# Plastid Phylogenomics of *Dendroseris* (Cichorieae; Asteraceae): Insights Into Structural Organization and Molecular Evolution of an Endemic Lineage From the Juan Fernández Islands

**DOI:** 10.3389/fpls.2020.594272

**Published:** 2020-11-05

**Authors:** Myong-Suk Cho, Seon-Hee Kim, JiYoung Yang, Daniel J. Crawford, Tod F. Stuessy, Patricio López-Sepúlveda, Seung-Chul Kim

**Affiliations:** ^1^Department of Biological Sciences, Sungkyunkwan University, Suwon-si, South Korea; ^2^Research Institute for Dok-do and Ulleung-do Island, Kyungpook National University, Daegu, South Korea; ^3^Department of Ecology and Evolutionary Biology and the Biodiversity Institute, The University of Kansas, Lawrence, KS, United States; ^4^Department of Evolution, Ecology, and Organismal Biology, The Ohio State University, Columbus, OH, United States; ^5^Department of Botany and Biodiversity Research, University of Vienna, Vienna, Austria; ^6^Departamento de Botánica, Universidad de Concepción, Concepción, Chile

**Keywords:** Asteraceae, adaptive radiation, plastome evolution, insular woodiness, Juan Fernández Islands, critically endangered

## Abstract

*Dendroseris* D. Don comprises 11 species endemic to the Juan Fernández islands in Chile. They demonstrate spectacular and unusual growth forms of rosette trees with extremely variable morphology and occupy wide ecological ranges on the islands. These unique plants are now highly threatened with extinction with very small population sizes, typically consisting of 10 or fewer individuals in wild. Despite morphological and ecological divergence among species of *Dendroseris*, their monophyly has been supported in previous studies, but with little resolution among subgeneric groups. We assembled seven complete plastome sequences from seven species of *Dendroseris*, including representatives from three subgenera, and carried out comparative phylogenomic analyses. The plastomes are highly conserved in gene content and order, with size ranging from 152,199 to 152,619 bp and containing 130 genes (87 coding genes, 6 rRNA genes, and 37 tRNA genes). Plastid phylogenomic analyses based on both the complete plastome sequences and 81 concatenated coding genes only show *Dendroseris* nested within *Sonchus s*ensu lato, and also that inter-subgeneric relationships are fully resolved. Subg. *Phoenicoseris* is resolved as sister to the remaining species of the genus and a sister relationship between the two subgenera *Dendroseris* and *Rea*. Ten mutation hotspots from LSC and SSC regions and variable SSRs are identified as potential chloroplast markers for future phylogenetic and phylogeographic studies of *Sonchus* and related groups.

## Introduction

The currently circumscribed *Sonchus* subg. *Dendroseris* (D. Don) S.-C. Kim & Mejías (tribe Cichorieae; Asteraceae) includes 12 highly threatened island endemics to the Juan Fernández and the Desventuradas Islands in the Pacific Ocean. The Juan Fernández archipelago is composed of three volcanic islands, i.e., Robinson Crusoe (also known as Masatierra, located about 667 km west of continental Chile; 48 km^2^), Alejandro Selkirk (also known as Masafuera, 181 km further westward, 50 km^2^), and Santa Clara (close to Robinson Crusoe, only 2 km^2^). Located 750 km north of the Juan Fernández archipelago, the Desventuradas Islands consist of the small islands of San Ambrosio and San Félix, along with several small islets.

For several decades, these 12 species in subg. *Dendroseris* have been recognized at the generic rank (*Dendroseris* D. Don and *Thamnoseris* F. Phil.) in subtribe Dendroseridinae Benth. (Stebbins, 1953; Bremer, 1994). However, recent molecular phylogenetic studies of *Sonchus* and related genera revealed that *Dendroseris* (comprising 11 species on the Juan Fernández archipelago) is deeply embedded within the genus *Sonchus* (Kim et al., 1996a, b, 2007). As a consequence, *Dendroseris* and *Thamnoseris* were merged in *Sonchus* as one of four recognized subgenera in a newly delimited subtribe Hyoseridinae Less (Lack, 2007; Kilian et al., 2009; Mejías and Kim, 2012). The genus *Sonchus* is an ideal group to study patterns and processes of plant evolution given its diverse morphological and life history traits (e.g., weedy annuals/biennials, herbaceous perennials, and woody perennials) and peculiar geographic distribution (e.g., Atlantic and Pacific Oceans, Africa, and cosmopolitan).

**FIGURE 1:**
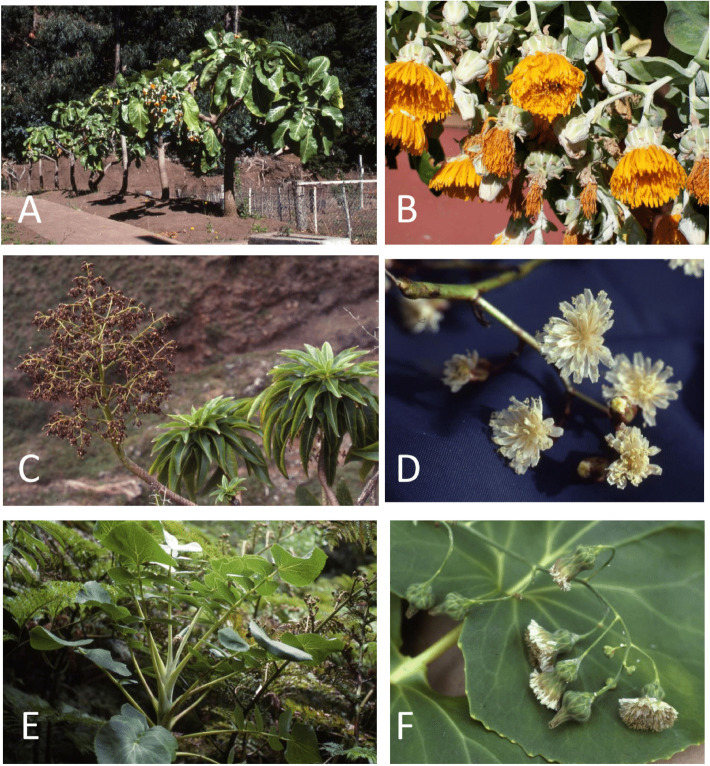
Species of *Dendroseris* from Robinson Crusoe Island in the Juan Fernández Archipelago, showing divergent morphological features. **(A,B)** *D. litoralis* (subg. *Dendroseris*); **(C,D)** *D. micrantha* (subg. *Rea*); **(E,F)** *D. berteroana* (subg. *Phoenicoseris*). Photo Credit: Tod F. Stuessy (Ohio State University, Columbus).

Regardless of its taxonomic rank, the *Dendroseris* lineage has attracted numerous taxonomists and evolutionary biologists for nearly a century. Hereafter, while the lineage is nested within *Sonchus*, we treat *Dendroseris* in the traditional sense as a distinc genus. Within the typically herbaceous family Asteraceae, *Dendroseris* shows spectacular and unusual tree-like growth-forms with extremely variable morphology and occupies wide ecological ranges on the islands (Figure 1 and Table 1). The spectrum of life form ranges from palmiform rosette trees to sparsely branched rosette trees and succulent rosette shrubs (Carlquist, 1967). Initially, this diversity in growth forms led Skottsberg (1953) to recognize four segregate genera (*Dendroseris*, *Rea*, *Hesperoseris*, and *Phoenicoseris*), but recognition of one cohesive genus is now followed by most workers (e.g., Wodehouse, 1935; Stebbins, 1953). Anatomical and molecular evidence provides support for a species-level classification that concur with the three traditionally recognized subgenera within *Dendroseris* (Carlquist, 1967; Sang et al., 1994). Subsequent treatments have recognized three subgenera; (1) subg. *Dendroseris* Skottsb., comprising the four species *D. litoralis* Skottsb., *D. macrantha* (Bertero ex Decne.) Skottsb., *D. macrophylla* D. Don and *D. marginata* Hook. & Arn., (2) subg. *Phoenicoseris* Skottsb., with the three species *D. berteroana* (Decne.) Hook. & Arn., *D. pinnata* (Bertero ex Decne.) Hook. & Arn., and *D. regia* Skottsb., and (3) subg. *Rea* (Bertero ex Decne.) Skottsb., consisting of the four species *D. gigantea* Johow, *D. micrantha* Hook. & Arn., *D. neriifolia* Hook. & Arn., and *D. pruinata* (Johow) Skottsb. (Stuessy et al., 1984; Sanders et al., 1987; Crawford et al., 1992, 1998). These unique plants are now highly threatened with extinction, as they are exceedingly rare with very small population sizes in the wild (almost always fewer than 10 plants) and widely scattered (Skottsberg, 1953; Stuessy et al., 1998). Faced with the threats of extinction, all 11 *Dendroseris* species on the Juan Fernández archipelago have been categorized as Critically Endangered in the wild, CE BI +2c, on the IUCN Red List of Threatened Species (Walter and Gillett, 1998).

**TABLE 1:** Morphological and ecological characteristics of seven *Dendroseris* species sequenced and analyzed in this study.

	Subg. *Dendroseris*			Subg. *Rea*		Subg. *Phoenicoseris*	
Species	*D. litoralis*	*D. macrantha*	*D. marginata*	*D. pruinata*	*D. micrantha*	*D. berteroana*	*D. pinnata*
Habit	Branched rosette tree with solid stems			Branched rosette shrub with solid and diaphramed stem		Palmiform with hollow stem	
Inflorescence	Heads few, large (3–5 mm wide), orange			Heads many, small, whitish		Heads many, small, whitish	
Achene	Winged, large, thick-walled			Non-winged, small, thin-walled		Non-winged, small, thin-walled	
Leaves	Entire, thin to thick, glabrous			Entire, elongate, very thick, glabrous		Pinnate, elongate, thick, glabrous	
Locality and habitat	Santa Clara and small rock off the south coast of Robinson Crusoe Island, coastal cliff, among coastal rocks in low islet	Robinson Crusoe Island in moist forest	Robinson Crusoe Island, cliffs at higher elevations	Robinson Crusoe Island and Santa Clara, open areas, either at low elevations near coast, or windy cliffs at higher elevations	Robinson Crusoe Island, common in the east and central parts, in forests and thickets at middle elevations, 400–600 m alt	Robinson Crusoe Island, in wet forest (extreme shade and moisture), tree/fern forests at higher altitudes	Robinson Crusoe Island, higher ridges, at the limit of the forest, among shrubs and small trees

*Dendroseris* on the Juan Fernández Islands has been the subject of numerous systematic and biogeographic studies over several decades as one of the most striking examples of adaptive radiation on oceanic islands in the Pacific Ocean (e.g., Skottsberg, 1953, 1956; Carlquist, 1967; Sanders et al., 1983, 1987; Crawford et al., 1987, 1992; Sang et al., 1994; Anderson et al., 2001; Bernardello et al., 2001). Despite the morphological, anatomical, and ecological divergence, *Dendroseris* has been strongly supported as a monophyletic group by several molecular markers (Sanders et al., 1983, 1987; Crawford et al., 1987, 1992; Spooner et al., 1987; Pacheco et al., 1991; Sang et al., 1994; Kim et al., 1996a, b; Esselman et al., 2000). Although the monophyly of *Dendroseris* was well-established, phylogenetic relationships among the three traditionally recognized taxonomic groups and species remained uncertain probably due to their rapid speciation. In addition, the phylogenetic position of *Dendroseris* relative to other *Sonchus* groups has been elusive (Sang et al., 1994; Kim et al., 1996a, b, 1999, 2007). While the two subgenera *Dendroseris* and *Phoenicoseris* were resolved as monophyletic, subg. *Rea* was not and relationships among the three subgenera were unresolved based on chloroplast (cpDNA) restriction fragment length polymorphisms (RFLPs), nuclear ribosomal DNA (nrDNA) internal transcribed spacer (ITS) sequences (Crawford et al., 1992; Sang et al., 1994), and allozymes (Crawford et al., 1998; Esselman et al., 2000). The neighbor-joining (NJ) tree based on randomly amplified polymorphic DNAs (RAPDs) showed the monophyly of each three subgenera, without further resolutions among them (Esselman et al., 2000). Lastly, the phylogenetic position of *Dendroseris* within the *Sonchus* group could not be determined confidently (Kim et al., 1999, 2007). In particular, the partial use of several coding and non-coding plastid regions in previous studies have been insufficient to provide robust phylogenetic relationships among *Dendroseris* and other closely related *Sonchus* groups (Sang et al., 1994; Kim et al., 1996a, b, 1999, 2007; Lee et al., 2005).

Fortunately, massive amounts of data have now become available with the advent of high-throughput sequencing technologies of next-generation sequencing (NGS) has revealed considerable genome-wide variation in sequences and structures of entire plastid genomes. The benefits of genome-wide data have increased phylogenetic resolution and significantly enhanced our understanding of plant evolution and diversity in the field of plastid genetics and genomics (Daniell et al., 2016). Whole plastome sequencing is now an efficient option for increasing phylogenetic resolution at lower taxonomic levels that are currently hindered by limited sequence variation due to recent divergence, rapid radiation and conservative genome evolution of plastomes (Parks et al., 2009).

In this study, we sequenced and assembled the whole plastid genomes of seven species of *Dendroseris*, representing three subgenera (*Dendroseris*, *Rea*, and *Phoenicoseris*) on the Juan Fernández Islands. Based on the complete plastome sequences, we tested the previous phylogenetic hypotheses proposed by various molecular markers, specifically focusing on inter-subgeneric relationships within the genus. We also performed comparative plastome analyses based on the phylogenetic framework to determine the structure, gene content, and rearrangements in the plastid genomes. Furthermore, we wanted to identify highly variable plastid regions and microsatellites or simple sequence repeats (SSRs), which could be utilized as useful markers for further population genetic or phylogeograhic studies of *Dendroseris*.

## Materials and Methods

### Plant Materials and DNA Extraction

Plant materials of seven *Dendroseris* species were collected previously in the field during four expeditions of the Universidad de Concepción, Chile and the Ohio State University, United States to the Juan Fernández Islands. As acknowleged in the previous studies (Crawford et al., 1987, 1992; Pacheco et al., 1991), the CONAF (Corporación National Forestal) of Chile issued the permission to collect in the Robinson Crusoe National Park. Our current samples of *Dendroseris* are representatives of those previous collections. For outgroup *Reichardia ligulata*, we used the same material as previously studied (Kim et al., 1996b, 2007). The fresh leaves were either dried (placed in sealable plastic bags with silica gel) or placed on ice and retained at 4°C until extracted in the laboratory at The Ohio State University, Columbus, OH, United States. Total genomic DNAs were extracted using the CTAB technique of Doyle and Doyle (1987), and purified in CsCl/ethidium bromide gradient.

### Plastome Sequencing, Assembly, and Annotation

**FIGURE 2:**
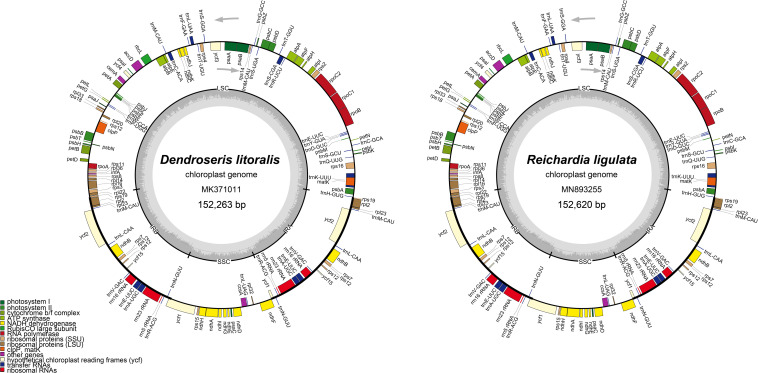
Gene maps of the plastid genomes of seven *Dendroseris* and *Reichardia ligulata* sequenced and analyzed in this study. The other six *Dendroseris* plastomes were consistent with *D. litoralis* in the structures and gene contents, but differed in their total length, having 152,263 bp for *D. macrantha* (MT157217), 152,261 bp for *D. marginata* (MT157218), 152,290 bp for *D. pinnata* (MT157219), 152,327 bp for *D. micrantha* (MK371008), 152,199 bp for *D. berteroana* (MK371014), and 152,348 bp for *D. pruinata* (MK371005). The genes inside and outside of the circle are transcribed in the clockwise and counterclockwise directions, respectively. Genes belonging to different functional groups are shown in different colors. The thick lines indicate the extent of the inverted repeats that separate the genomes into small single copy (SSC) and large single copy (LSC) regions.

Illumina paired-end (PE) genomic libraries with fragment size of 550 bp were prepared and sequenced using the Illumina HiSeq platform (Illumina, Inc., San Diego, CA, United States) at Macrogen Corporation (Seoul, South Korea). The sequence contigs were assembled by the *de novo* genomic assembler, Velvet 1.2.10 (Zerbino and Birney, 2008) at coverage ranging from 429 to 1489x. Annotation was performed using the Dual Organellar GenoMe Annotator (Wyman et al., 2004), ARAGORN v1.2.36 (Laslett and Canback, 2004), and RNAmmer 1.2 Server (Lagesen et al., 2007). Using Geneious v8.1.6 (Biomatters, Ltd., Auckland, New Zealand), the draft annotation was inspected and corrected manually, performing blast search by comparison with homologous genes in *Lactuca sativa* (DQ383816), *S. canariensis* (NC042381), *S. acaulis* (NC042382), and *S. webbii* (NC042383) from the GenBank database at the National Center for Biotechnology Information (NCBI) as references. The complete plastome sequences were registered in GenBank under the accession numbers MN893255 (*R. ligulata* from Spain, Collection # TE); MK371014 (*D. berteroana*, Collection # 12125); MK371011 (*D. litoralis*, Collection # 6435); MT157218 (*D. marginata*, Collection # 12068); MK371008 (*D. micrantha*, Collection # 11944); MT157219 (*D. pinnata*, Collection # 11132); and MK371005 (*D. pruinata*, Collection # 5108). OGDRAW (Lohse et al., 2013) was used to draw circular plastid genome maps (Figure 2).

### Comparative Plastome Analyses and Identification of Highly Divergent Regions

We performed several comparative plastome analyses among *Dendroseris* and other closely related *Sonchus* species on the Canary Islands, Atlantic Ocean. The analyses also included two related *Sonchus* species (*S. asper* and *S. canariensis*). *Sonchus asper* is a globally distributed herbaceous weed and *S. canariensis* is an arborescent shrub endemic to the Canary Islands. Codon usage frequency was calculated by using MEGA7 (Kumar et al., 2016) with relative synonymous codon usage (RSCU) value, which is the relative frequency of occurrence of the synonymous codon for a specific amino acid. The online program predictive RNA editor for plants (PREP) suite (Mower, 2009) was used to predict the possible RNA editing sites for annotated protein-coding genes with 35 reference genes available with known edit sites, based on a cutoff value of 0.8 (suggested as optimal for PREP-Cp). Overall sequence divergence was estimated using the LAGAN alignment mode (Brudno et al., 2003) in mVISTA (Frazer et al., 2004). Nucleotide diversity (Pi) was calculated using the sliding window analysis (window length = 1000 bp and step size = 200 bp excluding sites with alignment gaps) to detect the most divergent regions (i.e., mutation hotspots) in DnaSP (Librado and Rozas, 2009).

### Repeat Sequence Analysis

Two types of repeat sequences were identified in the eight plastid genomes of seven species of *Dendroseris* and *R. ligulata*. REPuter (Kurtz et al., 2001) was used to detect the various types of repetitive sequences with search parameters set to: maximum computed repeats = 50, minimum repeat size = 8 bp, and hamming distance = 1. SSRs were identified using MISA web^1^ with search parameters of 1–15 (unit size-minimum repeats, i.e., mono-nucleotide motifs with 15 minimum numbers of repetition), 2–5, 3–3, 4–3, 5–3, and 6–3 with 100 interruption (maximum difference for two SSRs).

### Phylogenetic Analysis

Phylogenetic relationships of the newly sequenced accessions of *Dendroseris* were investigated with other closely related *Sonchus* species using *R. ligulata* as the outgroup. Seven representative complete plastid sequences belonging to the major lineages of *Sonchus* were obtained from GenBank, including the woody *Sonchus* alliance species (*Sonchus* subg. *Dendrosonchus*) in Macaronesian Islands and globally occurring herbaceous weedy species (*Sonchus* subg. *Sonchus*). In total, full sequences of 15 plastid genomes, including an outgroup taxon, *R. ligulata*, were aligned using MAFFT v.7 (Katoh and Standley, 2013). Maximum likelihood trees based on both the complete plastid genome sequences and the concatenated sequences of 81 coding genes (excluding six repeated in IR) were produced with 1000 replicate bootstrap (BS) analyses by IQ-TREE (Nguyen et al., 2014). The best fit evolutionary model was chosen as TVM + F + I, which was scored according to the Bayesian information criterion (BIC) scores and weights by testing 88 DNA models of ModelFinder (Kalyaanamoorthy et al., 2017) implemented in IQ-TREE.

## Results

### Gene Content, Order, and Organization of the Plastomes of Dendroseris

**FIGURE 3:**
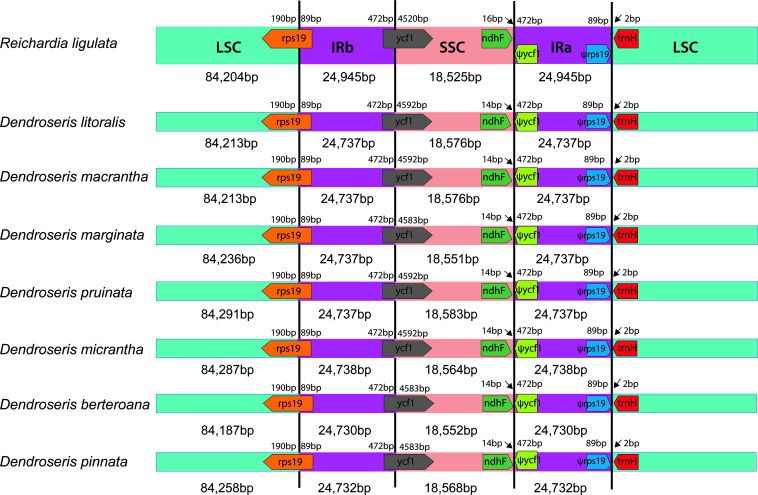
Comparison of the border positions of the large single copy (LSC), small single copy (SSC), and inverted repeat (IR) regions among seven *Dendroseris* and *Reichardia ligulata* plastid genomes. Gene names are indicated in boxes, and their lengths in the corresponding regions are displayed above the boxes. Ψ indicates a pseudogene.

Despite the great morphological and ecological differences among them, the seven plastomes of *Dendroseris* species and one outgroup taxon, *R. ligulata* were highly conserved in gene content and arrangement, displaying 99.3% pairwise similarity in sequences (99.8% among seven *Dendroseris* species only) (Figures 2, 3). Within *Dendroseris*, the total length of seven plastomes ranged from 152,199 (*D. berteroana;* subg. *Dendroseris)* to 152,348 (*D. pruinata;* subg. *Rea)* base pairs (bp), and consisted of four typical regions: large single copy (LSC), small single copy (SSC), and a pair of inverted repeat (IR) regions. The overall guanine-cytosine (GC) content of each plastid genome was 37.6%, with LSC, SSC, and IR regions having 35.7–35.8, 31.1–31.3, and 43.1% GC contents, respectively (Table 2). Each of the eight cp genomes contained 130 genes, including 87 protein-coding genes (excluding pseudogenes), six rRNA genes, and 37 tRNA genes (Table 3). Eighteen genes contained introns, including seven tRNA genes. Three genes of *clpP, rps12*, and *ycf3* exhibited two introns. The *trn*K-UUU tRNA gene harbored the largest intron, which contained the *mat*K gene in between. In total, 17 genes were duplicated in the IR regions, including seven tRNAs, three rRNAs, and seven protein genes. The trans-splicing gene *rps*12, consisting of three exons, was located in the LSC region for exon 1, but exon 2 and exon 3 of the gene were imbedded in the IR regions. Part of *ycf*1 and *rps*19 duplicated in IR region were annotated as pseudogenes in all cp genomes sequenced in this study.

**TABLE 2:** Genomic features of the complete plastid genomes of seven *Dendroseris* species and *Reichardia ligulata* sequenced and analyzed in this study.

Taxa	Accession number	Total plastid size (bp)/GC content (%)	LSC size (bp)/ GC content (%)	IR size (bp)/GC content (%)	SSC size (bp)/GC content (%)	No. of genes	No. of protein coding genes	No. of tRNA genes	No. of rRNA genes
***Reichardia* (outgroup)**									
*R. ligulata*	MN893255	152,620/37.6	84,205/35.7	24,945/43.1	18,525/31.1	130	87	37	6
***Dendroseris* subg. *Dendroseris***									
*D. litoralis*	MK371011	152,263/37.6	84,213/35.8	24,737/43.1	18,576/31.2	130	87	37	6
*D. macrantha*	MT157217	152,263/37.6	84,213/35.8	24,73743.1	18,576/31.2	130	87	37	6
*D. marginata*	MT157218	152,261/37.6	84,236/35.8	24,737/43.1	18,551/31.2	130	87	37	6
***Dendroseris* subg. *Rea***									
*D. pruinata*	MK371005	152,348/37.6	84,291/35.8	24,737/43.1	18,583/31.2	130	87	37	6
*D. micrantha*	MK371008	152,327/37.6	84,287/35.8	24,738/43.1	18,564/31.2	130	87	37	6
***Dendroseris* subg. *Phoenicoseris***									
*D. berteroana*	MK371014	152,199/37.6	84,187/35.8	24,730/43.1	18,552/31.3	130	87	37	6
*D. pinnata*	MT157219	152,290/37.6	84,258/35.8	24,732/43.1	18,568/31.2	130	87	37	6

*LSC, large single copy region; SSC, small single copy region; IR, inverted repeat.*

**TABLE 3:** Genes present in the complete plastid genomes of seven *Dendroseris* species and *R. ligulata* sequenced in this study.

Category	Gene name
Photosystem I	*psa*A, *psa*B, *psa*C, *psa*I, *psa*J, *ycf*3^*b*^, *ycf*4
Photosystem II	*psb*A, *psb*B, *psb*C, *psb*D, *psb*E, *psb*F, *psb*H, *psb*I, *psb*J, *psb*K, *psb*L, *psb*M, *psb*N, *psb*T, *psb*Z
Cytochrome b6/f complex	*pet*A, *pet*B^*a*^, *pet*D, *pet*G, *pet*L, *pet*N
Cytochrome C synthesis	*ccs*A
ATP synthase	*atp*A, *atp*B, *atp*E, *atp*F^*a*^, *atp*H, *atp*I
RuBisCO	*rbc*L
NADH oxidoreductase	*ndh*A^*a*^, *ndh*B^*a,d*^, *ndh*C, *ndh*D, *ndh*E, *ndh*F, *ndh*G, *ndh*H, *ndh*I, *ndh*J, *ndh*K
Large subunit ribosomal proteins	*rpl*2^*a,d*^, *rpl*14, *rpl*16^*a*^, *rpl*20, *rpl*22, *rpl*23^*d*^, *rpl*32, *rpl*33, *rpl*36
Small subunit ribosomal proteins	*rps*2, *rps*3, *rps*4, *rps*7^*d*^, *rps*8, *rps*11, *rps*12^*b,d,e*^, *rps*14, *rps*15, *rps*16^*a*^, *rps*18, *rps*19
RNA polymerase	*rpo*A, *rpo*B, *rpo*C1^*a*^, *rpo*C2
Translation initiation factor	*inf*A
Others	*acc*D, *cem*A, *clp*P^*b*^, *mat*K^*a*^
Unknown function genes (conserved reading frames)	*ycf*1, *ycf*2^*d*^, *ycf*15^*d*^
Ribosomal RNAs	*rrn*5^*d*^, *rrn*16^*d*^, *rrn*23^*d*^
Transfer RNAs	*trn*A-UGC^*a,d*^, *trn*C-ACA^*a*^, *trn*C-GCA, *trn*D-GUC, *trn*E-UUC, *trn*E-UUC^*a,d*^, *trn*F-GAA, *trn*G-GCC, *trn*H-GUG, *trn*K-UUU^*a*^, *trn*L-CAA^*d*^, *trn*L-UAA^*a*^, *trn*L-UAG, *trn*M-CAU^C,d^, *trn*N-GUU^*d*^, *trn*P-UGG, *trn*Q-UUG, *trn*R-ACG^*d*^, *trn*R-UCU, *trn*S-CGA^*a*^, *trn*S-GCU, *trn*S-GGA, *trn*S-UGA, *trn*T-GGU, *trn*T-UGU, *trn*V-GAC^*d*^, *trn*W-CCA, *trn*Y-GUA

*^*a*^Gene containing a single intron. ^*b*^Gene containing two introns. ^C^Two gene copies in LSC. ^*d*^Two gene copies in IRs. ^*e*^Trans-splicing gene.*

### Codon Usage and RNA Editing Sites

The frequency of codon usage was calculated based on the sequences of protein coding genes, of which RSCU (the relative frequencies of occurrence of the synonymous codon usages for a specific amino acid) values were reported in Supplementary Figure 1 (see Supplementary Table 1 for details). The 10 plastomes including seven *Dendroseris*, two *Sonchus* species (*S. asper* and *S. canariensis*), and outgroup taxon, *R. ligulata* showed very similar frequencies of codon usage despite morphological and evolutionary divergence among them. We found that all possible codons for all amino acids were used in their plastomes as specified in Supplementary Table 1. The highest RSCU value was in the usage of AGA codon for arginine (1.88) followed by UUA for leucine (1.81–1.82), while the lowest one, AGC for serine (0.34–0.35) followed by CUG for leucine (0.37–0.39) among all of them in common.

The total number of possible RNA editing sites predicted among 10 plastomes ranged from 93 to 104 sites in 35 protein-coding genes. Compared to the herbaceous species *S. asper* (98 sites) and *R. ligulata* (93 sites), most woody species of *Dendroseris* species and *S. canariensis* showed relatively more numbers of RNA edited sites (102–104 sites) except for *D. berteroana* (99 sites). The numbers of potential editing sites were not correlated with gene length; the highest numbers of potential editing sites were found in the *psa*B gene (10–12 sites), followed by the *ndh*B gene (9–10 sites). Only minor sites were predicted for longer genes, i.e., two sites for the longest gene *rpo*C2 and one for the second longest *rpo*B. Any potential RNA edited sites were not predicted for seven genes of *atp*F, *pet*D, *pet*G, *pet*L, *psa*I, *psb*B, and *psb*F consistently from all analyzed plastomes. *R. ligulata* differed at two more codon position in the *psa*B gene, from serine (S) to leucine (L), and from leucine (L) to phenylalanine (F), while *S. asper* changed one more codon position in the *ndh*B gene, from leucine (L) to phenylalanine (F) when compared with the rest of the species of *Dendroseris* and *Sonchus*. Most editing sites were distributed at the 2nd and 1st codon positions (Supplementary Table 2). The highest conversions in editing frequencies of codons associated with the corresponding amino acid changes were represented by the changes from proline (P) to leucine (L) (average score of 21.606) followed by serine (S) to leucine (L) (average score of 19.12) (Supplementary Figure 2).

### Sequence Divergence and Hotspot

**FIGURE 4:**
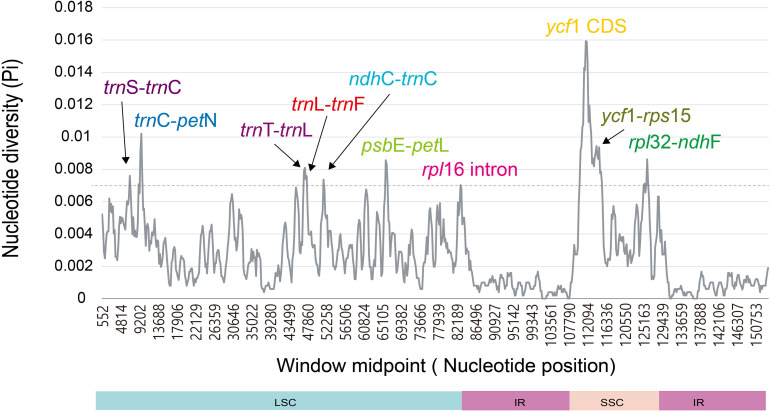
Ten most hotspot regions found in seven *Dendroseris*, two *Sonchus* (*S. asper*, and *S. canariensis*), and *Reichardia ligulata*.

The divergence level of nucleotide diversity was compared using DnaSP in both ways among seven *Dendroseris* plastomes only vs. among 10 plastomes including reference plastomes of *Sonchus* species (*S. asper* and *S. canariensis*) and the outgroup species *R. ligulata*. Overall nucleotide diversity value (Pi) among 10 plastomes (average value of 0.00283, ranging from 0 to 0.01593) was much higher than the one comparing seven *Dendroseris* plastid genomes (average value of 0.00061, ranging from 0 to 0.0039). The SSC regions, where the most variable gene, *ycf*1 was located, showed the highest nucleotide diversity in both analyses (0.00573 for 10 plastomes vs. 0.00121 for seven of *Dendroseris*), while the lowest value was in the IR boundary regions (0.00083 vs. 0.0003). Ten divergence hotspots among 10 plastomes were suggested as potential plastid markers for phylogenetic studies of *Dendroseris* species and closely related *Sonchus* groups. Eight intergenic regions (*trn*S-*trn*C, *trn*C-*pet*N, *trn*T-*trn*L, *trn*L-*trn*F, *ndh*C-*trn*C, *psb*E-*pet*L, *ycf*1-*rps*15, and *rpl*32-*ndh*F), one intron region (*rpl*16 intron), and one protein coding region (*ycf*1) were found in LSC and SSC regions (Figure 4). Additionally, seven hot spots identified among *Dendroseris* plastomes were found in LSC and SSC regions (Supplementary Figure 3).

**FIGURE 5:**
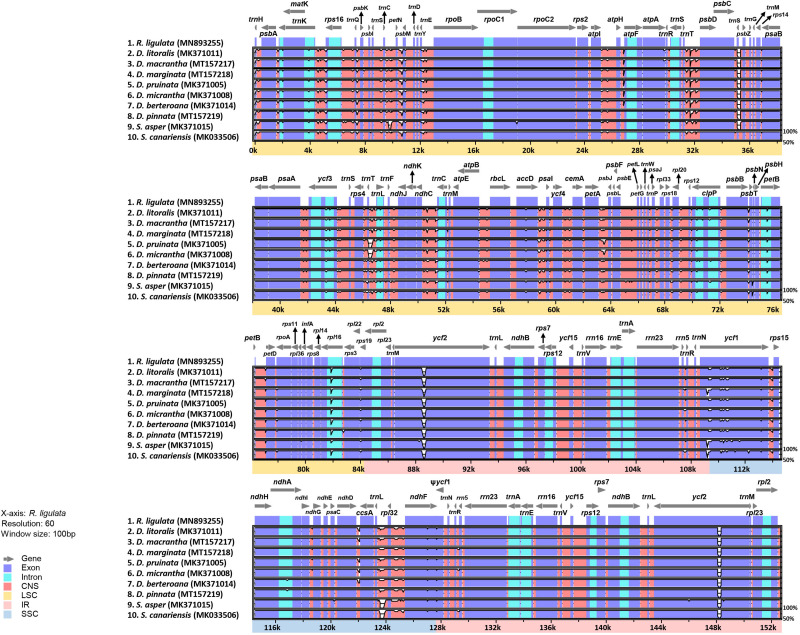
Comparison of the plastid genomes of seven *Dendroseris* and two *Sonchus* species, against *R. ligulata* by mVISTA. Gray arrows indicate genes with their orientation and position. Genome regions are color-coded as blue blocks for the conserved coding genes (exon), aqua blue blocks for introns, and orange blocks for the conserved non-coding sequences in intergenic regions (CNS). Thick lines below the alignment indicate the quadripartite regions of genomes; LSC region is in beige, IR regions, in pink, and SSC region, in light blue.

The result of mVISTA plotted against *R. ligulata* also exhibited a high degree of synteny and gene order conservation among the plastomes of *Dendroseris* and *Sonchus* species (Figure 5). A total of 1,873 polymorphic sites, which were identified in the DnaSP analysis, were visualized in mVISTA graph from mostly non-coding and intron regions, but also from several protein coding regions. The divergent coding genes of *Dendroseris* and *Sonchus* species against *R. ligulata* were *atp*A, *acc*D, *rpo*A, *ycf*2, *ycf*1, and *ndh*F.

### SSRs and Large Repeat Sequences

**FIGURE 6:**
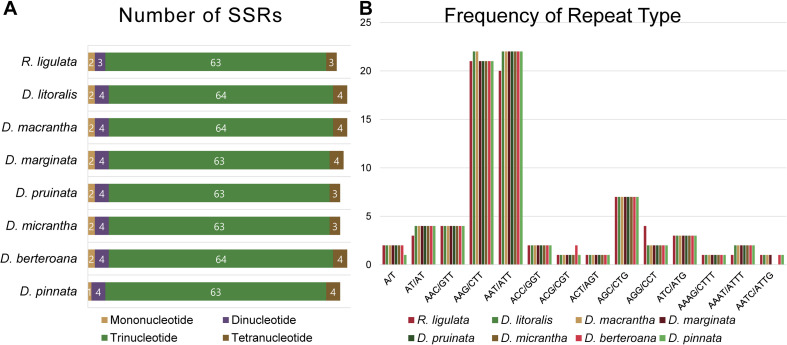
Simple sequence repeat (SSR) number per distribution and repeat type of the plastid genomes of seven *Dendroseris* and *Reichardia ligulata*. **(A)** Variation in the numbers of SSRs per distribution pattern. **(B)** Number of SSR motifs in different repeat motifs.

**FIGURE 7:**
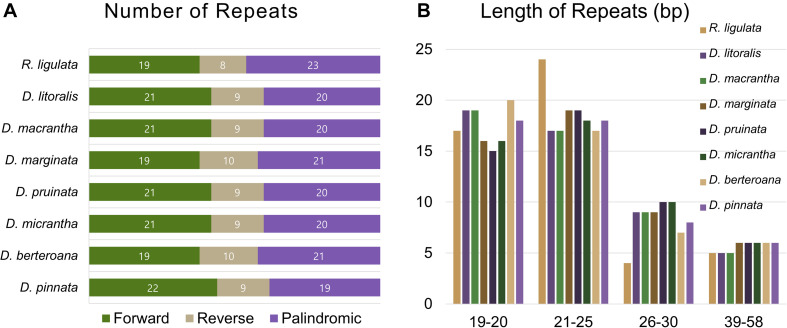
Repeat numbers per repeat type and repeat length of eight plastid genomes of seven *Dendroseris* and *Reichardia ligulata*. **(A)** Variation in the distribution of forward, reverse, complement, and palindromic repeats. **(B)** Number of different repeat lengths.

All of the eight plastid genomes of *Dendroseris* and *R. ligulata* contained very similar numbers and distribution patterns of repeated sequences. There were 71–74 SSRs detected by MISA based on search parameters set for 1–15 (mono-nucleotide motifs with 15 minimum numbers of repetition), 2–5, 3–3, 4–3, 5–3, and 6–3. The vast majority of the SSRs were tri-nucleotide motifs (63–64 SSRs, 87%), while other repeat types were relatively fewer; one to two mono-nucleotide motifs (3%), three to four di-nucleotide motifs (5%), and three to four tetra-nucleotide motifs (5%) (Figure 6A). The most abundant repeat motif was ‘AAT/ATT’ (30%) followed by ‘AAG/CTT’ (29%) in all eight genomes (Figure 6B and Supplementary Table 3). Interestingly, SSRs were distributed most frequently in the coding regions (53%), followed by intergenic regions (42%), with much lower numbers found in the non-coding introns (5%) in each cp genome (Supplementary Table 4). The coding regions with highest number of SSRs were *ycf* genes; 10 SSRs (five duplicated in each IR) in *ycf*2 and two in *ycf*1 (in SSC). Considering the quadripartite regional occupancy of SSRs, the IR and SSC regions were lower in overall SSR frequency compared with the LSC region; 15% from the SSC region and 18% from each of both IR regions versus 62% from the LSC region (Supplementary Table 4). Additionally, we found 50 pairs of large repeats in each cp genome (excluding duplicated IR region) using the parameters of maximum computed repeats = 50, minimum repeat size = 8 bp, and hamming distance = 1 by REPuter. They contained 19–22 forward, 8–10 reverse, and 19–23 palindromic matches of repeats (Figure 7A). Most of these large repeats were present in the intergenic spacers, but five repeat matches were found within the *ycf* coding genes, two in *ycf*1 and three in *ycf*2. Lengths of 21–25 repeats were the most frequent (37%) followed by lengths of 19–20 repeats (35%), while longer repeats of 26–30 (17%) and 39–58 (11%) were rarer than shorter ones (Figure 7B).

### Phylogenetic Analysis

**FIGURE 8:**
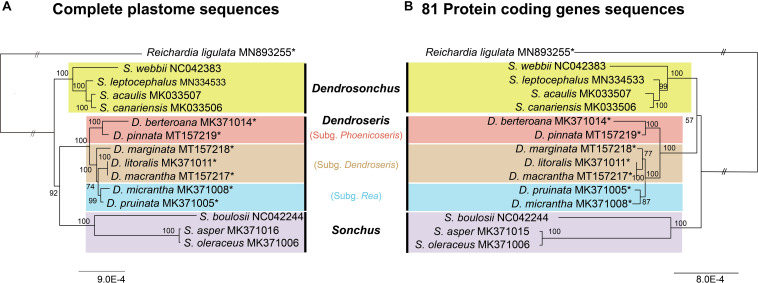
Maximum likelihood tree of *Dendroseris* and closely related *Sonchus* based on the complete plastome sequences **(A)** and 81 concatenated protein-coding genes **(B)**. Numbers above nodes are bootstrap values with 1000 replicates. The double slash (“//”) indicates that the branch lengths between outgroup and ingroup are shortened by one third size to improve readability of trees. Newly sequenced eight plastid genomes in this study are marked with an asterisk (*).

The complete plastome sequences provide good resolution of inter-subgeneric and interspecifc relationships within *Dendroseris* as well as resolving relationships among three major lineages within *Sonchus* sensu lato (Figure 8). Two maximum likelihood (ML) trees based on the complete sequences and the protein-coding genes only confirmed the monophyly of *Dendrosonchus*, *Dendroseris*, and *Sonchus* (100% BS) (Figure 8). However, phylogenetic relationships among three lineages within *Sonchus* sensu lato and within *Dendroseris* were unresolved between two ML trees. The complete plastome sequences suggested sister relationship between *Dendroseris* and *Sonchus* (92% BS; Figure 8A), while protein-coding genes showed sister relationships between *Dendroseris* and *Dendrosonchus* (56%; Figure 8B). In terms of relationships within *Dendrosonchus* from the Canary Islands, both trees showed that herbaceous perennial species with tuberous roots, *S. webbii*, diverged first within the group and also that pachycaulous *S. acaulis* is sister to the tree species *S. canariensis* (Figure 8). Within *Sonchus*, the newly described herbaceous perennial species *S. boulosii* from Morocco is sister to the cosmopolitan herbaceous weedy species *S. asper* and *S. oleraceus*.

## Discussion

### Highly Conserved Plastome Organization and Evolution in *Dendroseris*

The whole plastid genomes of *Dendroseris* species reported for the first time in this study were highly conserved structurally, sharing most common genomic features such as sequence similarity, gene content and numbers, and distributions of repeated sequences despite their morphological and ecological divergences. Interestingly, their genomic features are also very similar to other *Sonchus* groups with drastically different habits or geographical distribution (i.e., the woody *Sonchus* alliance of *Dendrosonchus* in the Macaronesian Islands, Atlantic Ocean and the globally distributed weedy sow thistles of *Sonchus* sensu stricto). Minor differences were found only in the total length of the herbaceous weedy species (*Sonchus s. str*.), which were shorter (151,808 ∼ 151,849 bp) than the woody shrubs and trees of *Dendrosonchus* (152,071 ∼ 152,194 bp) and *Dendroseris* (152,199 ∼ 152,620 bp), the differences due mainly to the SSC and LSC sizes (Cho et al., 2019a, b). Generally, the length of the plasid genome and its quadripartite regions varies among plant lineages due to the contraction and expansion of the inverted repeat regions. Evaluating their contraction and expansion by comparing the location of the boundaries among the four regions can provide some insights into plastid evolution (Menezes et al., 2018). The lengths of inverted repeats in the plastids of *Dendroseris, Dendrosonchus, and Sonchus s. str.* were very similar as a result of the complete duplication of: the genes *rpl*2, *rpl*23, *ycf*2, *ycf*15, *ndh*B, *rps*7; exons 1 and 2 of *rps*12; all three rRNA genes (5S, 16S, and 23S); and seven tRNA genes (*trn*L^CAA^, *trn*M^CAU^, *trn*V^*GAC*^, *trn*A^*UGC*^, *trn*E^*UUC*^, *trn*R^*ACG*^, and *trn*N^*GUU*^). Furthermore, the boundaries among the four regions IRs, LSC and SSC were fairly conserved, sharing exactly the same genes and similar gene contents at all junctions. The SSC region has been shown to exist in two equimolar states within individual plants differing only in the relative orientation of their single copy sequences (Palmer, 1983). Since the orientation of the SSC region is not standardized in GenBank (Walker et al., 2014, 2015), we reported one particular orientation of the SSC region for the comparative analysis in *Dendroseris, Dendrosonchus*, and *Sonchus.* All of them contained the functional protein coding gene of *ycf*1^ψ^ at IR/SSC with its pseudogene copy, *ycf*1^ψ^ at SSC/IR, and functional *rps*19 at LSC/IR with pseudogene copy *rps*19^Ψ^ at IR/LSC endpoints (Figure 3). Such a high conservation of plastid organization further corroborates that they share the most recent common ancestors, which has been previously suggested (Kim et al., 1996a, b, 2007; Kilian et al., 2009).

The frequency of codon usage vary by factors in species-specific ways, showing different preferences for the codons used to encode specific amino acids, probably as the result of evolution in the presence of mutational biases, selection for translation rate and accuracy, and possibly other factors (Orešiè and Shalloway, 1998). The patterns of frequently used codons were the same among *Dendroseris, Dendrosonchus*, and *Sonchus*, and only showed slightly different RSCU (the relative frequencies of occurrence of the synonymous codon usages for a specific amino acid) values. RNA editing alters plastid transcripts by converting specific cytidines to uridines, which results in a change in the amino acid sequence of the translated protein (Mower, 2009). In this study, several protein-coding genes showed variances in RNA editing prediction. The herbaceous weedy *S. asper* displayed less RNA editing at a total of 98 sites, especially for the genes *rpl*20 and *mat*K, compared to the woody *Dendrosonchus* species, *S. canariensis* (104 sites), and *Dendroseris* species (ranging from 99 to 104 sites). The dissimilarity in the RNA editing of the cosmopolitan weedy *S. asper* may originate from its different growth habit, but this needs further confirmation based on wider sampling because the woody species of *Dendroseris* on the Juan Fernández Islands exhibited quite diverse RNA editing prediction including *D. berteroana* (99 sites) with very similar RNA editing as herbaceous *S. asper.* As for SSRs and large repeat sequences, *Dendroseris* showed patterns comparable to *Dendrosonchus* and *Sonchus* in the numbers and frequencies of repeat type (Cho et al., 2019a, b). In addition, all showed an abundance of tri-nucleotide SSRs (over 80%), which is also consistent with other weedy Asteraceae species such as *Ambrosia trifida* (Sablok et al., 2019), and the highest frequencies of SSRs from the LSC region. Furthermore, the similar distribution pattern of large repeats was also observed for majority in forward (F) and palindromic (P) matches from *Dendroseris* (*F* = 19∼22, *P* = 19∼23 out of a total of 50 pairs) and herbaceous weedy *Sonchus* (*F* = 21, *P* = 21 out of total 49 pairs) (Cho et al., 2019b).

The nucleotide diversity (Pi value) was quite low within *Dendroseris* (average 0.00061) and *Dendrosonchus* (0.00090), and increased to 0.00117 for *Dendrosonchus* and *Sonchus s. str.*, and to 0.00283 for *Dendroseris*, *Dendrosonchus*, and *Sonchus* (Cho et al., 2019a, b). Given the highly conserved nature of the plastid genome in most angiosperms and the relatively recent origin of *Dendroseris* species on the Juan Fernández Islands (estimate ranging from 800,000 to 2.6 million years) (Crawford et al., 1992; Sang et al., 1994; Daniell et al., 2016), the low nucleotide diversity within *Dendroseris* is not surprising. Further, the overall patterns for highly variable regions were similar among the taxa. The most divergent hot spot was the *ycf*1 region followed by *rpl*32-*ndh*F, *trn*T-*trn*L-*trn*F, and *psb*E-*pet*L, which were suggested as potential cp markers for phylogenetic studies of *Sonchus* and closely related groups (Figure 4 and Supplementary Figure 3). Of 10 highly variable regions based on complete plastid genomes across diverse angiosperm lineages (Shaw et al., 2014), only three regions, i.e., *rpl*32-*trn*L, *psb*E-*pet*L, and *ndh*F-*rpl*32, were found to be also highly variable in *Dendroseris*. In this study, we identified variable molecular markers including SSRs and highly variable regions from plastid genomes, which will increase the efficiency and feasibility for species identification and phylogenetic reconstruction within *Sonchus*. The ML tree constructed from the concatenated sequences of 10 mutation hotspot regions (12,879 bp in length) demonstrated their effectiveness as potential molecular markers, and for resolving with high support values inter-subgeneric relationships within *Dendroseris* (except subg. *Rea*) as well as their relationships to other *Sonchus* groups (Supplementary Figure 4).

### Phylogenetic Relationships Within *Dendroseris*

Considering the overall high conservation among the plastomes of *Sonchus* and closely related species, it is not surprising that previous studies based on relatively few sequences from the plastid genomes provided limited resolution of relationships. Although *Dendroseris* was confirmed to be monophyletic in a *mat*K phylogeny, its relationships to other *Sonchus* groups and inter-subgeneric relationships within *Dendroseris* were not well-resolved (Kim et al., 2007). Here, the phylogenomic analyses of entire plastid sequences revealed better resolution among the three lineages sampled (i.e., *Dendrosonchus*, *Dendroseris*, and *Sonchus*). In agreement with earlier results, in the present study *Dendroseris* was nested deeply within the genus *Sonchus* sensu lato on both ML trees (Figures 8A,B). Therefore, *Dendroseris* was most likely derived from the *Sonchus* group, but its closest relatives or progenitors remain to be determined based on broader sampling of plastid sequences. Especially, to be desired are other Pacific Island endemics, and additional taxa of subg. *Sonchus* sections *Maritimi* and *Arvenses* because results of a prior study showed that species from these groups share the most recent common ancestor with *Dendroseris* (Kim et al., 2007). Since all *Dendroseris* have a diploid chromosome number of 36 (Sanders et al., 1983) and there is no evidence of multivalent formation (Sanders et al., 1983), suggestive of an autopolyploid (Jackson, 1982; Ramsey and Schemske, 2002), it can be considered allotetraploid. Given the higher frequency of polyploid *Sonchus* species in the Pacific Ocean compared to typically diploid ones in the Atlantic Ocean and Old World, the geographical origin of *Dendroseris* is likely somewhere in the Pacific Ocean (e.g., Australia, New Zealand, etc.) and adjacent regions (Kim et al., 2007). However, its origin is still enigmatic; it remains to be determined which lineages of *Sonchus* contributed to allotetraploid origin of *Dendroseris.*

Unlike the uncertain position of *Dendroseris* relative to other *Sonchus* groups, this study provides strong evidence of relationships among the three subgenera *Dendroseris*, *Rea*, and *Phoenicoseris*. Previous plastid phylogenies based on the *mat*K gene, the *psb*A-*trn*H intergenic spacer and restriction site mutations showed unresolved inter-subgeneric relationships, presumably the result of rapid radiation and speciation following the arrival of the common ancestor (Crawford et al., 1992; Kim et al., 1999, 2007). Other types of molecular markers, such as RAPDs (Esselman et al., 2000), ITS sequences (Sang et al., 1994), and allozymes (Crawford et al., 1998) failed to resolve relationships among the three subgenera. The initial divergence of subg. *Dendroseris* (*D. litoralis*, *D. marginata*, and *D. macrantha*) in the maximum likelihood tree based on ITS sequences is not significant, leaving inter-subgeneric relationships unresolved. However, species relationships within each subgenus have been strongly and consistently recognized regardless of different marker types. For example, a sister relationship between *D. litoralis* and *D. marginata* of subg. *Dendroseris* has been consistently recognized. Despite morphological and molecular similarities due to common ancestry, the species are ecologically and altitudinally well-differentiated: *D. litoralis* occurs in coastal lower elevations and *D. marginata* is found on higher elevation exposed cliffs (Stuessy et al., 1998). The second species pair of subg. *Rea*, *D. pruinata* and *D. micrantha*, also shows marked ecological and altitudinal differentiation: the former species commonly in middle elevation forests and the latter one in open lower coastal areas or open higher elevation windy cliffs (Sanders et al., 1987). Lastly, the species pair of subg. *Phoenicoseris*, *D. beteroana* and *D. pinnata*, are morphologically and ecologically quite divergent despite sharing the most recent common ancestor: the former in high altitude tree/fern forests and the latter in open wind-swept ridges at higher elevations. Therefore, all these species pairs suggest that spatial/ecological/altitudinal factors likely promoted the divergence and speciation of this largest and fascinating group on the Juan Fernández Islands.

The current whole plastid phylogenomic study shows the unresolved relationship of *D. marginata* within subg. *Dendroseris*. The phylogenetic tree suggests that *D. marginata* is either sister to the clade containing the subgenera *Dendroseris* and *Rea* (complete plastome sequence tree; Figure 8A) or is sister to the clade of *D. litoralis* and *D. macrantha* of subg. *Dendroseris* (protein coding genes only tree; Figure 8B). Given that the subg. *Dendroseris* is monophyletic based on various molecular markers and morphology, the tree based on protein coding genes only most likely represents the most plausible hypothesis of species relationships. In addition, our current study suggested that *D. litoralis* is more closely related to *D. macrantha* than to *D. marginata*. It has been demonstrated that, based on morphological traits, *D. macrantha* is most closely related to *D. macrophylla* on the geologically younger island Masafuera (Alejandro Selkirk), while flavonoid profiles provided no specific insights (Pacheco et al., 1991). The current study strongly suggests a sister relationship between *D. litoralis* and *D. macrantha* (100% BS). However, this relationship contrasts with morphological traits: *D. litoralis* has shorter morphological distance with *D. marginata* (two features) compared to the distance with *D. macrantha* (four features) (Sanders et al., 1987; Pacheco et al., 1991). It is probable that the close relationship of *D. litoralis* and *D. macrantha* based on the plastid genomes could be the result of past gene flow since *D. macrantha* is from cultivated material in the village and also morphology, especially leaf margin, is suggestive of gene flow event (TF Stuessy, personal observation).

Based on fully resolved inter-subgeneric relationships inferred in this study, we can hypothesize some processes with regard to the divergence and speciation of *Dendroseris* on the Juan Fernández Islands (Figure 8B). Upon arrival of the common ancestor on the older Robinson Crusoe island, two major lineages might have diverged; one lineage (subg. *Phoenicoseris*) toward higher elevation and the other lineage containing the two subgenera *Dendroseris* and *Rea* toward middle and lower elevations. Subgenus *Phoenicoseris* was considered to be highly derived within the genus based on morphology, with the characteristics of compound pinnatifid leaves, single-stemmed habit and being monocarpic (Sanders et al., 1987). In addition, subg. *Rea* was regarded as the most primitive subgenus based on morphological features and occurrence in middle elevation forests, and more open, arid habitats (Sanders et al., 1987). The current study, however, does not necessarily support the hypothesis that *Phoenicoseris* is highly derived, which was based on morphological features. Rather, the complete plastid sequences strongly suggest that after initial divergence of the two major lineages, the subg. *Phoenicoseris* lineage speciated at higher elevations with modification in some life history traits (e.g., monocarpic). A sister relationship between *Dendroseris* and *Rea* has never been postulated based on various molecular markers and, given their ecological preferences (*Dendroseris* in lower elevations along drier seacoasts and cliffs in full sun versus *Rea* in middle elevation in the edges of the cooler forests), it is plausible that the evolution of these two subgenera progressed from lower to middle elevations of the island.

## Conclusion

We characterized the first complete plastid sequences of seven species of *Dendroseris*, the largest endemic genus on the Juan Fernández Islands. As in most angiosperms, we found highly conserved plastomes at the generic level, including gene order and content. Despite the recent origin of *Dendroseris* species on the islands and the low rate of plastome evolution, we achieved the first fully resolved phylogeny for the genus. Especially, noteworthy was the complete resolution with strong support of relationships among the three subgenera. The plastid phylogenomics strongly suggest early divergence of two major lineages, one consisting of subg. *Phoenicoseris* and the other the clade comprised of subgenera *Dendroseris* and *Rea*. Although we achieved full resolution within the genus, questions remain such as the geographical origin within several lineages of *Sonchus* in the Pacific Ocean, the monophyly of subg. *Rea*, and the timing of the origin and radiation of the lineage in the archipelago. Our thorough characterization and comparative analyses among the plastid genomes have led us to discover several informative mutation hot spots and variable SSR regions, which can be used to identify and characterize each individual in these highly threatened and nearly extinct species of *Dendroseris*.

## Data Availability Statement

The datasets presented in this study can be found in online repositories. The names of the repository/repositories and accession number(s) can be found below: https://www.ncbi.nlm.nih.gov/genbank/, MN893255; https:// www.ncbi.nlm.nih.gov/genbank/, MK371011; https://www.ncbi. nlm.nih.gov/genbank/, MT157217; https://www.ncbi.nlm. nih.gov/genbank/, MT157218; https://www.ncbi.nlm.nih.gov/ genbank/, MK371005; https://www.ncbi.nlm.nih.gov/genbank/, MK371008; https://www.ncbi.nlm.nih.gov/genbank/, MK371014; https://www.ncbi.nlm.nih.gov/genbank/, MT157219.

## Author Contributions

M-SC and S-CK designed the experiment. DC, TS, and PL-S collected the samples. M-SC, S-HK, and JY performed the experiments and analyzed the data. M-SC drafted the manuscript. DC, TS, and S-CK revised the manuscript. All authors approved the final version of the manuscript.

## Conflict of Interest

The authors declare that the research was conducted in the absence of any commercial or financial relationships that could be construed as a potential conflict of interest.

## Footnotes

1 http://pgrc.ipk-gatersleben.de/misa/

## References

[B1] Anderson G. J. Bernardello G. Stuessy T. F. Crawford D. J. (2001). Breeding system and pollination of selected plants endemic to Juan Fernández Islands. *Am. J. Bot.* 88 220–233. 10.2307/2657013 11222245

[B2] Bernardello G. Anderson G. J. Stuessy T. F. Crawford D. J. (2001). A survey of floral traits, breeding systems, floral visitors, and pollination systems of the angiosperms of the Juan Fernández Islands (Chile). *Bot. Rev.* 67 255–308. 10.1007/bf02858097

[B3] Bremer K. (1994). *Plants and Islands.* Portland: Timber Press.

[B4] Brudno M. Do C. B. Cooper G. M. Kim M. F. Davydov E. Green E. D. (2003). NISC comparative sequencing program. LAGAN and multi-LAGAN: efficient tools for large-scale multiple alignment of genomic DNA. *Genome Res.* 13 721–731. 10.1101/gr.926603 12654723 PMC430158

[B5] Carlquist S. J. (1967). Anatomy and systematics of *Dendroseris* (sensu lato). *Brittonia* 19 99–121. 10.2307/2805268

[B6] Cho M. S. Kim J. H. Kim C. S. Mejías J. A. Kim S.-C. (2019b). Sow thistle chloroplast genomes: insights into the plastome evolution and relationship of two weedy species, *Sonchus asper* and *Sonchus oleraceus* (Asteraceae). *Genes* 10:881. 10.3390/genes10110881 31683955 PMC6895928

[B7] Cho M. S. Yang J. Y. Yang T. J. Kim S.-C. (2019a). Evolutionary comparison of the chloroplast genome in the woody *Sonchus* Alliance (Asteraceae) on the Canary Islands. *Genes* 10:217. 10.3390/genes10030217 30875850 PMC6470973

[B8] Crawford D. J. Sang T. Stuessy T. F. Kim S.-C. Silva M. O. (1998). “*Dendroseris* (Asteraceae: Lactuceae) and *Robinsonia* (Asteraceae: Senecioneae) on the Juan Fernandez islands: similarities and differences in biology and phylogeny,” in *Evolution and Speciation of Island Plants*, eds Stuessy T. Ono M. (New York, NY: Cambridge University Press), 97–119. 10.1017/cbo9780511721823.007

[B9] Crawford D. J. Stuessy T. F. Cosner M. B. Haines D. W. Silva M. O. Baeza M. (1992). Evolution of the genus *Dendroseris* (Asteraceae: Lactuceae) on the Juan-Fernandez Islands: evidence from chloroplast and ribosomal DNA. *Syst. Bot.* 17 676–682. 10.2307/2419735

[B10] Crawford D. J. Stuessy T. F. Silva M. O. (1987). Allozyme divergence and the evolution of *Dendroseris* (Compositae: Lactuceae) on the Juan Fernandez Islands. *Syst. Bot.* 12 435–443. 10.2307/2419268

[B11] Daniell H. Lin C. S. Yu M. Chang W. J. (2016). Chloroplast genomes: diversity, evolution, and applications in genetic engineering. *Genome Biol.* 17:134. 10.1186/s13059-016-1004-2 PMC4918201 27339192

[B12] Doyle J. J. Doyle J. L. (1987). A rapid DNA isolation procedure for small quantities of fresh leaf tissue. *Phytochem. Bull.* 19 11–15.

[B13] Esselman E. J. Crawford D. J. Brauner S. Stuessy T. F. Anderson G. J. Silva M. O. (2000). RAPD marker diversity within and divergence among species of *Dendroseris* (Asteraceae: Lactuceae). *Am. J. Bot.* 87 591–596. 10.2307/2656603 10766731

[B14] Frazer K. A. Pachter L. Poliakov A. Rubin E. M. Dubchak I. (2004). VISTA: computational tools for comparative genomics. *Nucleic Acids Res.* 32 W273–W279. 15215394 10.1093/nar/gkh458 PMC441596

[B15] Jackson R. C. (1982). Polyploidy and diploidy: new perspectives on chromosome pairing and its evolutionary implications. *Am. J. Bot.* 69 1512–1523. 10.1002/j.1537-2197.1982.tb13400.x

[B16] Kalyaanamoorthy S. Minh B. Q. Wong T. K. von Haeseler A. Jermiin L. S. (2017). ModelFinder: fast model selection for accurate phylogenetic estimates. *Nat. Methods* 14 587–589. 10.1038/nmeth.4285 28481363 PMC5453245

[B17] Katoh K. Standley D. M. (2013). MAFFT multiple sequence alignment software version 7: improvements in performance and usability. *Mol. Biol. Evol.* 30 772–780. 10.1093/molbev/mst010 23329690 PMC3603318

[B18] Kilian N. Gemeinholzer B. Lack W. L. (2009). “Cichorieae,” in *Systematics, Evolution and Biogeography of Compositae*, eds Funk V. A. Susanna A. Stuessy T. F. Bayer R. J. (Vienna: International Association for Plant Taxonomy), 343–383.

[B19] Kim S.-C. Crawford D. J. Francisco-Ortega J. Santos-Guerra A. (1996a). A common origin for woody *Sonchus* and five related genera in the Macaronesian islands: molecular evidence for extensive radiation. *Proc. Natl. Acad. Sci. U.S.A.* 93 7743–7748. 10.1073/pnas.93.15.7743 8755546 PMC38818

[B20] Kim S.-C. Crawford D. J. Jansen R. K. (1996b). Phylogenetic relationships among the genera of the subtribe Sonchinae (Asteraceae): evidence from ITS sequences. *Syst. Bot.* 21 417–432. 10.2307/2419668

[B21] Kim S.-C. Crawford D. J. Jansen R. K. Santos-Guerra A. (1999). The use of a non-coding region of chloroplast DNA in phylogenetic studies of the subtribe Sonchinae (Asteraceae: Lactuceae). *Plant Syst. Evol.* 215 85–99.

[B22] Kim S.-C. Lee C. Mejías J. A. (2007). Phylogenetic analysis of chloroplast DNA *mat*K gene and ITS of nrDNA sequences reveals polyphyly of the genus *Sonchus* and new relationships among the subtribe Sonchinae (Asteraceae: Cichorieae). *Mol. Phylogenet. Evol.* 44 578–597. 10.1016/j.ympev.2007.03.014 17531507

[B23] Kumar S. Stecher G. Tamura K. (2016). MEGA7: molecular evolutionary genetics analysis version 7.0 for bigger datasets. *Mol. Biol. Evol.* 33 1870–1874. 10.1093/molbev/msw054 27004904 PMC8210823

[B24] Kurtz S. Choudhuri J. V. Ohlebusch E. Schleiermacher C. Stoye J. Giegerich R. (2001). REPuter: the manifold applications of repeat analysis on a genomic scale. *Nucleic Acids Res.* 29 4633–4642. 10.1093/nar/29.22.4633 11713313 PMC92531

[B25] Lack H. W. (2007). “Cichorieae,” in *The Families and Genera of Vascular Plants, vol. 8, Flowering Plants, Eudicots, Asterales*, eds Kadereit J. W. Jeffrey C. (Berlin: Springer), 180–199.

[B26] Lagesen K. Hallin P. Rødland E. A. Stærfeldt H. H. Rognes T. Ussery D. W. (2007). RNammer: consistent annotation of rRNA genes in genomic sequences. *Nucleic Acids Res.* 35 3100–3108. 10.1093/nar/gkm160 17452365 PMC1888812

[B27] Laslett D. Canback B. (2004). ARAGORN, a program to detect tRNA genes and tmRNA genes in nucleotide sequences. *Nucleic Acids Res.* 32 11–16. 10.1093/nar/gkh152 14704338 PMC373265

[B28] Lee C. Kim S.-C. Lundy K. Santos−Guerra A. (2005). Chloroplast DNA phylogeny of the woody *Sonchus* alliance (Asteraceae: Sonchinae) in the Macaronesian Islands. *Am. J. Bot.* 92 2072–2085. 10.3732/ajb.92.12.2072 21646124

[B29] Librado P. Rozas J. (2009). DnaSP v5: a software for comprehensive analysis of DNA polymorphism data. *Bioinformatics* 25 1451–1452. 10.1093/bioinformatics/btp187 19346325

[B30] Lohse M. Drechsel O. Kahlau S. Bock R. (2013). OrganellarGenomeDRAW—A suite of tools for generating physical maps of plastid and mitochondrial genomes and visualizing expression data sets. *Nucleic Acids Res.* 41 W575–W581. 23609545 10.1093/nar/gkt289 PMC3692101

[B31] Mejías J. A. Kim S.-C. (2012). Taxonomic treatment of Cichorieae (Asteraceae) endemic to the Juan Fernández and Desventuradas Islands (SE Pacific). *Ann. Bot. Fenn.* 49 171–178. 10.5735/085.049.0303

[B32] Menezes A. P. A. Resende-Moreira L. C. Buzatti R. S. O. Nazareno A. G. Carlsen M. Lobo F. P. (2018). Chloroplast genomes of *Byrsonima* species (Malpighiaceae): comparative analysis and screening of high divergence sequences. *Sci. Rep.* 8 1–12. 29396532 10.1038/s41598-018-20189-4 PMC5797077

[B33] Mower J. P. (2009). The PREP suite: predictive RNA editors for plant mitochondrial genes, chloroplast genes and user-defined alignments. *Nucleic Acids Res.* 37 W253–W259. 10.1016/0168-9452(90)90180-v 19433507 PMC2703948

[B34] Nguyen L. T. Schmidt H. A. von Haeseler A. Minh B. Q. (2014). IQ-TREE: a fast and effective stochastic algorithm for estimating maximum-likelihood phylogenies. *Mol. Biol. Evol.* 32 268–274. 10.1093/molbev/msu300 25371430 PMC4271533

[B35] Orešiè M. Shalloway D. (1998). Specific correlations between relative synonymous codon usage and protein secondary structure. *J. Mol. Biol.* 281 31–48. 10.1006/jmbi.1998.1921 9680473

[B36] Pacheco P. Crawford D. J. Stuessy T. F. Silva M. O. (1991). Flavonoid evolution in *Dendroseris* (Compositae, Lactuceae) from the Juan Fernandez Islands, Chile. *Am. J. Bot.* 78 534–543. 10.1002/j.1537-2197.1991.tb15220.x

[B37] Palmer J. D. (1983). Chloroplast DNA exists in two orientations. *Nature* 301 92–93. 10.1038/301092a0

[B38] Parks M. Cronn R. Liston A. (2009). Increasing phylogenetic resolution at low taxonomic levels using massively parallel sequencing of chloroplast genomes. *BMC Biol.* 7:84. 10.1186/1741-7007-7-84 19954512 PMC2793254

[B39] Ramsey J. Schemske D. W. (2002). Neopolyploidy in flowering plants. *Annu. Rev. Ecol. Evol. Syst.* 33 589–639. 10.1146/annurev.ecolsys.33.010802.150437

[B40] Sablok G. Amiryousefi A. He X. Hyvönen J. Poczai P. (2019). Sequencing the plastid genome of giant ragweed (*Ambrosia trifida*, Asteraceae) from a herbarium specimen. *Front. Plant Sci.* 10:218. 10.3389/fpls.2019.00218 30873197 PMC6403193

[B41] Sanders R. W. Stuessy T. F. Marticorena C. Silva M. O. (1987). Phytogeography and evolution of *Dendroseris* and *Robinsonia*, tree-Compositae of the Juan Fernandez Islands. *Opera Bot.* 92 195–215.

[B42] Sanders R. W. Stuessy T. F. Rodriguez R. (1983). Chromosome numbers from the flora of the Juan Fernandez Islands. *Am. J. Bot.* 70 799–810. 10.1002/j.1537-2197.1983.tb06415.x

[B43] Sang T. Crawford D. J. Kim S.-C. Stuessy T. F. (1994). Radiation of the endemic genus *Dendroseris* (Asteraceae) on the Juan Fernandez Islands: evidence from sequences of the ITS regions of nuclear ribosomal DNA. *Am. J. Bot.* 81 1494–1501. 10.1002/j.1537-2197.1994.tb15635.x

[B44] Shaw J. Shafer H. L. Leonard O. R. Kovach M. J. Schorr M. Morris A. B. (2014). Chloroplast DNA sequence utility for the lowest phylogenetic and phylogeographic inferences in angiosperms: the tortoise and the hare IV. *Am. J. Bot.* 101 1987–2004. 10.3732/ajb.1400398 25366863

[B45] Skottsberg C. (1953). “The vegetation of the Juan Fernandez Islands,” in *The Natural History of Juan Fernandez and Easter Island V.2*, ed. Skottsberg C. (Uppsala: Almqvist and Wiksells boktryckeri), 793–960.

[B46] Skottsberg C. (1956). “Derivation of the Flora and Fauna of Juan Fernandez and Easter Island,” in *The Natural History of Juan Fernandez and Easter Island V.1*, ed. Skottsberg C. (Uppsala: Almqvist and Wiksells boktryckeri), 193–427.

[B47] Spooner D. M. Stuessy T. F. Crawford D. J. Silva M. O. (1987). Chromosome numbers from the flora of the Juan Fernandez Islands. II. *Rhodora* 89 351–356.

[B48] Stebbins G. L. (1953). A new classification of the tribe Cichorieae, family Compositae. *Madroño* 12 65–81.

[B49] Stuessy T. F. Sanders R. W. Silva M. O. (1984). “Phytogeography and evolution of the Juan Fernandez Islands: a progress report,” in *Pacific basin biogeography*, eds Radovsky F. J. Raven P. H. Sohmer S. H. (Lawrence, KS: B. P. Bishop Museum), 55–69.

[B50] Stuessy T. F. Swenson U. Crawford D. J. Anderson G. (1998). Plant conservation in the Juan Fernandez archipelago, Chile. *Aliso* 16 89–101. 10.5642/aliso.19971602.04

[B51] Walker J. F. Zanis M. J. Emery N. C. (2014). Comparative analysis of complete chloroplast genome sequence and inversion variation in *Lasthenia burkei* (Madieae, Asteraceae). *Am. J. Bot.* 101 722–729. 10.3732/ajb.1400049 24699541

[B52] Walker J. F. Zanis M. J. Emery N. C. (2015). Correction to “Comparative analysis of complete chloroplast genome sequence and inversion variation in *Lasthenia burkei* (Madieae, Asteraceae)”. *Am. J. Bot.* 102 1008–1008. 10.3732/ajb.1500990 26101424

[B53] Walter K. S. Gillett H. J. (1998). *1997 IUCN Red List of Threatened Plants.* Gland: IUCN-The World Conservation Union.

[B54] Wodehouse R. P. (1935). *Pollen Grains, Their Structure, Identification and Significance in Science and Medicine.* London: McGraw-Hill Book Company, Inc.

[B55] Wyman S. K. Jansen R. K. Boore J. L. (2004). Automatic annotation of organellar genomes with DOGMA. *Bioinformatics* 20 3252–3255. 10.1093/bioinformatics/bth352 15180927

[B56] Zerbino D. R. Birney E. (2008). Velvet: algorithms for de novo short read assembly using de Bruijn graphs. *Genome Res.* 18 821–829. 10.1101/gr.074492.107 18349386 PMC2336801

