# Supplementary material for: Plastid Phylogenomics of Dendroseris (Cichorieae; Asteraceae): Insights Into Structural Organization and Molecular Evolution of an Endemic Lineage From the Juan Fernández Islands
Source: Front Plant Sci. 2020 Nov 5;11:594272. doi: 10.3389/fpls.2020.594272 (PMC7674203; doi:10.3389/fpls.2020.594272)
Supplement: Supplementary file 1 [file Data_Sheet_1.zip › Table 1 - 2020-10-14T160845.760.DOCX]

Supplementary Material

Plastid phylogenomics of *Dendroseris* (Cichorieae; Asteraceae), endemic to the Juan Fernández Islands: Insights into structural organization and molecular evolution

**Myong-Suk Cho^1^, Seon-Hee Kim^1^, JiYoung Yang^2^, Daniel J. Crawford^3^, Tod F. Stuessy^4^, Patricio López-Sepúlveda^5^, and Seung-Chul Kim^1*^**

*** Correspondence**: Seung-Chul Kim: [sonchus96@skku.edu](mailto:sonchus96@skku.edu) or [sonchus2009@gmail.com](mailto:sonchus2009@gmail.com)

# Supplementary Figures and Tables

## Supplementary Figures

**Supplementary Figure 1.** The relative synonymous codon usage (RSCU) of the protein-coding genes in ten chloroplast genomes of seven *Dendroseris*, two *Sonchus* species, and *R. ligulata*. Amino acids are given on the x-axis, and the stacked RSCU values in each bar column are given on y-axis respectively. Each amino acid contains ten clustered bar columns representing ten species; 1^st^ column through 10^th^ column for *R. ligulata*, *Dendroseris litoralis*, *D. macrantha*, *D. marginata*, *D. pruinata*, *D. micrantha*, *D. berteroana*, *D. pinnata*, *Sonchus asper*, and *S. canariensis*.

**
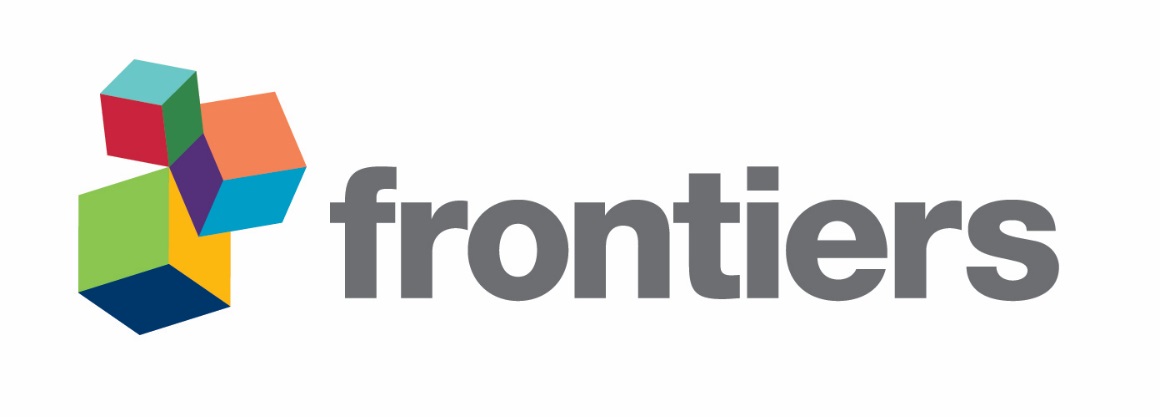

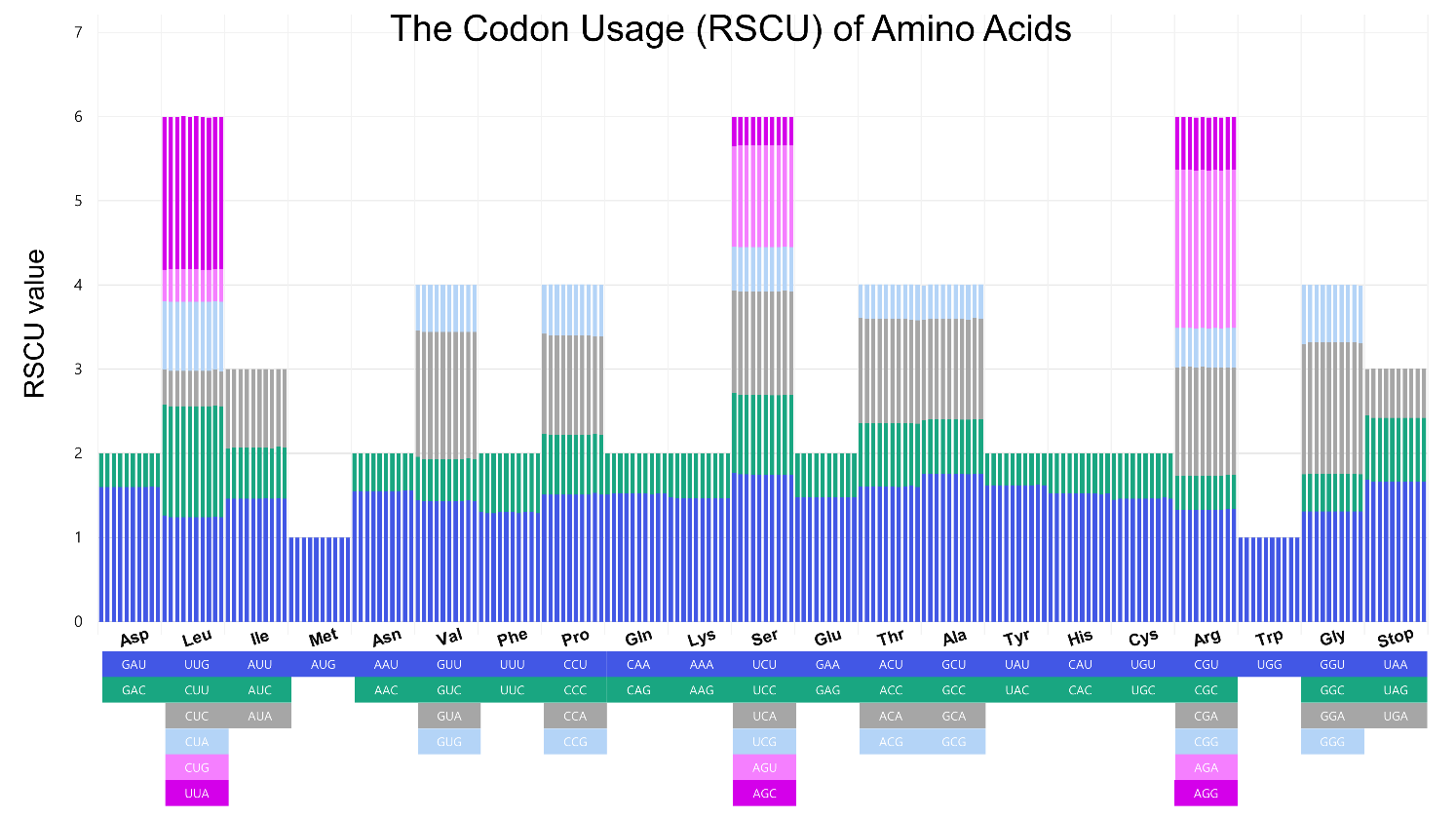
**
