# Supplementary material for: Plastid Phylogenomics of Dendroseris (Cichorieae; Asteraceae): Insights Into Structural Organization and Molecular Evolution of an Endemic Lineage From the Juan Fernández Islands
Source: Front Plant Sci. 2020 Nov 5;11:594272. doi: 10.3389/fpls.2020.594272 (PMC7674203; doi:10.3389/fpls.2020.594272)
Supplement: Supplementary file 1 [file Data_Sheet_1.zip › Table 2 - 2020-10-14T160846.290.DOCX]

Supplementary Material

Plastid phylogenomics of *Dendroseris* (Cichorieae; Asteraceae), endemic to the Juan Fernández Islands: Insights into structural organization and molecular evolution

**Myong-Suk Cho^1^, Seon-Hee Kim^1^, JiYoung Yang^2^, Daniel J. Crawford^3^, Tod F. Stuessy^4^, Patricio López-Sepúlveda^5^, and Seung-Chul Kim^1*^**

*** Correspondence**: Seung-Chul Kim: [sonchus96@skku.edu](mailto:sonchus96@skku.edu) or [sonchus2009@gmail.com](mailto:sonchus2009@gmail.com)

# Supplementary Figures and Tables

## Supplementary Figures

**Supplementary Figure 2.** Amino acid changes in predicted RNA editing sites in ten cp genomes of seven *Dendroseris*, two *Sonchus* species, *S. asper* and *S. canariensis,* and *Reichardia ligulata*. The scores (the proportion of sites that have the same amino acid at that position) of each edit site are stacked in each bar column. Color bricks indicate RNA editing effect: Alanine to Valine, A→V; Histidine to Tyrosine, H→Y; Leucine to Phenylalanine, L→F; Proline to Phenylalanine, P→F; Proline to Leucine, P→L; Proline to Serine, P→S; Arginine to Tryptophan, R→W; Serine to Phenylalanine, S→F; Serine to Leucine, S→L; Threonine to Isoleucine, T→I; Threonine to Methionine, T→M.


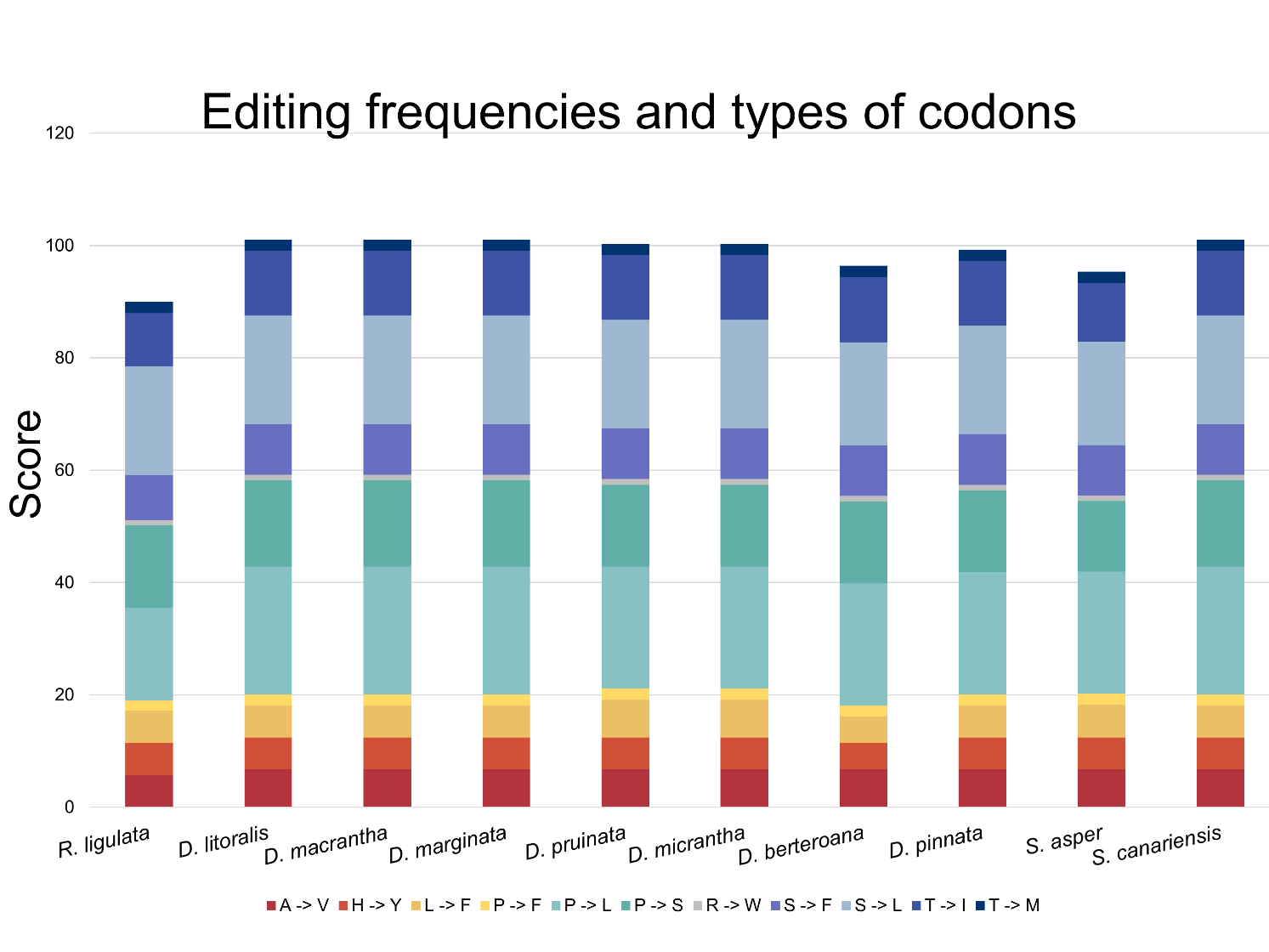


**
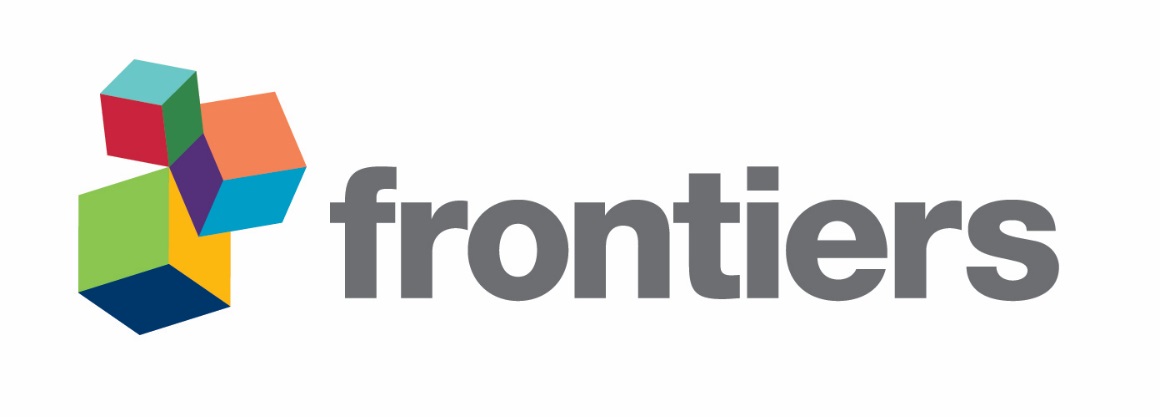
**
