# Supplementary material for: Plastid Phylogenomics of Dendroseris (Cichorieae; Asteraceae): Insights Into Structural Organization and Molecular Evolution of an Endemic Lineage From the Juan Fernández Islands
Source: Front Plant Sci. 2020 Nov 5;11:594272. doi: 10.3389/fpls.2020.594272 (PMC7674203; doi:10.3389/fpls.2020.594272)

Supplementary Material

Plastid phylogenomics of *Dendroseris* (Cichorieae; Asteraceae), endemic to the Juan Fernández Islands: Insights into structural organization and molecular evolution

**Myong-Suk Cho^1^, Seon-Hee Kim^1^, JiYoung Yang^2^, Daniel J. Crawford^3^, Tod F. Stuessy^4^, Patricio López-Sepúlveda^5^, and Seung-Chul Kim^1*^**

*** Correspondence**: Seung-Chul Kim: [sonchus96@skku.edu](mailto:sonchus96@skku.edu) or [sonchus2009@gmail.com](mailto:sonchus2009@gmail.com)

# Supplementary Figures and Tables

## Supplementary Figures

**
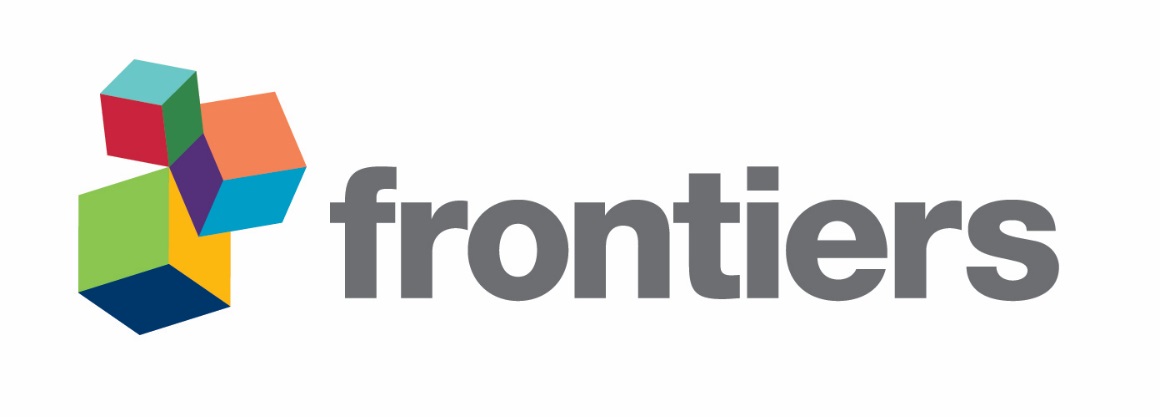
**

**Supplementary Figure 4.** Maximum likelihood tree of *Dendroseris* and closely related *Sonchus* based on the concatenated sequences of ten mutation hotspots regions identified in this study; *trn*S-*trn*C, *trn*C-*pet*N, *trn*T-*trn*L, *trn*L-*trn*F, *ndh*C-*trn*C, *psb*E-*pet*L, *ycf*1-*rps*15, *rpl*32-*ndh*F, *rpl*16 intron, and *ycf*1. Numbers above nodes are bootstrap values with 1000 replicates. Newly sequenced eight plastid genomes in this study are marked with an asterisk (*).


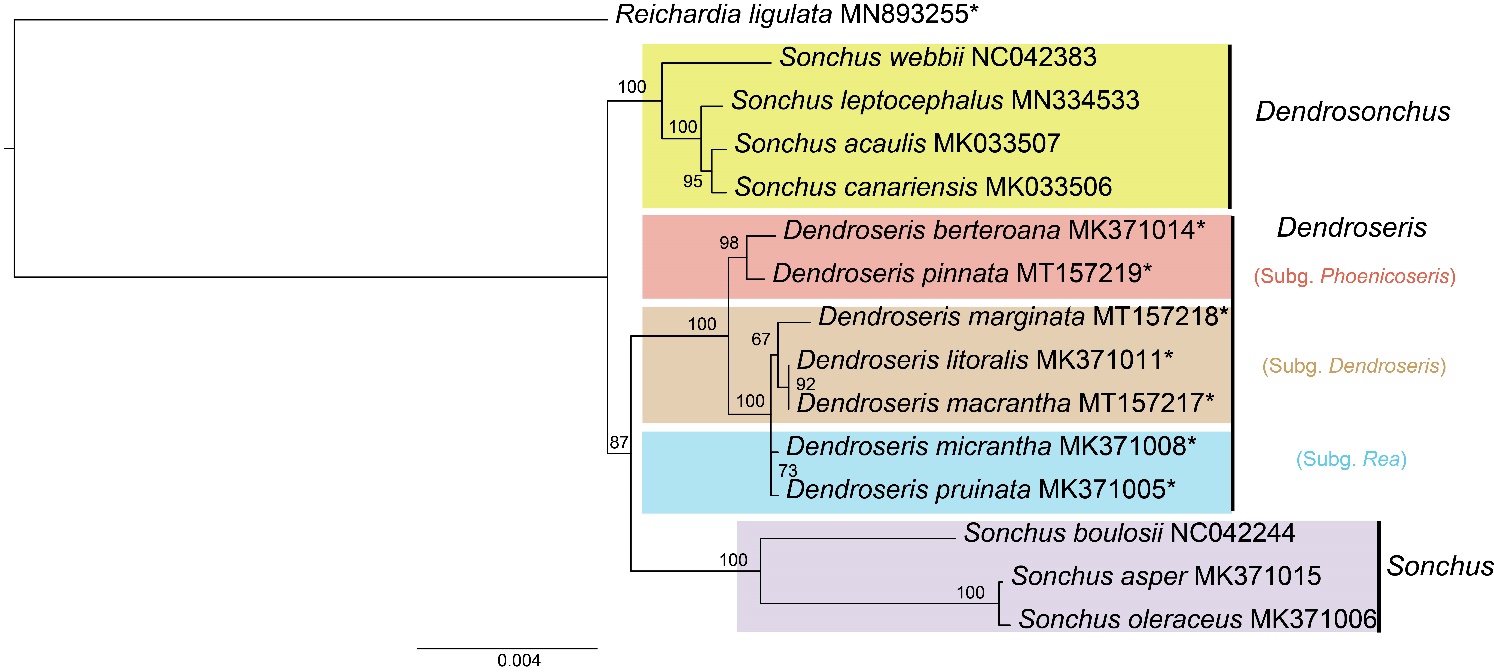

Supplement: Supplementary file 1 [file Data_Sheet_1.zip › Table 4 (97).DOCX]
