# Supplementary material for: Plastid Phylogenomics of Dendroseris (Cichorieae; Asteraceae): Insights Into Structural Organization and Molecular Evolution of an Endemic Lineage From the Juan Fernández Islands
Source: Front Plant Sci. 2020 Nov 5;11:594272. doi: 10.3389/fpls.2020.594272 (PMC7674203; doi:10.3389/fpls.2020.594272)
Supplement: Supplementary file 1 [file Data_Sheet_1.zip › Table 5 (62).DOCX]

Supplementary Material

Plastid phylogenomics of *Dendroseris* (Cichorieae; Asteraceae), endemic to the Juan Fernández Islands: Insights into structural organization and molecular evolution

**Myong-Suk Cho^1^, Seon-Hee Kim^1^, JiYoung Yang^2^, Daniel J. Crawford^3^, Tod F. Stuessy^4^, Patricio López-Sepúlveda^5^, and Seung-Chul Kim^1*^**

*** Correspondence**: Seung-Chul Kim: [sonchus96@skku.edu](mailto:sonchus96@skku.edu) or sonchus2009@gmail.com

# Supplementary Figures and Tables

## 1.2 Supplementary Tables

**
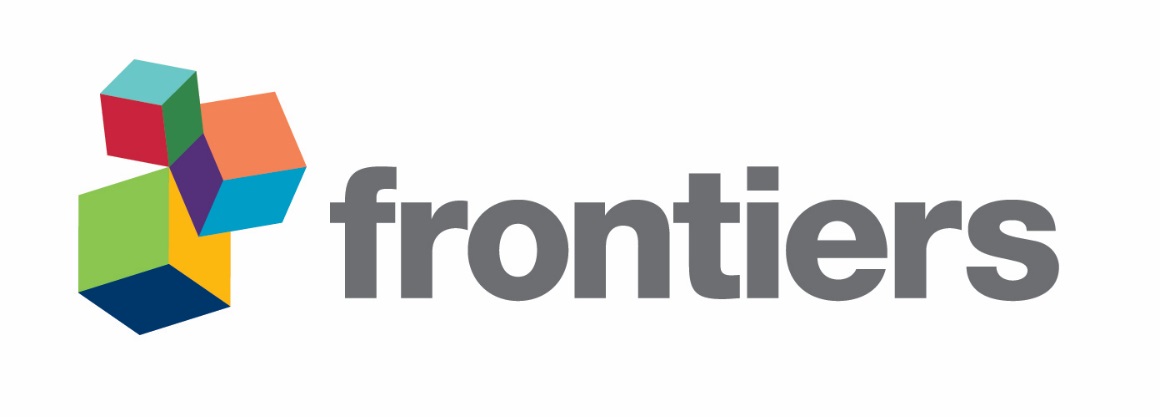
**

**Supplementary Table 1.** Codon-anticodon recognition pattern and codon usage for ten chloroplast genomes (seven *Dendroseris*, two *Sonchus* species, *S. asper* and *S. canariensis*, and *R. ligulata*). AA: Amino Acid (single letter amino acid code), RSCU: Relative synonymous codon usage value (the relative frequencies of occurrence of the synonymous codon for a specific amino acid)

|  | 1. ***Reichardia ligulata*** | | | **2. *Dendroseris litoralis*** | | | **3. *Dendroseris macrantha*** | | |
| --- | --- | --- | --- | --- | --- | --- | --- | --- | --- |
| AA | Codon | Count | RSCU | Codon | Count | RSCU | Codon | Count | RSCU |
| Asp (D) | GAU | 708 | 1.59 | GAU | 705 | 1.59 | GAU | 705 | 1.59 |
|  | GAC | 183 | 0.41 | GAC | 180 | 0.41 | GAC | 180 | 0.41 |
| Leu (L) | UUG | 510 | 1.25 | UUG | 501 | 1.24 | UUG | 501 | 1.24 |
|  | CUU | 529 | 1.3 | CUU | 526 | 1.3 | CUU | 526 | 1.3 |
|  | CUC | 154 | 0.38 | CUC | 153 | 0.38 | CUC | 153 | 0.38 |
|  | CUA | 326 | 0.8 | CUA | 322 | 0.8 | CUA | 322 | 0.8 |
|  | CUG | 146 | 0.36 | CUG | 151 | 0.37 | CUG | 151 | 0.37 |
|  | UUA | 779 | 1.91 | UUA | 768 | 1.9 | UUA | 768 | 1.9 |
| Ile (I) | AUU | 953 | 1.48 | AUU | 952 | 1.48 | AUU | 952 | 1.48 |
|  | AUC | 369 | 0.57 | AUC | 372 | 0.58 | AUC | 372 | 0.58 |
|  | AUA | 606 | 0.94 | AUA | 602 | 0.94 | AUA | 602 | 0.94 |
| Met (M) | AUG | 544 | 1 | AUG | 546 | 1 | AUG | 546 | 1 |
| Asn (N) | AAU | 848 | 1.57 | AAU | 849 | 1.57 | AAU | 849 | 1.57 |
|  | AAC | 234 | 0.43 | AAC | 234 | 0.43 | AAC | 234 | 0.43 |
| Val (V) | GUU | 449 | 1.45 | GUU | 447 | 1.45 | GUU | 447 | 1.45 |
|  | GUC | 155 | 0.5 | GUC | 152 | 0.49 | GUC | 152 | 0.49 |
|  | GUA | 476 | 1.53 | GUA | 471 | 1.53 | GUA | 471 | 1.53 |
|  | GUG | 161 | 0.52 | GUG | 165 | 0.53 | GUG | 165 | 0.53 |
| Phe (F) | UUU | 862 | 1.35 | UUU | 860 | 1.34 | UUU | 860 | 1.34 |
|  | UUC | 413 | 0.65 | UUC | 422 | 0.66 | UUC | 422 | 0.66 |
| Pro (P) | CCU | 377 | 1.55 | CCU | 374 | 1.55 | CCU | 374 | 1.55 |
|  | CCC | 170 | 0.7 | CCC | 165 | 0.69 | CCC | 165 | 0.69 |
|  | CCA | 283 | 1.16 | CCA | 277 | 1.15 | CCA | 277 | 1.15 |
|  | CCG | 142 | 0.58 | CCG | 147 | 0.61 | CCG | 147 | 0.61 |
| Gln (Q) | CAA | 628 | 1.52 | CAA | 625 | 1.53 | CAA | 625 | 1.53 |
|  | CAG | 198 | 0.48 | CAG | 193 | 0.47 | CAG | 193 | 0.47 |
| Lys (K) | AAA | 893 | 1.51 | AAA | 893 | 1.51 | AAA | 893 | 1.51 |
|  | AAG | 286 | 0.49 | AAG | 290 | 0.49 | AAG | 290 | 0.49 |
| Ser (S) | UCU | 507 | 1.79 | UCU | 499 | 1.76 | UCU | 499 | 1.76 |
|  | UCC | 256 | 0.9 | UCC | 258 | 0.91 | UCC | 258 | 0.91 |
|  | UCA | 341 | 1.2 | UCA | 345 | 1.22 | UCA | 345 | 1.22 |
|  | UCG | 144 | 0.51 | UCG | 145 | 0.51 | UCG | 145 | 0.51 |
|  | AGU | 354 | 1.25 | AGU | 358 | 1.26 | AGU | 358 | 1.26 |
|  | AGC | 99 | 0.35 | AGC | 94 | 0.33 | AGC | 94 | 0.33 |
| Glu (E) | GAA | 885 | 1.51 | GAA | 877 | 1.51 | GAA | 877 | 1.51 |
|  | GAG | 287 | 0.49 | GAG | 288 | 0.49 | GAG | 288 | 0.49 |
| Thr (T) | ACU | 466 | 1.62 | ACU | 466 | 1.62 | ACU | 466 | 1.62 |
|  | ACC | 219 | 0.76 | ACC | 218 | 0.76 | ACC | 218 | 0.76 |
|  | ACA | 360 | 1.25 | ACA | 356 | 1.24 | ACA | 356 | 1.24 |
|  | ACG | 108 | 0.37 | ACG | 112 | 0.39 | ACG | 112 | 0.39 |
| Ala (A) | GCU | 575 | 1.79 | GCU | 570 | 1.78 | GCU | 570 | 1.78 |
|  | GCC | 199 | 0.62 | GCC | 202 | 0.63 | GCC | 202 | 0.63 |
|  | GCA | 380 | 1.18 | GCA | 376 | 1.17 | GCA | 376 | 1.17 |
|  | GCG | 132 | 0.41 | GCG | 133 | 0.42 | GCG | 133 | 0.42 |
| Tyr (Y) | UAU | 693 | 1.62 | UAU | 700 | 1.63 | UAU | 700 | 1.63 |
|  | UAC | 162 | 0.38 | UAC | 161 | 0.37 | UAC | 161 | 0.37 |
| His (H) | CAU | 395 | 1.52 | CAU | 394 | 1.52 | CAU | 394 | 1.52 |
|  | CAC | 125 | 0.48 | CAC | 126 | 0.48 | CAC | 126 | 0.48 |
| Cys (C) | UGU | 181 | 1.47 | UGU | 181 | 1.48 | UGU | 181 | 1.48 |
|  | UGC | 65 | 0.53 | UGC | 63 | 0.52 | UGC | 63 | 0.52 |
| Arg (R) | CGU | 315 | 1.39 | CGU | 314 | 1.39 | CGU | 314 | 1.39 |
|  | CGC | 89 | 0.39 | CGC | 89 | 0.39 | CGC | 89 | 0.39 |
|  | CGA | 299 | 1.32 | CGA | 298 | 1.32 | CGA | 298 | 1.32 |
|  | CGG | 103 | 0.46 | CGG | 102 | 0.45 | CGG | 102 | 0.45 |
|  | AGA | 419 | 1.86 | AGA | 418 | 1.86 | AGA | 418 | 1.86 |
|  | AGG | 130 | 0.58 | AGG | 131 | 0.58 | AGG | 131 | 0.58 |
| Trp (W) | UGG | 397 | 1 | UGG | 400 | 1 | UGG | 400 | 1 |
| Gly (G) | GGU | 531 | 1.34 | GGU | 531 | 1.34 | GGU | 531 | 1.34 |
|  | GGC | 183 | 0.46 | GGC | 190 | 0.48 | GGC | 190 | 0.48 |
|  | GGA | 600 | 1.52 | GGA | 604 | 1.52 | GGA | 604 | 1.52 |
|  | GGG | 270 | 0.68 | GGG | 264 | 0.66 | GGG | 264 | 0.66 |
| Stop (*) | UAA | 45 | 1.69 | UAA | 44 | 1.65 | UAA | 44 | 1.65 |
|  | UAG | 20 | 0.75 | UAG | 20 | 0.75 | UAG | 20 | 0.75 |
|  | UGA | 15 | 0.56 | UGA | 16 | 0.6 | UGA | 16 | 0.6 |

|  | **4. *Dendroseris marginata*** | | | **5. *Dendroseris pruinata*** | | | **6. *Dendroseris micrantha*** | | |
| --- | --- | --- | --- | --- | --- | --- | --- | --- | --- |
| AA | Codon | Count | RSCU | Codon | Count | RSCU | Codon | Count | RSCU |
| Asp (D) | GAU | 705 | 1.59 | GAU | 705 | 1.59 | GAU | 705 | 1.59 |
|  | GAC | 180 | 0.41 | GAC | 181 | 0.41 | GAC | 180 | 0.41 |
| Leu (L) | UUG | 500 | 1.24 | UUG | 501 | 1.24 | UUG | 501 | 1.24 |
|  | CUU | 526 | 1.3 | CUU | 526 | 1.3 | CUU | 526 | 1.31 |
|  | CUC | 153 | 0.38 | CUC | 153 | 0.38 | CUC | 153 | 0.38 |
|  | CUA | 322 | 0.8 | CUA | 323 | 0.8 | CUA | 322 | 0.8 |
|  | CUG | 151 | 0.37 | CUG | 151 | 0.37 | CUG | 151 | 0.37 |
|  | UUA | 770 | 1.91 | UUA | 765 | 1.9 | UUA | 765 | 1.9 |
| Ile (I) | AUU | 952 | 1.48 | AUU | 952 | 1.48 | AUU | 952 | 1.48 |
|  | AUC | 372 | 0.58 | AUC | 372 | 0.58 | AUC | 372 | 0.58 |
|  | AUA | 602 | 0.94 | AUA | 602 | 0.94 | AUA | 602 | 0.94 |
| Met (M) | AUG | 546 | 1 | AUG | 546 | 1 | AUG | 546 | 1 |
| Asn (N) | AAU | 848 | 1.57 | AAU | 849 | 1.57 | AAU | 849 | 1.57 |
|  | AAC | 234 | 0.43 | AAC | 234 | 0.43 | AAC | 234 | 0.43 |
| Val (V) | GUU | 446 | 1.45 | GUU | 447 | 1.45 | GUU | 447 | 1.45 |
|  | GUC | 152 | 0.49 | GUC | 152 | 0.49 | GUC | 152 | 0.49 |
|  | GUA | 471 | 1.53 | GUA | 471 | 1.53 | GUA | 471 | 1.53 |
|  | GUG | 165 | 0.53 | GUG | 165 | 0.53 | GUG | 165 | 0.53 |
| Phe (F) | UUU | 861 | 1.34 | UUU | 863 | 1.35 | UUU | 865 | 1.35 |
|  | UUC | 420 | 0.66 | UUC | 420 | 0.65 | UUC | 419 | 0.65 |
| Pro (P) | CCU | 374 | 1.55 | CCU | 374 | 1.55 | CCU | 374 | 1.55 |
|  | CCC | 165 | 0.69 | CCC | 165 | 0.69 | CCC | 165 | 0.69 |
|  | CCA | 277 | 1.15 | CCA | 277 | 1.15 | CCA | 277 | 1.15 |
|  | CCG | 147 | 0.61 | CCG | 147 | 0.61 | CCG | 147 | 0.61 |
| Gln (Q) | CAA | 624 | 1.53 | CAA | 626 | 1.53 | CAA | 624 | 1.53 |
|  | CAG | 193 | 0.47 | CAG | 192 | 0.47 | CAG | 194 | 0.47 |
| Lys (K) | AAA | 891 | 1.51 | AAA | 892 | 1.51 | AAA | 893 | 1.51 |
|  | AAG | 290 | 0.49 | AAG | 290 | 0.49 | AAG | 290 | 0.49 |
| Ser (S) | UCU | 498 | 1.76 | UCU | 498 | 1.76 | UCU | 498 | 1.76 |
|  | UCC | 260 | 0.92 | UCC | 260 | 0.92 | UCC | 260 | 0.92 |
|  | UCA | 345 | 1.22 | UCA | 345 | 1.22 | UCA | 345 | 1.22 |
|  | UCG | 145 | 0.51 | UCG | 145 | 0.51 | UCG | 145 | 0.51 |
|  | AGU | 358 | 1.26 | AGU | 358 | 1.26 | AGU | 358 | 1.26 |
|  | AGC | 94 | 0.33 | AGC | 94 | 0.33 | AGC | 94 | 0.33 |
| Glu (E) | GAA | 878 | 1.51 | GAA | 878 | 1.51 | GAA | 880 | 1.51 |
|  | GAG | 288 | 0.49 | GAG | 287 | 0.49 | GAG | 287 | 0.49 |
| Thr (T) | ACU | 466 | 1.62 | ACU | 466 | 1.62 | ACU | 466 | 1.62 |
|  | ACC | 218 | 0.76 | ACC | 218 | 0.76 | ACC | 218 | 0.76 |
|  | ACA | 356 | 1.24 | ACA | 356 | 1.24 | ACA | 356 | 1.24 |
|  | ACG | 112 | 0.39 | ACG | 112 | 0.39 | ACG | 112 | 0.39 |
| Ala (A) | GCU | 570 | 1.78 | GCU | 570 | 1.78 | GCU | 570 | 1.78 |
|  | GCC | 202 | 0.63 | GCC | 202 | 0.63 | GCC | 202 | 0.63 |
|  | GCA | 376 | 1.17 | GCA | 376 | 1.17 | GCA | 375 | 1.17 |
|  | GCG | 133 | 0.42 | GCG | 133 | 0.42 | GCG | 133 | 0.42 |
| Tyr (Y) | UAU | 700 | 1.63 | UAU | 700 | 1.63 | UAU | 700 | 1.63 |
|  | UAC | 161 | 0.37 | UAC | 161 | 0.37 | UAC | 161 | 0.37 |
| His (H) | CAU | 394 | 1.52 | CAU | 394 | 1.52 | CAU | 394 | 1.52 |
|  | CAC | 126 | 0.48 | CAC | 126 | 0.48 | CAC | 126 | 0.48 |
| Cys (C) | UGU | 181 | 1.48 | UGU | 181 | 1.48 | UGU | 181 | 1.48 |
|  | UGC | 63 | 0.52 | UGC | 63 | 0.52 | UGC | 63 | 0.52 |
| Arg (R) | CGU | 314 | 1.39 | CGU | 314 | 1.39 | CGU | 314 | 1.39 |
|  | CGC | 89 | 0.39 | CGC | 89 | 0.39 | CGC | 89 | 0.4 |
|  | CGA | 297 | 1.32 | CGA | 298 | 1.32 | CGA | 298 | 1.32 |
|  | CGG | 103 | 0.46 | CGG | 102 | 0.45 | CGG | 101 | 0.45 |
|  | AGA | 418 | 1.85 | AGA | 418 | 1.86 | AGA | 418 | 1.86 |
|  | AGG | 132 | 0.59 | AGG | 131 | 0.58 | AGG | 131 | 0.58 |
| Trp (W) | UGG | 400 | 1 | UGG | 400 | 1 | UGG | 401 | 1 |
| Gly (G) | GGU | 531 | 1.34 | GGU | 531 | 1.34 | GGU | 530 | 1.34 |
|  | GGC | 190 | 0.48 | GGC | 190 | 0.48 | GGC | 190 | 0.48 |
|  | GGA | 604 | 1.52 | GGA | 604 | 1.52 | GGA | 604 | 1.52 |
|  | GGG | 264 | 0.66 | GGG | 264 | 0.66 | GGG | 264 | 0.66 |
| Stop (*) | UAA | 44 | 1.65 | UAA | 44 | 1.65 | UAA | 44 | 1.65 |
|  | UAG | 20 | 0.75 | UAG | 20 | 0.75 | UAG | 20 | 0.75 |
|  | UGA | 16 | 0.6 | UGA | 16 | 0.6 | UGA | 16 | 0.6 |

|  | **7. *Dendroseris berteroana*** | | | **8. *Dendroseris pinnata*** | | | **9. *Sonchus asper*** | | | **10. *Sonchus canariensis*** | | |
| --- | --- | --- | --- | --- | --- | --- | --- | --- | --- | --- | --- | --- |
| AA | Codon | Count | RSCU | Codon | Count | RSCU | Codon | Count | RSCU | Codon | Count | RSCU |
| Asp (D) | GAU | 706 | 1.59 | GAU | 705 | 1.59 | GAU | 708 | 1.6 | GAU | 708 | 1.59 |
|  | GAC | 182 | 0.41 | GAC | 181 | 0.41 | GAC | 177 | 0.4 | GAC | 181 | 0.41 |
| Leu (L) | UUG | 499 | 1.24 | UUG | 499 | 1.24 | UUG | 506 | 1.25 | UUG | 501 | 1.24 |
|  | CUU | 527 | 1.31 | CUU | 527 | 1.31 | CUU | 528 | 1.31 | CUU | 525 | 1.3 |
|  | CUC | 154 | 0.38 | CUC | 154 | 0.38 | CUC | 153 | 0.38 | CUC | 151 | 0.37 |
|  | CUA | 323 | 0.8 | CUA | 323 | 0.8 | CUA | 323 | 0.8 | CUA | 324 | 0.8 |
|  | CUG | 149 | 0.37 | CUG | 149 | 0.37 | CUG | 148 | 0.37 | CUG | 151 | 0.37 |
|  | UUA | 770 | 1.91 | UUA | 769 | 1.91 | UUA | 768 | 1.9 | UUA | 767 | 1.9 |
| Ile (I) | AUU | 954 | 1.49 | AUU | 953 | 1.48 | AUU | 949 | 1.49 | AUU | 952 | 1.48 |
|  | AUC | 370 | 0.58 | AUC | 371 | 0.58 | AUC | 372 | 0.58 | AUC | 373 | 0.58 |
|  | AUA | 602 | 0.94 | AUA | 602 | 0.94 | AUA | 592 | 0.93 | AUA | 601 | 0.94 |
| Met (M) | AUG | 547 | 1 | AUG | 546 | 1 | AUG | 549 | 1 | AUG | 545 | 1 |
| Asn (N) | AAU | 850 | 1.57 | AAU | 850 | 1.57 | AAU | 852 | 1.57 | AAU | 850 | 1.57 |
|  | AAC | 234 | 0.43 | AAC | 234 | 0.43 | AAC | 230 | 0.43 | AAC | 231 | 0.43 |
| Val (V) | GUU | 446 | 1.45 | GUU | 448 | 1.45 | GUU | 451 | 1.46 | GUU | 447 | 1.45 |
|  | GUC | 151 | 0.49 | GUC | 151 | 0.49 | GUC | 151 | 0.49 | GUC | 151 | 0.49 |
|  | GUA | 472 | 1.53 | GUA | 472 | 1.53 | GUA | 472 | 1.52 | GUA | 470 | 1.52 |
|  | GUG | 164 | 0.53 | GUG | 165 | 0.53 | GUG | 165 | 0.53 | GUG | 166 | 0.54 |
| Phe (F) | UUU | 861 | 1.34 | UUU | 863 | 1.35 | UUU | 861 | 1.35 | UUU | 862 | 1.34 |
|  | UUC | 421 | 0.66 | UUC | 419 | 0.65 | UUC | 418 | 0.65 | UUC | 422 | 0.66 |
| Pro (P) | CCU | 373 | 1.55 | CCU | 374 | 1.55 | CCU | 377 | 1.57 | CCU | 376 | 1.56 |
|  | CCC | 165 | 0.69 | CCC | 165 | 0.69 | CCC | 162 | 0.68 | CCC | 166 | 0.69 |
|  | CCA | 277 | 1.15 | CCA | 277 | 1.15 | CCA | 273 | 1.14 | CCA | 278 | 1.15 |
|  | CCG | 147 | 0.61 | CCG | 147 | 0.61 | CCG | 148 | 0.62 | CCG | 147 | 0.61 |
| Gln (Q) | CAA | 625 | 1.53 | CAA | 624 | 1.53 | CAA | 627 | 1.53 | CAA | 627 | 1.53 |
|  | CAG | 193 | 0.47 | CAG | 194 | 0.47 | CAG | 192 | 0.47 | CAG | 193 | 0.47 |
| Lys (K) | AAA | 892 | 1.51 | AAA | 891 | 1.51 | AAA | 892 | 1.51 | AAA | 890 | 1.51 |
|  | AAG | 292 | 0.49 | AAG | 292 | 0.49 | AAG | 290 | 0.49 | AAG | 291 | 0.49 |
| Ser (S) | UCU | 499 | 1.76 | UCU | 499 | 1.76 | UCU | 498 | 1.76 | UCU | 498 | 1.76 |
|  | UCC | 257 | 0.91 | UCC | 258 | 0.91 | UCC | 260 | 0.92 | UCC | 261 | 0.92 |
|  | UCA | 348 | 1.23 | UCA | 347 | 1.22 | UCA | 347 | 1.22 | UCA | 344 | 1.21 |
|  | UCG | 145 | 0.51 | UCG | 145 | 0.51 | UCG | 144 | 0.51 | UCG | 145 | 0.51 |
|  | AGU | 357 | 1.26 | AGU | 358 | 1.26 | AGU | 357 | 1.26 | AGU | 358 | 1.26 |
|  | AGC | 95 | 0.34 | AGC | 95 | 0.33 | AGC | 96 | 0.34 | AGC | 94 | 0.33 |
| Glu (E) | GAA | 877 | 1.51 | GAA | 877 | 1.51 | GAA | 883 | 1.51 | GAA | 880 | 1.51 |
|  | GAG | 288 | 0.49 | GAG | 288 | 0.49 | GAG | 283 | 0.49 | GAG | 287 | 0.49 |
| Thr (T) | ACU | 467 | 1.62 | ACU | 467 | 1.62 | ACU | 469 | 1.63 | ACU | 466 | 1.62 |
|  | ACC | 220 | 0.76 | ACC | 218 | 0.76 | ACC | 216 | 0.75 | ACC | 218 | 0.76 |
|  | ACA | 358 | 1.24 | ACA | 357 | 1.24 | ACA | 354 | 1.23 | ACA | 355 | 1.23 |
|  | ACG | 111 | 0.38 | ACG | 112 | 0.39 | ACG | 114 | 0.4 | ACG | 114 | 0.4 |
| Ala (A) | GCU | 568 | 1.78 | GCU | 569 | 1.78 | GCU | 570 | 1.78 | GCU | 570 | 1.78 |
|  | GCC | 202 | 0.63 | GCC | 203 | 0.63 | GCC | 202 | 0.63 | GCC | 202 | 0.63 |
|  | GCA | 377 | 1.18 | GCA | 376 | 1.17 | GCA | 381 | 1.19 | GCA | 375 | 1.17 |
|  | GCG | 132 | 0.41 | GCG | 133 | 0.42 | GCG | 131 | 0.41 | GCG | 134 | 0.42 |
| Tyr (Y) | UAU | 700 | 1.62 | UAU | 699 | 1.62 | UAU | 700 | 1.63 | UAU | 696 | 1.62 |
|  | UAC | 162 | 0.38 | UAC | 162 | 0.38 | UAC | 158 | 0.37 | UAC | 162 | 0.38 |
| His (H) | CAU | 396 | 1.52 | CAU | 395 | 1.52 | CAU | 393 | 1.51 | CAU | 395 | 1.52 |
|  | CAC | 124 | 0.48 | CAC | 125 | 0.48 | CAC | 127 | 0.49 | CAC | 126 | 0.48 |
| Cys (C) | UGU | 181 | 1.49 | UGU | 181 | 1.48 | UGU | 183 | 1.5 | UGU | 180 | 1.48 |
|  | UGC | 62 | 0.51 | UGC | 63 | 0.52 | UGC | 61 | 0.5 | UGC | 63 | 0.52 |
| Arg (R) | CGU | 313 | 1.39 | CGU | 314 | 1.39 | CGU | 315 | 1.4 | CGU | 316 | 1.4 |
|  | CGC | 89 | 0.39 | CGC | 89 | 0.39 | CGC | 89 | 0.4 | CGC | 89 | 0.39 |
|  | CGA | 297 | 1.32 | CGA | 297 | 1.32 | CGA | 293 | 1.3 | CGA | 293 | 1.3 |
|  | CGG | 104 | 0.46 | CGG | 103 | 0.46 | CGG | 105 | 0.47 | CGG | 105 | 0.47 |
|  | AGA | 418 | 1.85 | AGA | 419 | 1.86 | AGA | 418 | 1.86 | AGA | 418 | 1.85 |
|  | AGG | 132 | 0.59 | AGG | 131 | 0.58 | AGG | 131 | 0.58 | AGG | 132 | 0.59 |
| Trp (W) | UGG | 400 | 1 | UGG | 400 | 1 | UGG | 400 | 1 | UGG | 400 | 1 |
| Gly (G) | GGU | 532 | 1.34 | GGU | 531 | 1.34 | GGU | 533 | 1.34 | GGU | 531 | 1.34 |
|  | GGC | 189 | 0.48 | GGC | 189 | 0.48 | GGC | 189 | 0.48 | GGC | 187 | 0.47 |
|  | GGA | 604 | 1.52 | GGA | 605 | 1.52 | GGA | 603 | 1.52 | GGA | 605 | 1.52 |
|  | GGG | 264 | 0.66 | GGG | 264 | 0.66 | GGG | 264 | 0.66 | GGG | 264 | 0.67 |
| Stop (*) | UAA | 44 | 1.65 | UAA | 44 | 1.65 | UAA | 44 | 1.65 | UAA | 44 | 1.65 |
|  | UAG | 20 | 0.75 | UAG | 20 | 0.75 | UAG | 20 | 0.75 | UAG | 20 | 0.75 |
|  | UGA | 16 | 0.6 | UGA | 16 | 0.6 | UGA | 16 | 0.6 | UGA | 16 | 0.6 |
