# Supplementary material for: Plastid Phylogenomics of Dendroseris (Cichorieae; Asteraceae): Insights Into Structural Organization and Molecular Evolution of an Endemic Lineage From the Juan Fernández Islands
Source: Front Plant Sci. 2020 Nov 5;11:594272. doi: 10.3389/fpls.2020.594272 (PMC7674203; doi:10.3389/fpls.2020.594272)
Supplement: Supplementary file 1 [file Data_Sheet_1.zip › Table 6 (27).DOCX]

Supplementary Material

Plastid phylogenomics of *Dendroseris* (Cichorieae; Asteraceae), endemic to the Juan Fernández Islands: Insights into structural organization and molecular evolution

**Myong-Suk Cho^1^, Seon-Hee Kim^1^, JiYoung Yang^2^, Daniel J. Crawford^3^, Tod F. Stuessy^4^, Patricio López-Sepúlveda^5^, and Seung-Chul Kim^1*^**

*** Correspondence**: Seung-Chul Kim: [sonchus96@skku.edu](mailto:sonchus96@skku.edu) or sonchus2009@gmail.com

# Supplementary Figures and Tables

## 1.2 Supplementary Tables

**Supplementary Table 2.** Predicted RNA editing sites in the ten chloroplast genomes (seven *Dendroseris*, two *Sonchus* species, *S. asper* and *S. canariensis*, and *R. ligulata*).

| **1. *Reichardia ligulata*** | | | | | **2. *Dendroseris itoralis*** | | | |
| --- | --- | --- | --- | --- | --- | --- | --- | --- |
| Gene | Nucleotide Position | Amino Acid Position | Codon Conversion | Score | Nucleotide Position | Amino Acid Position | Codon Conversion | Score |
| *acc*D | 370 | 124 | CCT (P) => TCT (S) | 1 | 370 | 124 | CCT (P) => TCT (S) | 1 |
|  | 451 | 151 | CAC (H) => TAC (Y) | 1 | 451 | 151 | CAC (H) => TAC (Y) | 1 |
|  | 1225 | 409 | CCA (P) => TCA (S) | 1 | 1231 | 411 | CCA (P) => TCA (S) | 1 |
|  | 1433 | 478 | CCT (P) => CTT (L) | 1 | 1439 | 480 | CCT (P) => CTT (L) | 1 |
| *atp*A | 773 | 258 | TCA (S) => TTA (L) | 1 | 773 | 258 | TCA (S) => TTA (L) | 1 |
|  |  |  |  |  | 791 | 264 | CCC (P) => CTC (L) | 1 |
| *atp*B | 322 | 108 | CTT (L) => TTT (F) | 1 | 322 | 108 | CTT (L) => TTT (F) | 1 |
|  | 434 | 145 | TCC (S) => TTC (F) | 1 | 434 | 145 | TCC (S) => TTC (F) | 1 |
|  | 542 | 181 | CCC (P) => CTC (L) | 1 | 542 | 181 | CCC (P) => CTC (L) | 1 |
|  | 839 | 280 | TCC (S) => TTC (F) | 1 | 839 | 280 | TCC (S) => TTC (F) | 1 |
|  | 899 | 300 | GCC (A) => GTC (V) | 1 | 899 | 300 | GCC (A) => GTC (V) | 1 |
|  | 1247 | 416 | ACC (T) => ATC (I) | 1 | 1247 | 416 | ACC (T) => ATC (I) | 1 |
| *atp*F | 0 |  |  |  | 0 |  |  |  |
| *atp*I | 0 |  |  |  | 629 | 210 | TCA (S) => TTA (L) | 1 |
| *ccs*A | 26 | 9 | ACC (T) => ATC (I) | 1 | 26 | 9 | ACC (T) => ATC (I) | 1 |
|  | 40 | 14 | CCC (P) => TCC (S) | 1 | 40 | 14 | CCC (P) => TCC (S) | 1 |
|  | 205 | 69 | CCA (P) => TCA (S) | 1 | 173 | 58 | CCA (P) => CTA (L) | 1 |
|  |  |  |  |  | 205 | 69 | CCA (P) => TCA (S) | 1 |
| *clp*P | 146 | 49 | CCC (P) => CTC (L) | 1 | 146 | 49 | CCC (P) => CTC (L) | 1 |
|  | 164 | 55 | ACA (T) => ATA (I) | 0.86 | 164 | 55 | ACA (T) => ATA (I) | 0.86 |
|  | 191 | 64 | TCA (S) => TTA (L) | 0.86 | 191 | 64 | TCA (S) => TTA (L) | 0.86 |
|  | 455 | 152 | CCA (P) => CTA (L) | 0.86 | 323 | 108 | TCT (S) => TTT (F) | 1 |
|  | 542 | 181 | TCC (S) => TTC (F) | 1 | 329 | 110 | CCT (P) => CTT (L) | 1 |
|  |  |  |  |  | 542 | 181 | TCC (S) => TTC (F) | 1 |
| *mat*K | 68 | 23 | CCT (P) => CTT (L) | 1 | 68 | 23 | CCT (P) => CTT (L) | 1 |
|  | 365 | 122 | CCC (P) => CTC (L) | 1 | 365 | 122 | CCC (P) => CTC (L) | 1 |
|  | 482 | 161 | ACT (T) => ATT (I) | 1 | 482 | 161 | ACT (T) => ATT (I) | 1 |
|  | 652 | 218 | CCC (P) => TTC (F) | 0.86 | 568 | 190 | CTT (L) => TTT (F) | 0.86 |
|  | 653 | 218 | CCC (P) => TTC (F) | 0.86 | 718 | 240 | CCA (P) => TCA (S) | 1 |
|  | 718 | 240 | CCA (P) => TCA (S) | 1 | 739 | 247 | CCT (P) => TCT (S) | 1 |
|  | 739 | 247 | CCT (P) => TCT (S) | 1 | 1168 | 390 | CCC (P) => TTC (F) | 1 |
|  |  |  |  |  | 1169 | 390 | CCC (P) => TTC (F) | 1 |
| *ndh*A | 566 | 189 | TCA (S) => TTA (L) | 1 | 107 | 36 | CCT (P) => CTT (L) | 1 |
|  | 1073 | 358 | TCC (S) => TTC (F) | 1 | 566 | 189 | TCA (S) => TTA (L) | 1 |
|  |  |  |  |  | 1073 | 358 | TCC (S) => TTC (F) | 1 |
| *ndh*B | 149 | 50 | TCA (S) => TTA (L) | 1 | 149 | 50 | TCA (S) => TTA (L) | 1 |
|  | 467 | 156 | CCA (P) => CTA (L) | 1 | 467 | 156 | CCA (P) => CTA (L) | 1 |
|  | 586 | 196 | CAT (H) => TAT (Y) | 1 | 586 | 196 | CAT (H) => TAT (Y) | 1 |
|  | 611 | 204 | TCA (S) => TTA (L) | 0.8 | 611 | 204 | TCA (S) => TTA (L) | 0.8 |
|  | 737 | 246 | CCA (P) => CTA (L) | 1 | 737 | 246 | CCA (P) => CTA (L) | 1 |
|  | 746 | 249 | TCT (S) => TTT (F) | 1 | 746 | 249 | TCT (S) => TTT (F) | 1 |
|  | 830 | 277 | TCA (S) => TTA (L) | 1 | 830 | 277 | TCA (S) => TTA (L) | 1 |
|  | 836 | 279 | TCA (S) => TTA (L) | 1 | 836 | 279 | TCA (S) => TTA (L) | 1 |
|  | 1481 | 494 | CCA (P) => CTA (L) | 1 | 1481 | 494 | CCA (P) => CTA (L) | 1 |
|  |  |  |  |  |  |  |  |  |
| *ndh*D | 263 | 88 | TCA (S) => TTA (L) | 1 | 263 | 88 | TCA (S) => TTA (L) | 1 |
|  | 758 | 253 | TCA (S) => TTA (L) | 1 | 479 | 160 | TCA (S) => TTA (L) | 1 |
|  | 767 | 256 | CCC (P) => CTC (L) | 1 | 758 | 253 | TCA (S) => TTA (L) | 1 |
|  | 1178 | 393 | TCA (S) => TTA (L) | 0.8 | 767 | 256 | CCC (P) => CTC (L) | 1 |
|  |  |  |  |  | 1178 | 393 | TCA (S) => TTA (L) | 0.8 |
| *ndh*F | 290 | 97 | TCA (S) => TTA (L) | 1 | 290 | 97 | TCA (S) => TTA (L) | 1 |
| *ndh*G | 166 | 56 | CAT (H) => TAT (Y) | 0.8 | 166 | 56 | CAT (H) => TAT (Y) | 0.8 |
|  | 314 | 105 | ACA (T) => ATA (I) | 0.8 | 314 | 105 | ACA (T) => ATA (I) | 0.8 |
| *pet*B | 418 | 140 | CGG (R) => TGG (W) | 1 | 418 | 140 | CGG (R) => TGG (W) | 1 |
|  | 611 | 204 | CCA (P) => CTA (L) | 1 | 611 | 204 | CCA (P) => CTA (L) | 1 |
| *pet*D | 0 |  |  |  | 0 |  |  |  |
| *pet*G | 0 |  |  |  | 0 |  |  |  |
| *pet*L | 0 |  |  |  | 0 |  |  |  |
| *psa*B | 349 | 117 | CCA (P) => TCA (S) | 1 | 349 | 117 | CCA (P) => TCA (S) | 1 |
|  | 424 | 142 | CCA (P) => TCA (S) | 1 | 424 | 142 | CCA (P) => TCA (S) | 1 |
|  | 737 | 246 | TCG (S) => TTG (L) | 1 | 788 | 263 | CCC (P) => CTC (L) | 1 |
|  | 821 | 274 | CCA (P) => CTA (L) | 0.86 | 1202 | 401 | GCC (A) => GTC (V) | 1 |
|  | 935 | 312 | CCA (P) => CTA (L) | 1 | 1268 | 423 | CCC (P) => CTC (L) | 1 |
|  | 977 | 326 | TCG (S) => TTG (L) | 1 | 1307 | 436 | ACT (T) => ATT (I) | 1 |
|  | 1246 | 416 | CTT (L) => TTT (F) | 1 | 1342 | 448 | CCC (P) => TCC (S) | 1 |
|  | 1307 | 436 | ACT (T) => ATT (I) | 1 | 1487 | 496 | ACT (T) => ATT (I) | 1 |
|  | 1342 | 448 | CCC (P) => TCC (S) | 1 | 1889 | 630 | TCG (S) => TTG (L) | 1 |
|  | 1487 | 496 | ACT (T) => ATT (I) | 1 | 1981 | 661 | CTC (L) => TTC (F) | 1 |
|  | 1889 | 630 | TCG (S) => TTG (L) | 1 |  |  |  |  |
|  | 1981 | 661 | CTC (L) => TTC (F) | 1 |  |  |  |  |
| *psa*I | 0 |  |  |  | 0 |  |  |  |
| *psb*B | 0 |  |  |  | 0 |  |  |  |
| *psb*E | 101 | 34 | CCG (P) => CTG (L) | 1 | 101 | 34 | CCG (P) => CTG (L) | 1 |
|  | 107 | 36 | GCT (A) => GTT (V) | 1 | 107 | 36 | GCT (A) => GTT (V) | 1 |
|  | 200 | 67 | TCG (S) => TTG (L) | 1 |  |  |  |  |
| *psb*F | 0 |  |  |  | 0 |  |  |  |
| *psb*L | 58 | 20 | CCA (P) => TCA (S) | 1 | 58 | 20 | CCA (P) => TCA (S) | 1 |
| *rpl*2 | 407 | 136 | ACG (T) => ATG (M) | 1 | 407 | 136 | ACG (T) => ATG (M) | 1 |
|  | 446 | 149 | ACT (T) => ATT (I) | 1 | 446 | 149 | ACT (T) => ATT (I) | 1 |
|  | 659 | 220 | GCA (A) => GTA (V) | 0.86 | 659 | 220 | GCA (A) => GTA (V) | 0.86 |
|  | 677 | 226 | ACG (T) => ATG (M) | 1 | 677 | 226 | ACG (T) => ATG (M) | 1 |
| *rpl*20 | 163 | 55 | CCC (P) => TCC (S) | 1 | 163 | 55 | CCC (P) => TCC (S) | 1 |
|  | 284 | 95 | TCG (S) => TTG (L) | 1 | 284 | 95 | TCG (S) => TTG (L) | 1 |
|  | 305 | 102 | CCC (P) => CTC (L) | 0.86 | 305 | 102 | CCC (P) => CTC (L) | 0.86 |
|  | 364 | 122 | CCG (P) => TCG (S) | 0.86 | 364 | 122 | CCG (P) => TCG (S) | 0.86 |
| *rpl*23 | 58 | 20 | CAT (H) => TAT (Y) | 1 | 58 | 20 | CAT (H) => TAT (Y) | 1 |
|  | 230 | 77 | CCC (P) => CTC (L) | 1 | 230 | 77 | CCC (P) => CTC (L) | 1 |
| *rpo*A | 88 | 30 | CAT (H) => TAT (Y) | 0.86 | 88 | 30 | CAT (H) => TAT (Y) | 0.86 |
|  | 694 | 232 | CTC (L) => TTC (F) | 0.86 | 694 | 232 | CTC (L) => TTC (F) | 0.86 |
|  | 704 | 235 | TCC (S) => TTC (F) | 1 | 704 | 235 | TCC (S) => TTC (F) | 1 |
|  | 860 | 287 | TCG (S) => TTG (L) | 1 | 860 | 287 | TCG (S) => TTG (L) | 1 |
|  | 934 | 312 | CTT (L) => TTT (F) | 1 | 934 | 312 | CTT (L) => TTT (F) | 1 |
|  | 959 | 320 | ACA (T) => ATA (I) | 0.86 | 959 | 320 | ACA (T) => ATA (I) | 0.86 |
| *rpo*B | 983 | 328 | GCT (A) => GTT (V) | 1 | 983 | 328 | GCT (A) => GTT (V) | 1 |
| *rpo*C1 | 508 | 170 | CCC (P) => TCC (S) | 1 | 508 | 170 | CCC (P) => TCC (S) | 1 |
|  | 1589 | 530 | GCA (A) => GTA (V) | 0.86 | 1589 | 530 | GCA (A) => GTA (V) | 0.86 |
|  | 1747 | 583 | CCC (P) => TCC (S) | 0.86 | 1747 | 583 | CCC (P) => TCC (S) | 0.86 |
|  | 2042 | 681 | CCA (P) => CTA (L) | 1 | 2042 | 681 | CCA (P) => CTA (L) | 1 |
| *rpo*C2 | 2084 | 695 | GCA (A) => GTA (V) | 1 | 2084 | 695 | GCA (A) => GTA (V) | 1 |
|  | 3722 | 1241 | TCG (S) => TTG (L) | 0.86 | 3722 | 1241 | TCG (S) => TTG (L) | 0.86 |
| *rps*2 | 0 |  |  |  | 248 | 83 | TCA (S) => TTA (L) | 1 |
| *rps*8 | 94 | 32 | CCC (P) => TCC (S) | 0.86 | 94 | 32 | CCC (P) => TCC (S) | 0.86 |
|  | 332 | 111 | TCG (S) => TTG (L) | 1 | 104 | 35 | ACC (T) => ATC (I) | 1 |
|  | 403 | 135 | CAT (H) => TAT (Y) | 1 | 200 | 67 | CCT (P) => CTT (L) | 1 |
|  |  |  |  |  | 206 | 69 | CCG (P) => CTG (L) | 0.86 |
|  |  |  |  |  | 278 | 93 | ACC (T) => ATC (I) | 1 |
|  |  |  |  |  | 332 | 111 | TCG (S) => TTG (L) | 1 |
|  |  |  |  |  | 403 | 135 | CAT (H) => TAT (Y) | 1 |
| *rps*14 | 230 | 77 | TCG (S) => TTG (L) | 1 | 230 | 77 | TCG (S) => TTG (L) | 1 |
| *rps*16 | 0 |  |  |  | 82 | 28 | CCT (P) => TCT (S) | 0.8 |
|  |  |  |  |  | 104 | 35 | CCT (P) => CTT (L) | 1 |
| *ycf*3 | 31 | 11 | CTC (L) => TTC (F) | 1 | 31 | 11 | CTC (L) => TTC (F) | 1 |
|  | 110 | 37 | TCC (S) => TTC (F) | 1 | 110 | 37 | TCC (S) => TTC (F) | 1 |
|  | 350 | 117 | ACA (T) => ATA (I) | 1 | 350 | 117 | ACA (T) => ATA (I) | 1 |
|  | 503 | 168 | TCC (S) => TTC (F) | 1 | 503 | 168 | TCC (S) => TTC (F) | 1 |
|  |  |  |  |  |  |  |  |  |
| Total | 93 sites |  |  |  | 104 sites |  |  |  |

| **3. *Dendroseris macrantha*** | | | | | **4. *Dendroseris marginata*** | | | |
| --- | --- | --- | --- | --- | --- | --- | --- | --- |
| Gene | Nucleotide Position | Amino Acid Position | Codon Conversion | Score | Nucleotide Position | Amino Acid Position | Codon Conversion | Score |
| *acc*D | 370 | 124 | CCT (P) => TCT (S) | 1 | 370 | 124 | CCT (P) => TCT (S) | 1 |
|  | 451 | 151 | CAC (H) => TAC (Y) | 1 | 451 | 151 | CAC (H) => TAC (Y) | 1 |
|  | 1231 | 411 | CCA (P) => TCA (S) | 1 | 1231 | 411 | CCA (P) => TCA (S) | 1 |
|  | 1439 | 480 | CCT (P) => CTT (L) | 1 | 1439 | 480 | CCT (P) => CTT (L) | 1 |
| *atp*A | 773 | 258 | TCA (S) => TTA (L) | 1 | 773 | 258 | TCA (S) => TTA (L) | 1 |
|  | 791 | 264 | CCC (P) => CTC (L) | 1 | 791 | 264 | CCC (P) => CTC (L) | 1 |
| *atp*B | 322 | 108 | CTT (L) => TTT (F) | 1 | 322 | 108 | CTT (L) => TTT (F) | 1 |
|  | 434 | 145 | TCC (S) => TTC (F) | 1 | 434 | 145 | TCC (S) => TTC (F) | 1 |
|  | 542 | 181 | CCC (P) => CTC (L) | 1 | 542 | 181 | CCC (P) => CTC (L) | 1 |
|  | 839 | 280 | TCC (S) => TTC (F) | 1 | 839 | 280 | TCC (S) => TTC (F) | 1 |
|  | 899 | 300 | GCC (A) => GTC (V) | 1 | 899 | 300 | GCC (A) => GTC (V) | 1 |
|  | 1247 | 416 | ACC (T) => ATC (I) | 1 | 1247 | 416 | ACC (T) => ATC (I) | 1 |
| *atp*F | 0 |  |  |  | 0 |  |  |  |
| *atp*I | 629 | 210 | TCA (S) => TTA (L) | 1 | 629 | 210 | TCA (S) => TTA (L) | 1 |
| *ccs*A | 26 | 9 | ACC (T) => ATC (I) | 1 | 26 | 9 | ACC (T) => ATC (I) | 1 |
|  | 40 | 14 | CCC (P) => TCC (S) | 1 | 40 | 14 | CCC (P) => TCC (S) | 1 |
|  | 173 | 58 | CCA (P) => CTA (L) | 1 | 173 | 58 | CCA (P) => CTA (L) | 1 |
|  | 205 | 69 | CCA (P) => TCA (S) | 1 | 205 | 69 | CCA (P) => TCA (S) | 1 |
| *clp*P | 146 | 49 | CCC (P) => CTC (L) | 1 | 146 | 49 | CCC (P) => CTC (L) | 1 |
|  | 164 | 55 | ACA (T) => ATA (I) | 0.86 | 164 | 55 | ACA (T) => ATA (I) | 0.86 |
|  | 191 | 64 | TCA (S) => TTA (L) | 0.86 | 191 | 64 | TCA (S) => TTA (L) | 0.86 |
|  | 323 | 108 | TCT (S) => TTT (F) | 1 | 323 | 108 | TCT (S) => TTT (F) | 1 |
|  | 329 | 110 | CCT (P) => CTT (L) | 1 | 329 | 110 | CCT (P) => CTT (L) | 1 |
|  | 542 | 181 | TCC (S) => TTC (F) | 1 | 542 | 181 | TCC (S) => TTC (F) | 1 |
| *mat*K | 68 | 23 | CCT (P) => CTT (L) | 1 | 68 | 23 | CCT (P) => CTT (L) | 1 |
|  | 365 | 122 | CCC (P) => CTC (L) | 1 | 365 | 122 | CCC (P) => CTC (L) | 1 |
|  | 482 | 161 | ACT (T) => ATT (I) | 1 | 482 | 161 | ACT (T) => ATT (I) | 1 |
|  | 568 | 190 | CTT (L) => TTT (F) | 0.86 | 568 | 190 | CTT (L) => TTT (F) | 0.86 |
|  | 718 | 240 | CCA (P) => TCA (S) | 1 | 718 | 240 | CCA (P) => TCA (S) | 1 |
|  | 739 | 247 | CCT (P) => TCT (S) | 1 | 739 | 247 | CCT (P) => TCT (S) | 1 |
|  | 1168 | 390 | CCC (P) => TTC (F) | 1 | 1168 | 390 | CCC (P) => TTC (F) | 1 |
|  | 1169 | 390 | CCC (P) => TTC (F) | 1 | 1169 | 390 | CCC (P) => TTC (F) | 1 |
| *ndh*A | 107 | 36 | CCT (P) => CTT (L) | 1 | 107 | 36 | CCT (P) => CTT (L) | 1 |
|  | 566 | 189 | TCA (S) => TTA (L) | 1 | 566 | 189 | TCA (S) => TTA (L) | 1 |
|  | 1073 | 358 | TCC (S) => TTC (F) | 1 | 1073 | 358 | TCC (S) => TTC (F) | 1 |
| *ndh*B | 149 | 50 | TCA (S) => TTA (L) | 1 | 149 | 50 | TCA (S) => TTA (L) | 1 |
|  | 467 | 156 | CCA (P) => CTA (L) | 1 | 467 | 156 | CCA (P) => CTA (L) | 1 |
|  | 586 | 196 | CAT (H) => TAT (Y) | 1 | 586 | 196 | CAT (H) => TAT (Y) | 1 |
|  | 611 | 204 | TCA (S) => TTA (L) | 0.8 | 611 | 204 | TCA (S) => TTA (L) | 0.8 |
|  | 737 | 246 | CCA (P) => CTA (L) | 1 | 737 | 246 | CCA (P) => CTA (L) | 1 |
|  | 746 | 249 | TCT (S) => TTT (F) | 1 | 746 | 249 | TCT (S) => TTT (F) | 1 |
|  | 1499 | 500 | TCA (S) => TTA (L) | 1 | 830 | 277 | TCA (S) => TTA (L) | 1 |
|  | 1505 | 502 | TCA (S) => TTA (L) | 1 | 836 | 279 | TCA (S) => TTA (L) | 1 |
|  | 2150 | 717 | CCA (P) => CTA (L) | 1 | 1481 | 494 | CCA (P) => CTA (L) | 1 |
| *ndh*D | 263 | 88 | TCA (S) => TTA (L) | 1 | 263 | 88 | TCA (S) => TTA (L) | 1 |
|  | 479 | 160 | TCA (S) => TTA (L) | 1 | 479 | 160 | TCA (S) => TTA (L) | 1 |
|  | 758 | 253 | TCA (S) => TTA (L) | 1 | 758 | 253 | TCA (S) => TTA (L) | 1 |
|  | 767 | 256 | CCC (P) => CTC (L) | 1 | 767 | 256 | CCC (P) => CTC (L) | 1 |
|  | 1178 | 393 | TCA (S) => TTA (L) | 0.8 | 1178 | 393 | TCA (S) => TTA (L) | 0.8 |
| *ndh*F | 290 | 97 | TCA (S) => TTA (L) | 1 | 290 | 97 | TCA (S) => TTA (L) | 1 |
| *ndh*G | 166 | 56 | CAT (H) => TAT (Y) | 0.8 | 166 | 56 | CAT (H) => TAT (Y) | 0.8 |
|  | 314 | 105 | ACA (T) => ATA (I) | 0.8 | 314 | 105 | ACA (T) => ATA (I) | 0.8 |
| *pet*B | 418 | 140 | CGG (R) => TGG (W) | 1 | 418 | 140 | CGG (R) => TGG (W) | 1 |
|  | 611 | 204 | CCA (P) => CTA (L) | 1 | 611 | 204 | CCA (P) => CTA (L) | 1 |
| *pet*D | 0 |  |  |  | 0 |  |  |  |
| *pet*G | 0 |  |  |  | 0 |  |  |  |
| *pet*L | 0 |  |  |  | 0 |  |  |  |
| *psa*B | 349 | 117 | CCA (P) => TCA (S) | 1 | 349 | 117 | CCA (P) => TCA (S) | 1 |
|  | 424 | 142 | CCA (P) => TCA (S) | 1 | 424 | 142 | CCA (P) => TCA (S) | 1 |
|  | 788 | 263 | CCC (P) => CTC (L) | 1 | 788 | 263 | CCC (P) => CTC (L) | 1 |
|  | 1202 | 401 | GCC (A) => GTC (V) | 1 | 1202 | 401 | GCC (A) => GTC (V) | 1 |
|  | 1268 | 423 | CCC (P) => CTC (L) | 1 | 1268 | 423 | CCC (P) => CTC (L) | 1 |
|  | 1307 | 436 | ACT (T) => ATT (I) | 1 | 1307 | 436 | ACT (T) => ATT (I) | 1 |
|  | 1342 | 448 | CCC (P) => TCC (S) | 1 | 1342 | 448 | CCC (P) => TCC (S) | 1 |
|  | 1487 | 496 | ACT (T) => ATT (I) | 1 | 1487 | 496 | ACT (T) => ATT (I) | 1 |
|  | 1889 | 630 | TCG (S) => TTG (L) | 1 | 1889 | 630 | TCG (S) => TTG (L) | 1 |
|  | 1981 | 661 | CTC (L) => TTC (F) | 1 | 1981 | 661 | CTC (L) => TTC (F) | 1 |
| *psa*I | 0 |  |  |  | 0 |  |  |  |
| *psb*B | 0 |  |  |  | 0 |  |  |  |
| *psb*E | 101 | 34 | CCG (P) => CTG (L) | 1 | 101 | 34 | CCG (P) => CTG (L) | 1 |
|  | 107 | 36 | GCT (A) => GTT (V) | 1 | 107 | 36 | GCT (A) => GTT (V) | 1 |
|  |  |  |  |  |  |  |  |  |
| *psb*F | 0 |  |  |  | 0 |  |  |  |
| *psb*L | 58 | 20 | CCA (P) => TCA (S) | 1 | 58 | 20 | CCA (P) => TCA (S) | 1 |
| *rpl*2 | 407 | 136 | ACG (T) => ATG (M) | 1 | 407 | 136 | ACG (T) => ATG (M) | 1 |
|  | 446 | 149 | ACT (T) => ATT (I) | 1 | 446 | 149 | ACT (T) => ATT (I) | 1 |
|  | 659 | 220 | GCA (A) => GTA (V) | 0.86 | 659 | 220 | GCA (A) => GTA (V) | 0.86 |
|  | 677 | 226 | ACG (T) => ATG (M) | 1 | 677 | 226 | ACG (T) => ATG (M) | 1 |
| *rpl*20 | 163 | 55 | CCC (P) => TCC (S) | 1 | 163 | 55 | CCC (P) => TCC (S) | 1 |
|  | 284 | 95 | TCG (S) => TTG (L) | 1 | 284 | 95 | TCG (S) => TTG (L) | 1 |
|  | 305 | 102 | CCC (P) => CTC (L) | 0.86 | 305 | 102 | CCC (P) => CTC (L) | 0.86 |
|  | 364 | 122 | CCG (P) => TCG (S) | 0.86 | 364 | 122 | CCG (P) => TCG (S) | 0.86 |
|  |  |  |  |  |  |  |  |  |
| *rpl*23 | 58 | 20 | CAT (H) => TAT (Y) | 1 | 58 | 20 | CAT (H) => TAT (Y) | 1 |
|  | 230 | 77 | CCC (P) => CTC (L) | 1 | 230 | 77 | CCC (P) => CTC (L) | 1 |
| *rpo*A | 88 | 30 | CAT (H) => TAT (Y) | 0.86 | 88 | 30 | CAT (H) => TAT (Y) | 0.86 |
|  | 694 | 232 | CTC (L) => TTC (F) | 0.86 | 694 | 232 | CTC (L) => TTC (F) | 0.86 |
|  | 704 | 235 | TCC (S) => TTC (F) | 1 | 704 | 235 | TCC (S) => TTC (F) | 1 |
|  | 860 | 287 | TCG (S) => TTG (L) | 1 | 860 | 287 | TCG (S) => TTG (L) | 1 |
|  | 934 | 312 | CTT (L) => TTT (F) | 1 | 934 | 312 | CTT (L) => TTT (F) | 1 |
|  | 959 | 320 | ACA (T) => ATA (I) | 0.86 | 959 | 320 | ACA (T) => ATA (I) | 0.86 |
| *rpo*B | 983 | 328 | GCT (A) => GTT (V) | 1 | 983 | 328 | GCT (A) => GTT (V) | 1 |
| *rpo*C1 | 508 | 170 | CCC (P) => TCC (S) | 1 | 508 | 170 | CCC (P) => TCC (S) | 1 |
|  | 1589 | 530 | GCA (A) => GTA (V) | 0.86 | 1589 | 530 | GCA (A) => GTA (V) | 0.86 |
|  | 1747 | 583 | CCC (P) => TCC (S) | 0.86 | 1747 | 583 | CCC (P) => TCC (S) | 0.86 |
|  | 2042 | 681 | CCA (P) => CTA (L) | 1 | 2042 | 681 | CCA (P) => CTA (L) | 1 |
| *rpo*C2 | 2084 | 695 | GCA (A) => GTA (V) | 1 | 2084 | 695 | GCA (A) => GTA (V) | 1 |
|  | 3722 | 1241 | TCG (S) => TTG (L) | 0.86 | 3722 | 1241 | TCG (S) => TTG (L) | 0.86 |
| *rps*2 | 248 | 83 | TCA (S) => TTA (L) | 1 | 248 | 83 | TCA (S) => TTA (L) | 1 |
| *rps*8 | 94 | 32 | CCC (P) => TCC (S) | 0.86 | 94 | 32 | CCC (P) => TCC (S) | 0.86 |
|  | 104 | 35 | ACC (T) => ATC (I) | 1 | 104 | 35 | ACC (T) => ATC (I) | 1 |
|  | 200 | 67 | CCT (P) => CTT (L) | 1 | 200 | 67 | CCT (P) => CTT (L) | 1 |
|  | 206 | 69 | CCG (P) => CTG (L) | 0.86 | 206 | 69 | CCG (P) => CTG (L) | 0.86 |
|  | 278 | 93 | ACC (T) => ATC (I) | 1 | 278 | 93 | ACC (T) => ATC (I) | 1 |
|  | 332 | 111 | TCG (S) => TTG (L) | 1 | 332 | 111 | TCG (S) => TTG (L) | 1 |
|  | 403 | 135 | CAT (H) => TAT (Y) | 1 | 403 | 135 | CAT (H) => TAT (Y) | 1 |
| *rps*14 | 230 | 77 | TCG (S) => TTG (L) | 1 | 230 | 77 | TCG (S) => TTG (L) | 1 |
| *rps*16 | 82 | 28 | CCT (P) => TCT (S) | 0.8 | 82 | 28 | CCT (P) => TCT (S) | 0.8 |
|  | 104 | 35 | CCT (P) => CTT (L) | 1 | 104 | 35 | CCT (P) => CTT (L) | 1 |
| *ycf*3 | 31 | 11 | CTC (L) => TTC (F) | 1 | 31 | 11 | CTC (L) => TTC (F) | 1 |
|  | 110 | 37 | TCC (S) => TTC (F) | 1 | 110 | 37 | TCC (S) => TTC (F) | 1 |
|  | 350 | 117 | ACA (T) => ATA (I) | 1 | 350 | 117 | ACA (T) => ATA (I) | 1 |
|  | 503 | 168 | TCC (S) => TTC (F) | 1 | 503 | 168 | TCC (S) => TTC (F) | 1 |
|  |  |  |  |  |  |  |  |  |
| Total | 104 sites |  |  |  | 104 sites |  |  |  |

| **5. *Dendroseris pruinata*** | | | | | ***6. Dendroseris micrantha*** | | | |
| --- | --- | --- | --- | --- | --- | --- | --- | --- |
| Gene | Nucleotide Position | Amino Acid Position | Codon Conversion | Score | Nucleotide Position | Amino Acid Position | Codon Conversion | Score |
| *acc*D | 370 | 124 | CCT (P) => TCT (S) | 1 | 370 | 124 | CCT (P) => TCT (S) | 1 |
|  | 451 | 151 | CAC (H) => TAC (Y) | 1 | 451 | 151 | CAC (H) => TAC (Y) | 1 |
|  | 1231 | 411 | CCA (P) => TCA (S) | 1 | 1231 | 411 | CCA (P) => TCA (S) | 1 |
|  | 1439 | 480 | CCT (P) => CTT (L) | 1 | 1439 | 480 | CCT (P) => CTT (L) | 1 |
| *atp*A | 773 | 258 | TCA (S) => TTA (L) | 1 | 773 | 258 | TCA (S) => TTA (L) | 1 |
|  | 791 | 264 | CCC (P) => CTC (L) | 1 | 791 | 264 | CCC (P) => CTC (L) | 1 |
| *atp*B | 322 | 108 | CTT (L) => TTT (F) | 1 | 322 | 108 | CTT (L) => TTT (F) | 1 |
|  | 434 | 145 | TCC (S) => TTC (F) | 1 | 434 | 145 | TCC (S) => TTC (F) | 1 |
|  | 542 | 181 | CCC (P) => CTC (L) | 1 | 542 | 181 | CCC (P) => CTC (L) | 1 |
|  | 839 | 280 | TCC (S) => TTC (F) | 1 | 839 | 280 | TCC (S) => TTC (F) | 1 |
|  | 899 | 300 | GCC (A) => GTC (V) | 1 | 899 | 300 | GCC (A) => GTC (V) | 1 |
|  | 1247 | 416 | ACC (T) => ATC (I) | 1 | 1247 | 416 | ACC (T) => ATC (I) | 1 |
| *atp*F | 0 |  |  |  | 0 |  |  |  |
| *atp*I | 629 | 210 | TCA (S) => TTA (L) | 1 | 629 | 210 | TCA (S) => TTA (L) | 1 |
| *ccs*A | 26 | 9 | ACC (T) => ATC (I) | 1 | 26 | 9 | ACC (T) => ATC (I) | 1 |
|  | 40 | 14 | CCC (P) => TCC (S) | 1 | 40 | 14 | CCC (P) => TCC (S) | 1 |
|  | 173 | 58 | CCA (P) => CTA (L) | 1 | 173 | 58 | CCA (P) => CTA (L) | 1 |
|  | 205 | 69 | CCA (P) => TCA (S) | 1 | 205 | 69 | CCA (P) => TCA (S) | 1 |
| *clp*P | 146 | 49 | CCC (P) => CTC (L) | 1 | 146 | 49 | CCC (P) => CTC (L) | 1 |
|  | 164 | 55 | ACA (T) => ATA (I) | 0.86 | 164 | 55 | ACA (T) => ATA (I) | 0.86 |
|  | 191 | 64 | TCA (S) => TTA (L) | 0.86 | 191 | 64 | TCA (S) => TTA (L) | 0.86 |
|  | 323 | 108 | TCT (S) => TTT (F) | 1 | 323 | 108 | TCT (S) => TTT (F) | 1 |
|  | 329 | 110 | CCT (P) => CTT (L) | 1 | 329 | 110 | CCT (P) => CTT (L) | 1 |
|  | 542 | 181 | TCC (S) => TTC (F) | 1 | 542 | 181 | TCC (S) => TTC (F) | 1 |
| *mat*K | 68 | 23 | CCT (P) => CTT (L) | 1 | 68 | 23 | CCT (P) => CTT (L) | 1 |
|  | 365 | 122 | CCC (P) => CTC (L) | 1 | 365 | 122 | CCC (P) => CTC (L) | 1 |
|  | 482 | 161 | ACT (T) => ATT (I) | 1 | 482 | 161 | ACT (T) => ATT (I) | 1 |
|  | 568 | 190 | CTT (L) => TTT (F) | 0.86 | 568 | 190 | CTT (L) => TTT (F) | 0.86 |
|  | 718 | 240 | CCA (P) => TCA (S) | 1 | 718 | 240 | CCA (P) => TCA (S) | 1 |
|  | 739 | 247 | CCT (P) => TCT (S) | 1 | 739 | 247 | CCT (P) => TCT (S) | 1 |
|  | 1168 | 390 | CCC (P) => TTC (F) | 1 | 1168 | 390 | CCC (P) => TTC (F) | 1 |
|  | 1169 | 390 | CCC (P) => TTC (F) | 1 | 1169 | 390 | CCC (P) => TTC (F) | 1 |
| *ndh*A | 107 | 36 | CCT (P) => CTT (L) | 1 | 107 | 36 | CCT (P) => CTT (L) | 1 |
|  | 566 | 189 | TCA (S) => TTA (L) | 1 | 566 | 189 | TCA (S) => TTA (L) | 1 |
|  | 1073 | 358 | TCC (S) => TTC (F) | 1 | 1073 | 358 | TCC (S) => TTC (F) | 1 |
| *ndh*B | 149 | 50 | TCA (S) => TTA (L) | 1 | 149 | 50 | TCA (S) => TTA (L) | 1 |
|  | 467 | 156 | CCA (P) => CTA (L) | 1 | 467 | 156 | CCA (P) => CTA (L) | 1 |
|  | 586 | 196 | CAT (H) => TAT (Y) | 1 | 586 | 196 | CAT (H) => TAT (Y) | 1 |
|  | 611 | 204 | TCA (S) => TTA (L) | 0.8 | 611 | 204 | TCA (S) => TTA (L) | 0.8 |
|  | 737 | 246 | CCA (P) => CTA (L) | 1 | 737 | 246 | CCA (P) => CTA (L) | 1 |
|  | 746 | 249 | TCT (S) => TTT (F) | 1 | 746 | 249 | TCT (S) => TTT (F) | 1 |
|  | 830 | 277 | TCA (S) => TTA (L) | 1 | 830 | 277 | TCA (S) => TTA (L) | 1 |
|  | 836 | 279 | TCA (S) => TTA (L) | 1 | 836 | 279 | TCA (S) => TTA (L) | 1 |
|  | 1481 | 494 | CCA (P) => CTA (L) | 1 | 1481 | 494 | CCA (P) => CTA (L) | 1 |
| *ndh*D | 263 | 88 | TCA (S) => TTA (L) | 1 | 263 | 88 | TCA (S) => TTA (L) | 1 |
|  | 479 | 160 | TCA (S) => TTA (L) | 1 | 479 | 160 | TCA (S) => TTA (L) | 1 |
|  | 758 | 253 | TCA (S) => TTA (L) | 1 | 758 | 253 | TCA (S) => TTA (L) | 1 |
|  | 767 | 256 | CCC (P) => CTC (L) | 1 | 767 | 256 | CCC (P) => CTC (L) | 1 |
|  | 1178 | 393 | TCA (S) => TTA (L) | 0.8 | 1178 | 393 | TCA (S) => TTA (L) | 0.8 |
| *ndh*F | 290 | 97 | TCA (S) => TTA (L) | 1 | 290 | 97 | TCA (S) => TTA (L) | 1 |
| *ndh*G | 166 | 56 | CAT (H) => TAT (Y) | 0.8 | 166 | 56 | CAT (H) => TAT (Y) | 0.8 |
|  | 314 | 105 | ACA (T) => ATA (I) | 0.8 | 314 | 105 | ACA (T) => ATA (I) | 0.8 |
| *pet*B | 418 | 140 | CGG (R) => TGG (W) | 1 | 418 | 140 | CGG (R) => TGG (W) | 1 |
|  | 611 | 204 | CCA (P) => CTA (L) | 1 | 611 | 204 | CCA (P) => CTA (L) | 1 |
| *pet*D | 0 |  |  |  | 0 |  |  |  |
| *pet*G | 0 |  |  |  | 0 |  |  |  |
| *pet*L | 0 |  |  |  | 0 |  |  |  |
| *psa*B | 349 | 117 | CCA (P) => TCA (S) | 1 | 349 | 117 | CCA (P) => TCA (S) | 1 |
|  | 424 | 142 | CCA (P) => TCA (S) | 1 | 424 | 142 | CCA (P) => TCA (S) | 1 |
|  | 788 | 263 | CCC (P) => CTC (L) | 1 | 788 | 263 | CCC (P) => CTC (L) | 1 |
|  | 1202 | 401 | GCC (A) => GTC (V) | 1 | 1202 | 401 | GCC (A) => GTC (V) | 1 |
|  | 1268 | 423 | CCC (P) => CTC (L) | 1 | 1268 | 423 | CCC (P) => CTC (L) | 1 |
|  | 1307 | 436 | ACT (T) => ATT (I) | 1 | 1307 | 436 | ACT (T) => ATT (I) | 1 |
|  | 1342 | 448 | CCC (P) => TCC (S) | 1 | 1342 | 448 | CCC (P) => TCC (S) | 1 |
|  | 1487 | 496 | ACT (T) => ATT (I) | 1 | 1487 | 496 | ACT (T) => ATT (I) | 1 |
|  | 1889 | 630 | TCG (S) => TTG (L) | 1 | 1889 | 630 | TCG (S) => TTG (L) | 1 |
|  | 1981 | 661 | CTC (L) => TTC (F) | 1 | 1981 | 661 | CTC (L) => TTC (F) | 1 |
| *psa*I | 0 |  |  |  | 0 |  |  |  |
| *psb*B | 0 |  |  |  | 0 |  |  |  |
| *psb*E | 101 | 34 | CCG (P) => CTG (L) | 1 | 101 | 34 | CCG (P) => CTG (L) | 1 |
|  | 107 | 36 | GCT (A) => GTT (V) | 1 | 107 | 36 | GCT (A) => GTT (V) | 1 |
| *psb*F | 0 |  |  |  | 0 |  |  |  |
| *psb*L | 58 | 20 | CCA (P) => TCA (S) | 1 | 58 | 20 | CCA (P) => TCA (S) | 1 |
| *rpl*2 | 407 | 136 | ACG (T) => ATG (M) | 1 | 407 | 136 | ACG (T) => ATG (M) | 1 |
|  | 446 | 149 | ACT (T) => ATT (I) | 1 | 446 | 149 | ACT (T) => ATT (I) | 1 |
|  | 659 | 220 | GCA (A) => GTA (V) | 0.86 | 659 | 220 | GCA (A) => GTA (V) | 0.86 |
|  |  |  |  |  | 677 | 226 | ACG (T) => ATG (M) | 1 |
| *rpl*20 | 677 | 226 | ACG (T) => ATG (M) | 1 | 163 | 55 | CCC (P) => TCC (S) | 1 |
|  | 163 | 55 | CCC (P) => TCC (S) | 1 | 284 | 95 | TCG (S) => TTG (L) | 1 |
|  | 284 | 95 | TCG (S) => TTG (L) | 1 | 305 | 102 | CCC (P) => CTC (L) | 0.86 |
|  | 305 | 102 | CCC (P) => CTC (L) | 0.86 | 364 | 122 | CCG (P) => TCG (S) | 0.86 |
|  | 364 | 122 | CCG (P) => TCG (S) | 0.86 |  |  |  |  |
| *rpl*23 | 58 | 20 | CAT (H) => TAT (Y) | 1 | 58 | 20 | CAT (H) => TAT (Y) | 1 |
|  | 230 | 77 | CCC (P) => CTC (L) | 1 | 230 | 77 | CCC (P) => CTC (L) | 1 |
| *rpo*A | 88 | 30 | CAT (H) => TAT (Y) | 0.86 | 88 | 30 | CAT (H) => TAT (Y) | 0.86 |
|  | 694 | 232 | CTC (L) => TTC (F) | 0.86 | 694 | 232 | CTC (L) => TTC (F) | 0.86 |
|  | 704 | 235 | TCC (S) => TTC (F) | 1 | 704 | 235 | TCC (S) => TTC (F) | 1 |
|  | 860 | 287 | TCG (S) => TTG (L) | 1 | 860 | 287 | TCG (S) => TTG (L) | 1 |
|  | 934 | 312 | CTT (L) => TTT (F) | 1 | 934 | 312 | CTT (L) => TTT (F) | 1 |
|  | 959 | 320 | ACA (T) => ATA (I) | 0.86 | 959 | 320 | ACA (T) => ATA (I) | 0.86 |
| *rpo*B | 983 | 328 | GCT (A) => GTT (V) | 1 | 983 | 328 | GCT (A) => GTT (V) | 1 |
| *rpo*C1 | 508 | 170 | CCC (P) => TCC (S) | 1 | 508 | 170 | CCC (P) => TCC (S) | 1 |
|  | 1589 | 530 | GCA (A) => GTA (V) | 0.86 | 1589 | 530 | GCA (A) => GTA (V) | 0.86 |
|  | 1747 | 583 | CCC (P) => TCC (S) | 0.86 | 1747 | 583 | CCC (P) => TCC (S) | 0.86 |
|  | 2042 | 681 | CCA (P) => CTA (L) | 1 | 2042 | 681 | CCA (P) => CTA (L) | 1 |
| *rpo*C2 | 2084 | 695 | GCA (A) => GTA (V) | 1 | 2084 | 695 | GCA (A) => GTA (V) | 1 |
|  | 3722 | 1241 | TCG (S) => TTG (L) | 0.86 | 3722 | 1241 | TCG (S) => TTG (L) | 0.86 |
| *rps*2 | 248 | 83 | TCA (S) => TTA (L) | 1 | 248 | 83 | TCA (S) => TTA (L) | 1 |
| *rps*8 | 94 | 32 | CCC (P) => TCC (S) | 0.86 | 94 | 32 | CCC (P) => TCC (S) | 0.86 |
|  | 104 | 35 | ACC (T) => ATC (I) | 1 | 104 | 35 | ACC (T) => ATC (I) | 1 |
|  | 200 | 67 | CCT (P) => CTT (L) | 1 | 200 | 67 | CCT (P) => CTT (L) | 1 |
|  | 206 | 69 | CCG (P) => CTG (L) | 0.86 | 206 | 69 | CCG (P) => CTG (L) | 0.86 |
|  | 278 | 93 | ACC (T) => ATC (I) | 1 | 278 | 93 | ACC (T) => ATC (I) | 1 |
|  | 332 | 111 | TCG (S) => TTG (L) | 1 | 332 | 111 | TCG (S) => TTG (L) | 1 |
|  | 403 | 135 | CAT (H) => TAT (Y) | 1 | 403 | 135 | CAT (H) => TAT (Y) | 1 |
| *rps*14 | 230 | 77 | TCG (S) => TTG (L) | 1 | 230 | 77 | TCG (S) => TTG (L) | 1 |
| *rps*16 | 130 | 44 | CTT (L) => TTT (F) | 1 | 130 | 44 | CTT (L) => TTT (F) | 1 |
|  |  |  |  |  |  |  |  |  |
| *ycf*3 | 31 | 11 | CTC (L) => TTC (F) | 1 | 31 | 11 | CTC (L) => TTC (F) | 1 |
|  | 110 | 37 | TCC (S) => TTC (F) | 1 | 110 | 37 | TCC (S) => TTC (F) | 1 |
|  | 350 | 117 | ACA (T) => ATA (I) | 1 | 350 | 117 | ACA (T) => ATA (I) | 1 |
|  | 503 | 168 | TCC (S) => TTC (F) | 1 | 503 | 168 | TCC (S) => TTC (F) | 1 |
|  |  |  |  |  |  |  |  |  |
| Total | 103 sites |  |  |  | 103 sites |  |  |  |

| **7. *Dendroseris berteroana*** | | | | | ***8. Dendroseris pinnata*** | | | |
| --- | --- | --- | --- | --- | --- | --- | --- | --- |
| Gene | Nucleotide Position | Amino Acid Position | Codon Conversion | Score | Nucleotide Position | Amino Acid Position | Codon Conversion | Score |
| *acc*D | 370 | 124 | CCT (P) => TCT (S) | 1 | 370 | 124 | CCT (P) => TCT (S) | 1 |
|  | 1231 | 411 | CCA (P) => TCA (S) | 1 | 451 | 151 | CAC (H) => TAC (Y) | 1 |
|  | 1439 | 480 | CCT (P) => CTT (L) | 1 | 1231 | 411 | CCA (P) => TCA (S) | 1 |
|  |  |  |  |  | 1439 | 480 | CCT (P) => CTT (L) | 1 |
| *atp*A | 773 | 258 | TCA (S) => TTA (L) | 1 | 773 | 258 | TCA (S) => TTA (L) | 1 |
|  | 791 | 264 | CCC (P) => CTC (L) | 1 | 791 | 264 | CCC (P) => CTC (L) | 1 |
| *atp*B | 322 | 108 | CTT (L) => TTT (F) | 1 | 322 | 108 | CTT (L) => TTT (F) | 1 |
|  | 434 | 145 | TCC (S) => TTC (F) | 1 | 434 | 145 | TCC (S) => TTC (F) | 1 |
|  | 542 | 181 | CCC (P) => CTC (L) | 1 | 542 | 181 | CCC (P) => CTC (L) | 1 |
|  | 839 | 280 | TCC (S) => TTC (F) | 1 | 839 | 280 | TCC (S) => TTC (F) | 1 |
|  | 899 | 300 | GCC (A) => GTC (V) | 1 | 899 | 300 | GCC (A) => GTC (V) | 1 |
|  | 1247 | 416 | ACC (T) => ATC (I) | 1 | 1247 | 416 | ACC (T) => ATC (I) | 1 |
| *atp*F | 0 |  |  |  | 0 |  |  |  |
| *atp*I | 629 | 210 | TCA (S) => TTA (L) | 1 | 629 | 210 | TCA (S) => TTA (L) | 1 |
| *ccs*A | 26 | 9 | ACC (T) => ATC (I) | 1 | 26 | 9 | ACC (T) => ATC (I) | 1 |
|  | 40 | 14 | CCC (P) => TCC (S) | 1 | 40 | 14 | CCC (P) => TCC (S) | 1 |
|  | 173 | 58 | CCA (P) => CTA (L) | 1 | 173 | 58 | CCA (P) => CTA (L) | 1 |
|  | 205 | 69 | CCA (P) => TCA (S) | 1 | 205 | 69 | CCA (P) => TCA (S) | 1 |
| *clp*P | 146 | 49 | CCC (P) => CTC (L) | 1 | 146 | 49 | CCC (P) => CTC (L) | 1 |
|  | 164 | 55 | ACA (T) => ATA (I) | 0.86 | 164 | 55 | ACA (T) => ATA (I) | 0.86 |
|  | 191 | 64 | TCA (S) => TTA (L) | 0.86 | 191 | 64 | TCA (S) => TTA (L) | 0.86 |
|  | 323 | 108 | TCT (S) => TTT (F) | 1 | 323 | 108 | TCT (S) => TTT (F) | 1 |
|  | 329 | 110 | CCT (P) => CTT (L) | 1 | 329 | 110 | CCT (P) => CTT (L) | 1 |
|  | 542 | 181 | TCC (S) => TTC (F) | 1 | 542 | 181 | TCC (S) => TTC (F) | 1 |
| *mat*K | 68 | 23 | CCT (P) => CTT (L) | 1 | 68 | 23 | CCT (P) => CTT (L) | 1 |
|  | 365 | 122 | CCC (P) => CTC (L) | 1 | 365 | 122 | CCC (P) => CTC (L) | 1 |
|  | 482 | 161 | ACT (T) => ATT (I) | 1 | 482 | 161 | ACT (T) => ATT (I) | 1 |
|  | 568 | 190 | CTT (L) => TTT (F) | 0.86 | 568 | 190 | CTT (L) => TTT (F) | 0.86 |
|  | 718 | 240 | CCA (P) => TCA (S) | 1 | 718 | 240 | CCA (P) => TCA (S) | 1 |
|  | 739 | 247 | CCT (P) => TCT (S) | 1 | 739 | 247 | CCT (P) => TCT (S) | 1 |
|  | 1168 | 390 | CCC (P) => TTC (F) | 1 | 1168 | 390 | CCC (P) => TTC (F) | 1 |
|  | 1169 | 390 | CCC (P) => TTC (F) | 1 | 1169 | 390 | CCC (P) => TTC (F) | 1 |
| *ndh*A | 107 | 36 | CCT (P) => CTT (L) | 1 | 107 | 36 | CCT (P) => CTT (L) | 1 |
|  | 566 | 189 | TCA (S) => TTA (L) | 1 | 566 | 189 | TCA (S) => TTA (L) | 1 |
|  | 1073 | 358 | TCC (S) => TTC (F) | 1 | 1073 | 358 | TCC (S) => TTC (F) | 1 |
| *ndh*B | 149 | 50 | TCA (S) => TTA (L) | 1 | 149 | 50 | TCA (S) => TTA (L) | 1 |
|  | 467 | 156 | CCA (P) => CTA (L) | 1 | 467 | 156 | CCA (P) => CTA (L) | 1 |
|  | 586 | 196 | CAT (H) => TAT (Y) | 1 | 586 | 196 | CAT (H) => TAT (Y) | 1 |
|  | 611 | 204 | TCA (S) => TTA (L) | 0.8 | 611 | 204 | TCA (S) => TTA (L) | 0.8 |
|  | 737 | 246 | CCA (P) => CTA (L) | 1 | 737 | 246 | CCA (P) => CTA (L) | 1 |
|  | 746 | 249 | TCT (S) => TTT (F) | 1 | 746 | 249 | TCT (S) => TTT (F) | 1 |
|  | 830 | 277 | TCA (S) => TTA (L) | 1 | 830 | 277 | TCA (S) => TTA (L) | 1 |
|  | 836 | 279 | TCA (S) => TTA (L) | 1 | 836 | 279 | TCA (S) => TTA (L) | 1 |
|  | 1481 | 494 | CCA (P) => CTA (L) | 1 | 1481 | 494 | CCA (P) => CTA (L) | 1 |
|  |  |  |  |  |  |  |  |  |
| *ndh*D | 263 | 88 | TCA (S) => TTA (L) | 1 | 263 | 88 | TCA (S) => TTA (L) | 1 |
|  | 479 | 160 | TCA (S) => TTA (L) | 1 | 479 | 160 | TCA (S) => TTA (L) | 1 |
|  | 758 | 253 | TCA (S) => TTA (L) | 1 | 758 | 253 | TCA (S) => TTA (L) | 1 |
|  | 767 | 256 | CCC (P) => CTC (L) | 1 | 767 | 256 | CCC (P) => CTC (L) | 1 |
|  | 1178 | 393 | TCA (S) => TTA (L) | 0.8 | 1178 | 393 | TCA (S) => TTA (L) | 0.8 |
| *ndh*F | 290 | 97 | TCA (S) => TTA (L) | 1 | 290 | 97 | TCA (S) => TTA (L) | 1 |
| *ndh*G | 166 | 56 | CAT (H) => TAT (Y) | 0.8 | 166 | 56 | CAT (H) => TAT (Y) | 0.8 |
|  | 314 | 105 | ACA (T) => ATA (I) | 0.8 | 314 | 105 | ACA (T) => ATA (I) | 0.8 |
| *pet*B | 418 | 140 | CGG (R) => TGG (W) | 1 | 418 | 140 | CGG (R) => TGG (W) | 1 |
|  | 611 | 204 | CCA (P) => CTA (L) | 1 | 611 | 204 | CCA (P) => CTA (L) | 1 |
| *pet*D | 0 |  |  |  | 0 |  |  |  |
| *pet*G | 0 |  |  |  | 0 |  |  |  |
| *pet*L | 0 |  |  |  | 0 |  |  |  |
| *psa*B | 349 | 117 | CCA (P) => TCA (S) | 1 | 349 | 117 | CCA (P) => TCA (S) | 1 |
|  | 424 | 142 | CCA (P) => TCA (S) | 1 | 424 | 142 | CCA (P) => TCA (S) | 1 |
|  | 788 | 263 | CCC (P) => CTC (L) | 1 | 788 | 263 | CCC (P) => CTC (L) | 1 |
|  | 1202 | 401 | GCC (A) => GTC (V) | 1 | 1202 | 401 | GCC (A) => GTC (V) | 1 |
|  | 1268 | 423 | CCC (P) => CTC (L) | 1 | 1268 | 423 | CCC (P) => CTC (L) | 1 |
|  | 1307 | 436 | ACT (T) => ATT (I) | 1 | 1307 | 436 | ACT (T) => ATT (I) | 1 |
|  | 1342 | 448 | CCC (P) => TCC (S) | 1 | 1342 | 448 | CCC (P) => TCC (S) | 1 |
|  | 1487 | 496 | ACT (T) => ATT (I) | 1 | 1487 | 496 | ACT (T) => ATT (I) | 1 |
|  | 1889 | 630 | TCG (S) => TTG (L) | 1 | 1889 | 630 | TCG (S) => TTG (L) | 1 |
|  | 1981 | 661 | CTC (L) => TTC (F) | 1 | 1981 | 661 | CTC (L) => TTC (F) | 1 |
| *psa*I | 0 |  |  |  | 0 |  |  |  |
| *psb*B | 0 |  |  |  | 0 |  |  |  |
| *psb*E | 101 | 34 | CCG (P) => CTG (L) | 1 | 101 | 34 | CCG (P) => CTG (L) | 1 |
|  | 107 | 36 | GCT (A) => GTT (V) | 1 | 107 | 36 | GCT (A) => GTT (V) | 1 |
|  |  |  |  |  |  |  |  |  |
| *psb*F | 0 |  |  |  | 0 |  |  |  |
| *psb*L | 58 | 20 | CCA (P) => TCA (S) | 1 | 58 | 20 | CCA (P) => TCA (S) | 1 |
| *rpl*2 | 407 | 136 | ACG (T) => ATG (M) | 1 | 407 | 136 | ACG (T) => ATG (M) | 1 |
|  | 446 | 149 | ACT (T) => ATT (I) | 1 | 446 | 149 | ACT (T) => ATT (I) | 1 |
|  | 659 | 220 | GCA (A) => GTA (V) | 0.86 | 659 | 220 | GCA (A) => GTA (V) | 0.86 |
|  | 677 | 226 | ACG (T) => ATG (M) | 1 | 677 | 226 | ACG (T) => ATG (M) | 1 |
| *rpl*20 | 163 | 55 | CCC (P) => TCC (S) | 1 | 163 | 55 | CCC (P) => TCC (S) | 1 |
|  | 284 | 95 | TCG (S) => TTG (L) | 1 | 284 | 95 | TCG (S) => TTG (L) | 1 |
|  | 305 | 102 | CCC (P) => CTC (L) | 0.86 | 305 | 102 | CCC (P) => CTC (L) | 0.86 |
|  | 364 | 122 | CCG (P) => TCG (S) | 0.86 | 364 | 122 | CCG (P) => TCG (S) | 0.86 |
|  |  |  |  |  |  |  |  |  |
| *rpl*23 | 58 | 20 | CAT (H) => TAT (Y) | 1 | 58 | 20 | CAT (H) => TAT (Y) | 1 |
|  | 230 | 77 | CCC (P) => CTC (L) | 1 | 230 | 77 | CCC (P) => CTC (L) | 1 |
| *rpo*A | 88 | 30 | CAT (H) => TAT (Y) | 0.86 | 88 | 30 | CAT (H) => TAT (Y) | 0.86 |
|  | 694 | 232 | CTC (L) => TTC (F) | 0.86 | 694 | 232 | CTC (L) => TTC (F) | 0.86 |
|  | 704 | 235 | TCC (S) => TTC (F) | 1 | 704 | 235 | TCC (S) => TTC (F) | 1 |
|  | 827 | 276 | ACA (T) => ATA (I) | 1 | 860 | 287 | TCG (S) => TTG (L) | 1 |
|  |  |  |  |  | 934 | 312 | CTT (L) => TTT (F) | 1 |
|  |  |  |  |  | 959 | 320 | ACA (T) => ATA (I) | 0.86 |
| *rpo*B | 983 | 328 | GCT (A) => GTT (V) | 1 | 983 | 328 | GCT (A) => GTT (V) | 1 |
| *rpo*C1 | 508 | 170 | CCC (P) => TCC (S) | 1 | 508 | 170 | CCC (P) => TCC (S) | 1 |
|  | 1589 | 530 | GCA (A) => GTA (V) | 0.86 | 1589 | 530 | GCA (A) => GTA (V) | 0.86 |
|  | 1747 | 583 | CCC (P) => TCC (S) | 0.86 | 1747 | 583 | CCC (P) => TCC (S) | 0.86 |
|  | 2042 | 681 | CCA (P) => CTA (L) | 1 | 2042 | 681 | CCA (P) => CTA (L) | 1 |
| *rpo*C2 | 2084 | 695 | GCA (A) => GTA (V) | 1 | 2084 | 695 | GCA (A) => GTA (V) | 1 |
|  | 3722 | 1241 | TCG (S) => TTG (L) | 0.86 | 3722 | 1241 | TCG (S) => TTG (L) | 0.86 |
| *rps*2 | 248 | 83 | TCA (S) => TTA (L) | 1 | 248 | 83 | TCA (S) => TTA (L) | 1 |
| *rps*8 | 94 | 32 | CCC (P) => TCC (S) | 0.86 | 94 | 32 | CCC (P) => TCC (S) | 0.86 |
|  | 104 | 35 | ACC (T) => ATC (I) | 1 | 104 | 35 | ACC (T) => ATC (I) | 1 |
|  | 200 | 67 | CCT (P) => CTT (L) | 1 | 200 | 67 | CCT (P) => CTT (L) | 1 |
|  | 206 | 69 | CCG (P) => CTG (L) | 0.86 | 206 | 69 | CCG (P) => CTG (L) | 0.86 |
|  | 278 | 93 | ACC (T) => ATC (I) | 1 | 278 | 93 | ACC (T) => ATC (I) | 1 |
|  | 332 | 111 | TCG (S) => TTG (L) | 1 | 332 | 111 | TCG (S) => TTG (L) | 1 |
|  | 403 | 135 | CAT (H) => TAT (Y) | 1 | 403 | 135 | CAT (H) => TAT (Y) | 1 |
| *rps*14 | 230 | 77 | TCG (S) => TTG (L) | 1 | 230 | 77 | TCG (S) => TTG (L) | 1 |
| *rps*16 | 0 |  |  |  | 0 |  |  |  |
| *ycf*3 | 31 | 11 | CTC (L) => TTC (F) | 1 | 31 | 11 | CTC (L) => TTC (F) | 1 |
|  | 110 | 37 | TCC (S) => TTC (F) | 1 | 110 | 37 | TCC (S) => TTC (F) | 1 |
|  | 350 | 117 | ACA (T) => ATA (I) | 1 | 350 | 117 | ACA (T) => ATA (I) | 1 |
|  | 503 | 168 | TCC (S) => TTC (F) | 1 | 503 | 168 | TCC (S) => TTC (F) | 1 |
|  |  |  |  |  |  |  |  |  |
| Total | 99 sites |  |  |  | 102 Sites |  |  |  |

| ***9. Sonchus asper*** | | | | | ***10. Sonchus canariensis*** | | | |
| --- | --- | --- | --- | --- | --- | --- | --- | --- |
| Gene | Nucleotide Position | Amino Acid Position | Codon Conversion | Score | Nucleotide Position | Amino Acid Position | Codon Conversion | Score |
| *acc*D | 370 | 124 | CCT (P) => TCT (S) | 1 | 370 | 124 | CCT (P) => TCT (S) | 1 |
|  | 451 | 151 | CAC (H) => TAC (Y) | 1 | 451 | 151 | CAC (H) => TAC (Y) | 1 |
|  | 1231 | 411 | CCA (P) => TCA (S) | 1 | 1231 | 411 | CCA (P) => TCA (S) | 1 |
|  | 1439 | 480 | CCT (P) => CTT (L) | 1 | 1439 | 480 | CCT (P) => CTT (L) | 1 |
| *atp*A | 773 | 258 | TCA (S) => TTA (L) | 1 | 773 | 258 | TCA (S) => TTA (L) | 1 |
|  | 791 | 264 | CCC (P) => CTC (L) | 1 | 791 | 264 | CCC (P) => CTC (L) | 1 |
| *atp*B | 322 | 108 | CTT (L) => TTT (F) | 1 | 322 | 108 | CTT (L) => TTT (F) | 1 |
|  | 434 | 145 | TCC (S) => TTC (F) | 1 | 434 | 145 | TCC (S) => TTC (F) | 1 |
|  | 542 | 181 | CCC (P) => CTC (L) | 1 | 542 | 181 | CCC (P) => CTC (L) | 1 |
|  | 839 | 280 | TCC (S) => TTC (F) | 1 | 839 | 280 | TCC (S) => TTC (F) | 1 |
|  | 899 | 300 | GCC (A) => GTC (V) | 1 | 899 | 300 | GCC (A) => GTC (V) | 1 |
|  | 1247 | 416 | ACC (T) => ATC (I) | 1 | 1247 | 416 | ACC (T) => ATC (I) | 1 |
| *atp*F | 0 |  |  |  | 0 |  |  |  |
| *atp*I | 629 | 210 | TCA (S) => TTA (L) | 1 | 629 | 210 | TCA (S) => TTA (L) | 1 |
| *ccs*A | 26 | 9 | ACC (T) => ATC (I) | 1 | 26 | 9 | ACC (T) => ATC (I) | 1 |
|  | 40 | 14 | CCC (P) => TCC (S) | 1 | 40 | 14 | CCC (P) => TCC (S) | 1 |
|  | 205 | 69 | CCA (P) => TCA (S) | 1 | 173 | 58 | CCA (P) => CTA (L) | 1 |
|  |  |  |  |  | 205 | 69 | CCA (P) => TCA (S) | 1 |
| *clp*P | 146 | 49 | CCC (P) => CTC (L) | 1 | 146 | 49 | CCC (P) => CTC (L) | 1 |
|  | 164 | 55 | ACA (T) => ATA (I) | 0.86 | 164 | 55 | ACA (T) => ATA (I) | 0.86 |
|  | 191 | 64 | TCA (S) => TTA (L) | 0.86 | 191 | 64 | TCA (S) => TTA (L) | 0.86 |
|  | 323 | 108 | TCT (S) => TTT (F) | 1 | 323 | 108 | TCT (S) => TTT (F) | 1 |
|  | 329 | 110 | CCT (P) => CTT (L) | 1 | 329 | 110 | CCT (P) => CTT (L) | 1 |
|  | 542 | 181 | TCC (S) => TTC (F) | 1 | 542 | 181 | TCC (S) => TTC (F) | 1 |
| *mat*K | 68 | 23 | CCT (P) => CTT (L) | 1 | 68 | 23 | CCT (P) => CTT (L) | 1 |
|  | 365 | 122 | CCC (P) => CTC (L) | 1 | 365 | 122 | CCC (P) => CTC (L) | 1 |
|  | 482 | 161 | ACT (T) => ATT (I) | 1 | 482 | 161 | ACT (T) => ATT (I) | 1 |
|  | 718 | 240 | CCA (P) => TCA (S) | 1 | 568 | 190 | CTT (L) => TTT (F) | 0.86 |
|  | 1168 | 390 | CCC (P) => TTC (F) | 1 | 718 | 240 | CCA (P) => TCA (S) | 1 |
|  | 1169 | 390 | CCC (P) => TTC (F) | 1 | 739 | 247 | CCT (P) => TCT (S) | 1 |
|  |  |  |  |  | 1168 | 390 | CCC (P) => TTC (F) | 1 |
|  |  |  |  |  | 1169 | 390 | CCC (P) => TTC (F) | 1 |
| *ndh*A | 107 | 36 | CCT (P) => CTT (L) | 1 | 107 | 36 | CCT (P) => CTT (L) | 1 |
|  | 566 | 189 | TCA (S) => TTA (L) | 1 | 566 | 189 | TCA (S) => TTA (L) | 1 |
|  | 1073 | 358 | TCC (S) => TTC (F) | 1 | 1073 | 358 | TCC (S) => TTC (F) | 1 |
| *ndh*B | 149 | 50 | TCA (S) => TTA (L) | 1 | 149 | 50 | TCA (S) => TTA (L) | 1 |
|  | 259 | 87 | CTT (L) => TTT (F) | 1 | 467 | 156 | CCA (P) => CTA (L) | 1 |
|  | 467 | 156 | CCA (P) => CTA (L) | 1 | 586 | 196 | CAT (H) => TAT (Y) | 1 |
|  | 586 | 196 | CAT (H) => TAT (Y) | 1 | 611 | 204 | TCA (S) => TTA (L) | 0.8 |
|  | 611 | 204 | TCA (S) => TTA (L) | 0.8 | 737 | 246 | CCA (P) => CTA (L) | 1 |
|  | 737 | 246 | CCA (P) => CTA (L) | 1 | 746 | 249 | TCT (S) => TTT (F) | 1 |
|  | 746 | 249 | TCT (S) => TTT (F) | 1 | 830 | 277 | TCA (S) => TTA (L) | 1 |
|  | 830 | 277 | TCA (S) => TTA (L) | 1 | 836 | 279 | TCA (S) => TTA (L) | 1 |
|  | 836 | 279 | TCA (S) => TTA (L) | 1 | 1481 | 494 | CCA (P) => CTA (L) | 1 |
|  | 1481 | 494 | CCA (P) => CTA (L) | 1 |  |  |  |  |
| *ndh*D | 263 | 88 | TCA (S) => TTA (L) | 1 | 263 | 88 | TCA (S) => TTA (L) | 1 |
|  | 479 | 160 | TCA (S) => TTA (L) | 1 | 479 | 160 | TCA (S) => TTA (L) | 1 |
|  | 758 | 253 | TCA (S) => TTA (L) | 1 | 758 | 253 | TCA (S) => TTA (L) | 1 |
|  | 767 | 256 | CCC (P) => CTC (L) | 1 | 767 | 256 | CCC (P) => CTC (L) | 1 |
|  | 1178 | 393 | TCA (S) => TTA (L) | 0.8 | 1178 | 393 | TCA (S) => TTA (L) | 0.8 |
| *ndh*F | 290 | 97 | TCA (S) => TTA (L) | 1 | 290 | 97 | TCA (S) => TTA (L) | 1 |
| ndhG | 166 | 56 | CAT (H) => TAT (Y) | 0.8 | 166 | 56 | CAT (H) => TAT (Y) | 0.8 |
|  | 314 | 105 | ACA (T) => ATA (I) | 0.8 | 314 | 105 | ACA (T) => ATA (I) | 0.8 |
| *pet*B | 418 | 140 | CGG (R) => TGG (W) | 1 | 418 | 140 | CGG (R) => TGG (W) | 1 |
|  | 611 | 204 | CCA (P) => CTA (L) | 1 | 611 | 204 | CCA (P) => CTA (L) | 1 |
| *pet*D | 0 |  |  |  | 0 |  |  |  |
| *pet*G | 0 |  |  |  | 0 |  |  |  |
| *pet*L | 0 |  |  |  | 0 |  |  |  |
| *psa*B | 349 | 117 | CCA (P) => TCA (S) | 1 | 349 | 117 | CCA (P) => TCA (S) | 1 |
|  | 424 | 142 | CCA (P) => TCA (S) | 1 | 424 | 142 | CCA (P) => TCA (S) | 1 |
|  | 788 | 263 | CCC (P) => CTC (L) | 1 | 788 | 263 | CCC (P) => CTC (L) | 1 |
|  | 1202 | 401 | GCC (A) => GTC (V) | 1 | 1202 | 401 | GCC (A) => GTC (V) | 1 |
|  | 1268 | 423 | CCC (P) => CTC (L) | 1 | 1268 | 423 | CCC (P) => CTC (L) | 1 |
|  | 1307 | 436 | ACT (T) => ATT (I) | 1 | 1307 | 436 | ACT (T) => ATT (I) | 1 |
|  | 1342 | 448 | CCC (P) => TCC (S) | 1 | 1342 | 448 | CCC (P) => TCC (S) | 1 |
|  | 1487 | 496 | ACT (T) => ATT (I) | 1 | 1487 | 496 | ACT (T) => ATT (I) | 1 |
|  | 1889 | 630 | TCG (S) => TTG (L) | 1 | 1889 | 630 | TCG (S) => TTG (L) | 1 |
|  | 1981 | 661 | CTC (L) => TTC (F) | 1 | 1981 | 661 | CTC (L) => TTC (F) | 1 |
| *psa*I | 0 |  |  |  | 0 |  |  |  |
| *psb*B | 0 |  |  |  | 0 |  |  |  |
| *psb*E | 101 | 34 | CCG (P) => CTG (L) | 1 | 101 | 34 | CCG (P) => CTG (L) | 1 |
|  | 107 | 36 | GCT (A) => GTT (V) | 1 | 107 | 36 | GCT (A) => GTT (V) | 1 |
|  |  |  |  |  |  |  |  |  |
| *psb*F | 0 |  |  |  | 0 |  |  |  |
| *psb*L | 58 | 20 | CCA (P) => TCA (S) | 1 | 58 | 20 | CCA (P) => TCA (S) | 1 |
| *rpl*2 | 407 | 136 | ACG (T) => ATG (M) | 1 | 407 | 136 | ACG (T) => ATG (M) | 1 |
|  | 659 | 220 | GCA (A) => GTA (V) | 0.86 | 446 | 149 | ACT (T) => ATT (I) | 1 |
|  | 677 | 226 | ACG (T) => ATG (M) | 1 | 659 | 220 | GCA (A) => GTA (V) | 0.86 |
|  |  |  |  |  | 677 | 226 | ACG (T) => ATG (M) | 1 |
| *rpl*20 | 308 | 103 | CCG (P) => CTG (L) | 0.86 | 163 | 55 | CCC (P) => TCC (S) | 1 |
|  |  |  |  |  | 284 | 95 | TCG (S) => TTG (L) | 1 |
|  |  |  |  |  | 305 | 102 | CCC (P) => CTC (L) | 0.86 |
|  |  |  |  |  | 364 | 122 | CCG (P) => TCG (S) | 0.86 |
| *rpl*23 | 58 | 20 | CAT (H) => TAT (Y) | 1 | 58 | 20 | CAT (H) => TAT (Y) | 1 |
|  | 230 | 77 | CCC (P) => CTC (L) | 1 | 230 | 77 | CCC (P) => CTC (L) | 1 |
| *rpo*A | 88 | 30 | CAT (H) => TAT (Y) | 0.86 | 88 | 30 | CAT (H) => TAT (Y) | 0.86 |
|  | 694 | 232 | CTC (L) => TTC (F) | 0.86 | 694 | 232 | CTC (L) => TTC (F) | 0.86 |
|  | 704 | 235 | TCC (S) => TTC (F) | 1 | 704 | 235 | TCC (S) => TTC (F) | 1 |
|  | 860 | 287 | TCG (S) => TTG (L) | 1 | 860 | 287 | TCG (S) => TTG (L) | 1 |
|  | 934 | 312 | CTT (L) => TTT (F) | 1 | 934 | 312 | CTT (L) => TTT (F) | 1 |
|  | 959 | 320 | ACA (T) => ATA (I) | 0.86 | 959 | 320 | ACA (T) => ATA (I) | 0.86 |
| *rpo*B | 983 | 328 | GCT (A) => GTT (V) | 1 | 983 | 328 | GCT (A) => GTT (V) | 1 |
| *rpo*C1 | 508 | 170 | CCC (P) => TCC (S) | 1 | 508 | 170 | CCC (P) => TCC (S) | 1 |
|  | 1589 | 530 | GCA (A) => GTA (V) | 0.86 | 1589 | 530 | GCA (A) => GTA (V) | 0.86 |
|  | 1747 | 583 | CCC (P) => TCC (S) | 0.86 | 1747 | 583 | CCC (P) => TCC (S) | 0.86 |
|  | 2042 | 681 | CCA (P) => CTA (L) | 1 | 2042 | 681 | CCA (P) => CTA (L) | 1 |
| *rpo*C2 | 2093 | 698 | GCA (A) => GTA (V) | 1 | 2084 | 695 | GCA (A) => GTA (V) | 1 |
|  | 3731 | 1244 | TCG (S) => TTG (L) | 0.86 | 3722 | 1241 | TCG (S) => TTG (L) | 0.86 |
| *rps*2 | 248 | 83 | TCA (S) => TTA (L) | 1 | 248 | 83 | TCA (S) => TTA (L) | 1 |
| *rps*8 | 94 | 32 | CCC (P) => TCC (S) | 0.86 | 94 | 32 | CCC (P) => TCC (S) | 0.86 |
|  | 104 | 35 | ACC (T) => ATC (I) | 1 | 104 | 35 | ACC (T) => ATC (I) | 1 |
|  | 200 | 67 | CCT (P) => CTT (L) | 1 | 200 | 67 | CCT (P) => CTT (L) | 1 |
|  | 206 | 69 | CCG (P) => CTG (L) | 0.86 | 206 | 69 | CCG (P) => CTG (L) | 0.86 |
|  | 278 | 93 | ACC (T) => ATC (I) | 1 | 278 | 93 | ACC (T) => ATC (I) | 1 |
|  | 332 | 111 | TCG (S) => TTG (L) | 1 | 332 | 111 | TCG (S) => TTG (L) | 1 |
|  | 403 | 135 | CAT (H) => TAT (Y) | 1 | 403 | 135 | CAT (H) => TAT (Y) | 1 |
| *rps*14 | 230 | 77 | TCG (S) => TTG (L) | 1 | 230 | 77 | TCG (S) => TTG (L) | 1 |
| *rps1*6 | 82 | 28 | CCT (P) => TCT (S) | 0.8 | 82 | 28 | CCT (P) => TCT (S) | 0.8 |
|  | 104 | 35 | CCT (P) => CTT (L) | 1 | 104 | 35 | CCT (P) => CTT (L) | 1 |
| *ycf*3 | 31 | 11 | CTC (L) => TTC (F) | 1 | 31 | 11 | CTC (L) => TTC (F) | 1 |
|  | 110 | 37 | TCC (S) => TTC (F) | 1 | 110 | 37 | TCC (S) => TTC (F) | 1 |
|  | 350 | 117 | ACA (T) => ATA (I) | 1 | 350 | 117 | ACA (T) => ATA (I) | 1 |
|  | 503 | 168 | TCC (S) => TTC (F) | 1 | 503 | 168 | TCC (S) => TTC (F) | 1 |
|  |  |  |  |  |  |  |  |  |
| Total | 98 sites |  |  |  | 104 Sites |  |  |  |

**
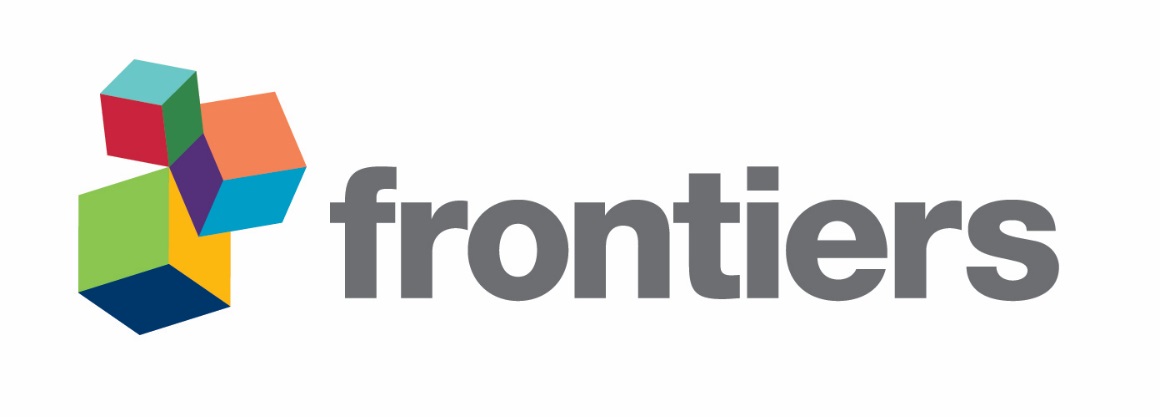
**
