# Supplementary material for: Plastid Phylogenomics of Dendroseris (Cichorieae; Asteraceae): Insights Into Structural Organization and Molecular Evolution of an Endemic Lineage From the Juan Fernández Islands
Source: Front Plant Sci. 2020 Nov 5;11:594272. doi: 10.3389/fpls.2020.594272 (PMC7674203; doi:10.3389/fpls.2020.594272)
Supplement: Supplementary file 1 [file Data_Sheet_1.zip › Table 7 (20).DOCX]

Supplementary Material

Plastid phylogenomics of *Dendroseris* (Cichorieae; Asteraceae), endemic to the Juan Fernández Islands: Insights into structural organization and molecular evolution

**Myong-Suk Cho^1^, Seon-Hee Kim^1^, JiYoung Yang^2^, Daniel J. Crawford^3^, Tod F. Stuessy^4^, Patricio López-Sepúlveda^5^, and Seung-Chul Kim^1*^**

*** Correspondence**: Seung-Chul Kim: [sonchus96@skku.edu](mailto:sonchus96@skku.edu) or sonchus2009@gmail.com

# Supplementary Figures and Tables

## 1.2 Supplementary Tables

**Supplementary Table 3.** The frequency of SSR candidates of seven *Dendroseris* and *Reichardia ligulata*.

| **Repeat type of SSR (considering sequence complementary)** | **Frequency** | | | | | | | | **Percentage frequency (%)** |
| --- | --- | --- | --- | --- | --- | --- | --- | --- | --- |
|  | ***R. ligulata*** | ***D. litoralis*** | ***D. macrantha*** | ***D. marginata*** | ***D. pruinata*** | ***D. micrantha*** | ***D. berteroana*** | ***D. pinnata*** |  |
| **Mononucleotide** |  |  |  |  |  |  |  |  |  |
| A/T | 2 | 2 | 2 | 2 | 2 | 2 | 2 | 1 | 3 |
| **Dinucleotide** |  |  |  |  |  |  |  |  |  |
| AT/AT | 3 | 4 | 4 | 4 | 4 | 4 | 4 | 4 | 5 |
| **Trinucleotide** |  |  |  |  |  |  |  |  |  |
| AAC/GTT | 4 | 4 | 4 | 4 | 4 | 4 | 4 | 4 | 5 |
| AAG/CTT | 21 | 22 | 22 | 21 | 21 | 21 | 21 | 21 | 29 |
| AAT/ATT | 20 | 22 | 22 | 22 | 22 | 22 | 22 | 22 | 30 |
| ACC/GGT | 2 | 2 | 2 | 2 | 2 | 2 | 2 | 2 | 3 |
| ACG/CGT | 1 | 1 | 1 | 1 | 1 | 1 | 2 | 1 | 2 |
| ACT/AGT | 1 | 1 | 1 | 1 | 1 | 1 | 1 | 1 | 1 |
| AGC/CTG | 7 | 7 | 7 | 7 | 7 | 7 | 7 | 7 | 10 |
| AGG/CCT | 4 | 2 | 2 | 2 | 2 | 2 | 2 | 2 | 3 |
| ATC/ATG | 3 | 3 | 3 | 3 | 3 | 3 | 3 | 3 | 4 |
| **Tetranucleotide** |  |  |  |  |  |  |  |  |  |
| AAAG/CTTT | 1 | 1 | 1 | 1 | 1 | 1 | 1 | 1 | 1 |
| AAAT/ATTT | 1 | 2 | 2 | 2 | 2 | 2 | 2 | 2 | 3 |
| AATC/ATTG | 1 | 1 | 1 | 1 |  |  | 1 | 1 | 1 |

**
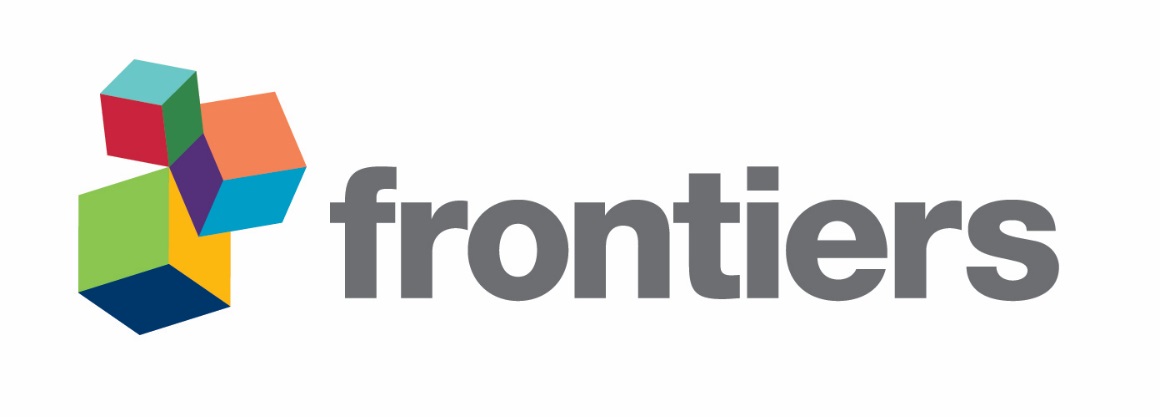
**
