# Supplementary material for: Plastid Phylogenomics of Dendroseris (Cichorieae; Asteraceae): Insights Into Structural Organization and Molecular Evolution of an Endemic Lineage From the Juan Fernández Islands
Source: Front Plant Sci. 2020 Nov 5;11:594272. doi: 10.3389/fpls.2020.594272 (PMC7674203; doi:10.3389/fpls.2020.594272)
Supplement: Supplementary file 1 [file Data_Sheet_1.zip › Table 8 (7).DOCX]

Supplementary Material

Plastid phylogenomics of *Dendroseris* (Cichorieae; Asteraceae), endemic to the Juan Fernández Islands: Insights into structural organization and molecular evolution

**Myong-Suk Cho^1^, Seon-Hee Kim^1^, JiYoung Yang^2^, Daniel J. Crawford^3^, Tod F. Stuessy^4^, Patricio López-Sepúlveda^5^, and Seung-Chul Kim^1*^**

*** Correspondence**: Seung-Chul Kim: [sonchus96@skku.edu](mailto:sonchus96@skku.edu) or sonchus2009@gmail.com

# Supplementary Figures and Tables

## 1.2 Supplementary Tables

**
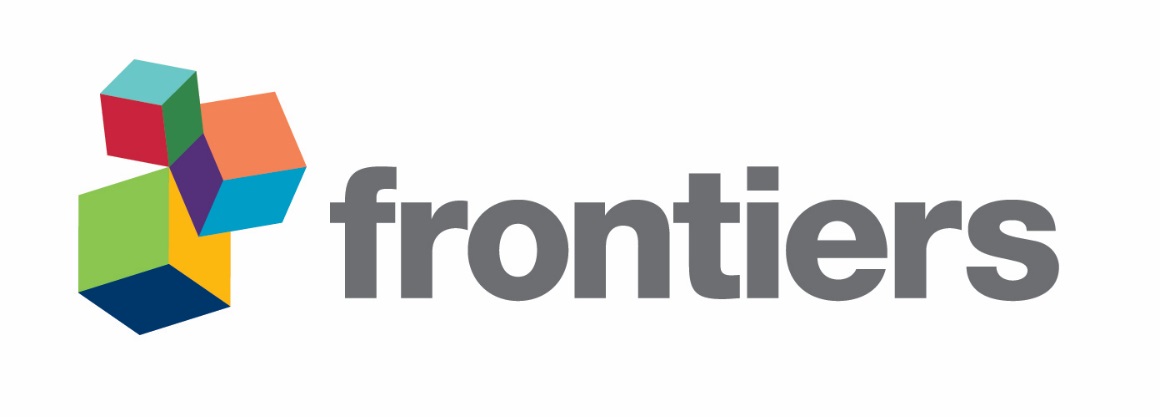
**

**Supplementary Table 4.** The SSR candidates of seven *Dedroseris* species and *Reichardia ligulata* chloroplast genomes. The repeated nucleotide sequences of SSR repeat motifs are indicated inside brackets, and their repeat numbers are marked outside brackets.

|  | **SSR type** | **SSR repeat motifs and repeat numbers** | | | | | | | | **size** | **CDS/non-coding gene/intergenic region** | **quadri-partite region** |
| --- | --- | --- | --- | --- | --- | --- | --- | --- | --- | --- | --- | --- |
|  |  | ***R. ligulata*** | ***D. litoralis*** | ***D. macrantha*** | ***D. marginata*** | ***D. pruinata*** | ***D. micrantha*** | ***D. berteroana*** | ***D. pinnata*** |  |  |  |
|  |  |  |  |  |  |  |  |  |  |  |  |  |
| 1 | Trinucleotide | (TTC)3 | (TTC)3 | (TTC)3 | (TTC)3 | (TTC)3 | (TTC)3 | (TTC)3 | (TTC)3 | 9 | psbA | LSC |
| 2 | Trinucleotide | (TTC)3 | (TTC)3 | (TTC)3 | (TTC)3 | (TTC)3 | (TTC)3 | (TTC)3 | (TTC)3 | 9 | matK | LSC |
| 3 | Trinucleotide | (GAA)3 | (GAA)3 | (GAA)3 | (GAA)3 | (GAA)3 | (GAA)3 | (GAA)3 | (GAA)3 | 9 | matK | LSC |
| 4 | Tetranucleotide | (AAAT)3 | (AAAT)3 | (AAAT)3 | (AAAT)3 | (AAAT)3 | (AAAT)3 | (AAAT)3 | (AAAT)3 | 12 | trnK-rps16 | LSC |
| 5 | Trinucleotide | (AAG)3 | (AAG)3 | (AAG)3 | (AAG)3 | (AAG)3 | (AAG)3 | (AAG)3 | (AAG)3 | 9 | trnK-rps17 | LSC |
| 6 | Trinucleotide | (ATT)3 |  |  |  |  |  |  |  | 9 | trnC-petN | LSC |
| 7 | Trinucleotide |  | (ATA)3 | (ATA)3 | (ATA)3 | (ATA)3 | (ATA)3 | (ATA)3 | (ATA)3 | 9 | rps16-trnQ | LSC |
| 8 | Trinucleotide |  |  |  | (TCG)3 | (TCG)3 | (TCG)3 | (TCG)3 | (TCG)3 | 9 | trnQ-psbK | LSC |
| 9 | Mononucleotide |  |  |  |  |  |  | (T)18 |  | 18 | trnE-rpoB | LSC |
| 10 | Trinucleotide | (TCG)3 | (TCG)3 | (TCG)3 | (GAA)3 | (GAA)3 | (GAA)3 | (GAA)3 | (GAA)3 | 9 | psbM-trnD | LSC |
| 11 | Mononucleotide | (T)17 |  |  |  |  |  |  |  | 17 | trnB-rpoB | LSC |
| 12 | Trinucleotide | (GAA)3 | (GAA)3 | (GAA)3 |  |  |  |  |  | 9 | rpoC1 | LSC |
| 13 | Dinucleotide | (TA)5 | (TA)5 | (TA)5 | (TA)5 | (TA)5 | (TA)5 | (TA)5 | (TA)5 | 10 | rpoC1 | LSC |
| 14 | Dinucleotide | (AT)5 | (AT)5 | (AT)5 | (AT)5 | (AT)5 | (AT)5 | (AT)5 | (AT)5 | 10 | rpoC2 | LSC |
| 15 | Trinucleotide | (TAA)3 | (TAA)3 | (TAA)3 | (TAA)3 | (TAA)3 | (TAA)3 | (TAA)3 | (TAA)3 | 9 | rps2-atpI | LSC |
| 16 | Trinucleotide | (GTT)3 | (GTT)3 | (GTT)3 | (GTT)3 | (GTT)3 | (GTT)3 | (GTT)3 | (GTT)3 | 9 | atpI | LSC |
| 17 | Dinucleotide |  | (AT)8 | (AT)8 | (AT)8 | (AT)6 | (AT)6 | (AT)8 | (AT)8 | 12 | atpH-atpF | LSC |
| 18 | Trinucleotide | (TAA)3 | (TAA)3 | (TAA)3 | (TAA)3 | (TAA)3 | (TAA)3 | (TAA)3 | (TAA)3 | 9 | trnT-psbD | LSC |
| 19 | Trinucleotide | (GGA)3 |  |  |  |  |  |  |  | 9 | psbC | LSC |
| 20 | Trinucleotide | (TTC)4 | (TTC)4 | (TTC)4 | (TTC)4 | (TTC)4 | (TTC)4 | (TTC)4 | (TTC)4 | 12 | psbC | LSC |
| 21 | Trinucleotide | (ATG)3 | (ATG)3 | (ATG)3 | (ATG)3 | (ATG)3 | (ATG)3 | (ATG)3 | (ATG)3 | 9 | psaB | LSC |
| 22 | Trinucleotide | (GCA)3 | (GCA)3 | (GCA)3 | (GCA)3 | (GCA)3 | (GCA)3 | (GCA)3 | (GCA)3 | 9 | psaA | LSC |
| 23 | Trinucleotide |  | (CTT)3 | (CTT)3 | (CTT)3 | (CTT)3 | (CTT)3 | (CTT)3 | (CTT)3 | 9 | rps4-trnT | LSC |
| 24 | Trinucleotide | (ATA)3 | (ATA)3 | (ATA)3 | (ATA)3 | (ATA)3 | (ATA)3 | (ATA)3 | (ATA)3 | 9 | trnF-ndhJ | LSC |
| 25 | Mononucleotide | (T)18 |  |  |  |  |  |  |  | 18 | trnT-trnL | LSC |
| 26 | Trinucleotide | (TTA)3 | (TTA)3 | (TTA)3 | (TTA)3 | (TTA)3 | (TTA)3 | (TTA)3 | (TTA)3 | 9 | trnC non-coding | LSC |
| 27 | Trinucleotide | (TAA)3 |  |  |  |  |  |  |  |  | atpB-rbcL | LSC |
| 28 | Trinucleotide | (TTG)3 |  |  |  |  |  |  |  |  | atpB-rbcL | LSC |
| 29 | Tetranucleotide | (TTG)4 | (AAAT)3 | (AAAT)3 | (AAAT)3 | (AAAT)3 | (AAAT)3 | (AAAT)3 | (AAAT)3 | 12 | atpB-rbcL | LSC |
| 30 | Trinucleotide | (TTG)5 | (TTG)3 | (TTG)3 | (TTG)3 | (TTG)3 | (TTG)3 | (TTG)3 | (TTG)3 | 9 | atpB-rbcL | LSC |
| 31 | Trinucleotide | (GCT)3 | (GCT)3 | (GCT)3 | (GCT)3 | (GCT)3 | (GCT)3 | (GCT)3 | (GCT)3 | 9 | rbcL | LSC |
| 32 | Trinucleotide | (TTA)3 | (TTA)3 | (TTA)3 | (TTA)3 | (TTA)3 | (TTA)3 | (TTA)3 | (TTA)3 | 9 | rbcL-accD | LSC |
| 33 | Trinucleotide |  |  |  |  |  |  | (CGA)3 |  |  | accD | LSC |
| 34 | Trinucleotide | (GGA)3 | (GGA)3 | (GGA)3 | (GGA)3 | (GGA)3 | (GGA)3 | (GGA)3 | (GGA)3 | 9 | accD | LSC |
| 35 | Trinucleotide | (TAA)3 | (TAA)4 | (TAA)4 | (TAA)4 | (TAA)4 | (TAA)4 | (TAA)4 | (TAA)4 | 12 | accD-psaI | LSC |
| 36 | Trinucleotide | (AAT)3 | (AAT)3 | (AAT)3 | (AAT)3 | (AAT)3 | (AAT)3 | (AAT)3 | (AAT)3 | 9 | accD-psaI | LSC |
| 37 | Trinucleotide | (AGG)3 |  |  |  |  |  |  |  |  | ycf4-cemA | LSC |
| 38 | Trinucleotide |  | (AAT)3 | (AAT)3 | (AAT)3 | (AAT)3 | (AAT)3 | (AAT)3 | (AAT)3 | 9 | petA-psbJ | LSC |
| 39 | Mononucleotide |  | (A)15 | (A)15 | (A)16 | (A)15 | (A)15 |  |  |  | psbE-petL | LSC |
| 40 | Trinucleotide | (CCT)3 | (CCT)3 | (CCT)3 | (CCT)3 | (CCT)3 | (CCT)3 | (CCT)3 | (CCT)3 | 9 | petG-trnW | LSC |
| 41 | Trinucleotide | (ATA)3 | (ATA)3 | (ATA)3 | (ATA)3 | (ATA)3 | (ATA)3 | (ATA)3 | (ATA)3 | 9 | trnP-psaJ | LSC |
| 42 | Dinucleotide | (TA)8 | (TA)8 | (TA)8 | (TA)8 | (TA)8 | (TA)8 | (TA)8 | (TA)8 | 16 | rpl33-rps18 | LSC |
| 43 | Trinucleotide | (AAC)3 | (AAC)3 | (AAC)3 | (AAC)3 | (AAC)3 | (AAC)3 | (AAC)3 | (AAC)3 | 9 | rps18 | LSC |
| 44 | Trinucleotide |  | (AAT)3 | (AAT)3 | (AAT)3 | (AAT)3 | (AAT)3 | (AAT)3 | (AAT)3 | 9 | rps18-rpl20 | LSC |
| 45 | Trinucleotide | (GAA)3 | (GAA)3 | (GAA)3 | (GAA)3 | (GAA)3 | (GAA)3 | (GAA)3 | (GAA)3 | 9 | rps18-rpl20 | LSC |
| 46 | Trinucleotide | (TTG)3 | (TTG)3 | (TTG)3 | (TTG)3 | (TTG)3 | (TTG)3 | (TTG)3 | (TTG)3 | 9 | psbB | LSC |
| 47 | Trinucleotide | (TCT)3 | (TCT)3 | (TCT)3 | (TCT)3 | (TCT)3 | (TCT)3 | (TCT)3 | (TCT)3 | 9 | psbB | LSC |
| 48 | Trinucleotide | (CTG)3 | (CTG)3 | (CTG)3 | (CTG)3 | (CTG)3 | (CTG)3 | (CTG)3 | (CTG)3 | 9 | rpoA | LSC |
| 49 | Trinucleotide | (ATA)3 | (ATA)3 | (ATA)3 | (ATA)3 | (ATA)3 | (ATA)3 | (ATA)3 | (ATA)3 | 9 | rpoA | LSC |
| 50 | Tetranucleotide | (TTTC)3 | (TTTC)3 | (TTTC)3 | (TTTC)3 | (TTTC)3 | (TTTC)3 | (TTTC)3 | (TTTC)3 | 12 | rpl16 intron | LSC |
| 51 | Trinucleotide | (TGC)3 | (TGC)3 | (TGC)3 | (TGC)3 | (TGC)3 | (TGC)3 | (TGC)3 | (TGC)3 | 9 | rpl22 | LSC |
| 52 | Trinucleotide | (TAT)3 | (TAT)3 | (TAT)3 | (TAT)3 | (TAT)3 | (TAT)3 | (TAT)3 | (TAT)3 | 9 | rpl22-rps19 | LSC |
| 53 | Trinucleotide | (TTA)3 | (TTA)3 | (TTA)3 | (TTA)3 | (TTA)3 | (TTA)3 | (TTA)3 | (TTA)3 | 9 | rps19 | LSC |
| 54 | Trinucleotide | (TCT)3 | (TCT)3 | (TCT)3 | (TCT)3 | (TCT)3 | (TCT)3 | (TCT)3 | (TCT)3 | 9 | ycf2 | IR |
| 55 | Trinucleotide | (CTT)3 | (CTT)3 | (CTT)3 | CTT)3 | (CTT)3 | (CTT)3 | (CTT)3 | (CTT)3 | 9 | ycf2 | IR |
| 56 | Trinucleotide | (GGT)3 | (GGT)3 | (GGT)3 | (GGT)3 | (GGT)3 | (GGT)3 | (GGT)3 | (GGT)3 | 9 | ycf2 | IR |
| 57 | Trinucleotide | (TGA)3 | (TGA)3 | (TGA)3 | (TGA)3 | (TGA)3 | (TGA)3 | (TGA)3 | (TGA)3 | 9 | ycf2 | IR |
| 58 | Trinucleotide | (ATT)3 | (ATT)3 | (ATT)3 | (ATT)3 | (ATT)3 | (ATT)3 | (ATT)3 | (ATT)3 | 9 | ycf2 | IR |
| 59 | Trinucleotide | (AGA)3 | (AGA)3 | (AGA)3 | (AGA)3 | (AGA)3 | (AGA)3 | (AGA)3 | (AGA)3 | 9 | ndhB | IR |
| 60 | Trinucleotide | (AGA)3 | (AGA)3 | (AGA)3 | (AGA)3 | (AGA)3 | (AGA)3 | (AGA)3 | (AGA)3 | 9 | ndhB | IR |
| 61 | Trinucleotide | (CTG)3 | (CTG)3 | (CTG)3 | (CTG)3 | (CTG)3 | (CTG)3 | (CTG)3 | (CTG)3 | 9 | rrn23 | IR |
| 62 | Trinucleotide | (GAA)3 | (GAA)4 | (GAA)4 | (GAA)4 | (GAA)4 | (GAA)4 | (GAA)4 | (GAA)4 | 12 | ycf1 | SSC |
| 63 | Trinucleotide | (AGA)3 |  |  |  |  |  |  |  | 9 | ycf1 | SSC |
| 64 | Mononucleotide |  | (A)15 | (A)15 | (A)15 | (A)15 | (A)15 | (A)15 | (A)15 | 15 | ycf1 | SSC |
| 65 | Trinucleotide | (ATT)3 | (ATT)3 | (ATT)3 | (ATT)3 | (ATT)3 | (ATT)3 | (ATT)3 | (ATT)3 | 9 | ycf1-rps15 | SSC |
| 66 | Trinucleotide | (GCT)3 | (GCT)3 | (GCT)3 | (GCT)3 | (GCT)3 | (GCT)3 | (GCT)3 | (GCT)3 | 9 | ndhA | SSC |
| 67 | Tetranucleotide | (GATT)3 | (GATT)3 | (GATT)3 | (GATT)3 | (AAT)3 | (AAT)3 | (GATT)3 | (GATT)3 | 12 | ndhA intron | SSC |
| 68 | Trinucleotide | (AAT)3 | (AAT)3 | (AAT)3 | (AAT)3 | (AGA)3 | (AGA)3 | (AAT)3 | (AAT)3 | 9 | ndhA intron | SSC |
| 69 | Trinucleotide | (AGA)3 | (AGA)3 | (AGA)3 | (AGA)3 | (TAG)3 | (TAG)3 | (AGA)3 | (AGA)3 | 9 | ndhA | SSC |
| 70 | Trinucleotide | (TAG)3 | (TAG)3 | (TAG)3 | (TAG)3 | (ATT)3 | (ATT)3 | (TAG)3 | (TAG)3 | 9 | ndhG-ndhE | SSC |
| 71 | Trinucleotide |  | (AAG)3 | (AAG)3 |  |  |  |  |  | 9 | ndhD-ccsA | SSC |
| 72 | Trinucleotide |  | (ATT)3 | (ATT)3 | (ATT)3 |  |  | (ATT)3 | (ATT)3 | 9 | rpl32-ndhF | SSC |
| 73 | Trinucleotide | (ATT)3 | (ATT)3 | (ATT)3 | (ATT)3 | (ATT)3 | (ATT)3 | (ATT)3 | (ATT)3 | 9 | rpl32-ndhF | SSC |
| 74 | Trinucleotide | (TAA)3 | (TAA)3 | (TAA)3 | (TAA)3 | (TAA)3 | (TAA)3 | (TAA)3 | (TAA)3 | 9 | rpl32-ndhF | SSC |
| 75 | Trinucleotide | (CTT)3 | (CTT)3 | (CTT)3 | (CTT)3 | (CTT)3 | (CTT)3 | (CTT)3 | (CTT)3 | 9 | ndhF | SSC |
| 76 | Trinucleotide | (TTA)3 | (TTA)3 | (TTA)3 | (TTA)3 | (TTA)3 | (TTA)3 | (TTA)3 | (TTA)3 | 9 | ndhF | SSC |
| 77 | Trinucleotide | (GAA)3 | (GAA)3 | (GAA)3 | (GAA)3 | (GAA)3 | (GAA)3 | (GAA)3 | (GAA)3 | 9 | ndhF | SSC |
| 78 | Trinucleotide | (CAG)3 | (CAG)3 | (CAG)3 | (CAG)3 | (CAG)3 | (CAG)3 | (CAG)3 | (CAG)3 | 9 | rrn23 | IR |
| 79 | Trinucleotide | (TTC)3 | (TTC)3 | (TTC)3 | (TTC)3 | (TTC)3 | (TTC)3 | (TTC)3 | (TTC)3 | 9 | ndhB | IR |
| 80 | Trinucleotide | (TCT)3 | (TCT)3 | (TCT)3 | (TCT)3 | (TCT)3 | (TCT)3 | (TCT)3 | (TCT)3 | 9 | ndhB | IR |
| 81 | Trinucleotide | (AAT)3 | (AAT)3 | (AAT)3 | (AAT)3 | (AAT)3 | (AAT)3 | (AAT)3 | (AAT)3 | 9 | ycf2 | IR |
| 82 | Trinucleotide | (TCA)3 | (TCA)3 | (TCA)3 | (TCA)3 | (TCA)3 | (TCA)3 | (TCA)3 | (TCA)3 | 9 | ycf2 | IR |
| 83 | Trinucleotide | (ACC)3 | (ACC)3 | (ACC)3 | (ACC)3 | (ACC)3 | (ACC)3 | (ACC)3 | (ACC)3 | 9 | ycf2 | IR |
| 84 | Trinucleotide | (AAG)3 | (AAG)3 | (AAG)3 | (AAG)3 | (AAG)3 | (AAG)3 | (AAG)3 | (AAG)3 | 9 | ycf2 | IR |
| 85 | Trinucleotide | (AGA)3 | (AGA)3 | (AGA)3 | (AGA)3 | (AGA)3 | (AGA)3 | (AGA)3 | (AGA)3 | 9 | ycf2 | IR |
